# A new fossil marine lizard with soft tissues from the Late Cretaceous of southern Italy

**DOI:** 10.1098/rsos.172411

**Published:** 2018-06-20

**Authors:** Ilaria Paparella, Alessandro Palci, Umberto Nicosia, Michael W. Caldwell

**Affiliations:** 1 Department of Biological Sciences, University of Alberta, Edmonton, Alberta T6G 2E9, Canada; 2 Department of Earth and Atmospheric Sciences, University of Alberta, Edmonton, Alberta T6G 2E9, Canada; 3 Dipartimento di Scienze della Terra, Sapienza Università di Roma, 00185 Rome, Italy; 4 College of Science and Engineering, Flinders University, GPO Box 2100, Adelaide 5001, Australia; 5 South Australian Museum, North Terrace, Adelaide, South Australia 5000, Australia

**Keywords:** Squamata, Pythonomorpha, Apulian Platform, Cretaceous, soft tissue, ultraviolet radiation

## Abstract

A new marine lizard showing exceptional soft tissue preservation was found in Late Cretaceous deposits of the Apulian Platform (Puglia, Italy). *Primitivus manduriensis* gen. et sp. nov. is not only the first evidence of the presence of dolichosaurs in a southern Italian Carbonate Platform, filling a palaeogeographic gap in the Mediterranean Tethys, but also extends the range of this group to the upper Campanian–lower Maastrichtian. Our parsimony analysis recovers a monophyletic non-ophidian pythonomorph clade, including *Tetrapodophis amplectus* at the stem of Mosasauroidea + Dolichosauridae, which together represent the sister group of Ophidia (modern and fossil snakes). Based on Bayesian inference instead, Pythonomorpha is monophyletic, with Ophidia representing the more deeply nested clade, and the new taxon as basal to all other pythonomorphs. *Primitivus* displays a fairly conservative morphology in terms of both axial elongation of the trunk and limb reduction, and the coexistence of aquatic adaptations with features hinting at the retention of the ability to move on land suggests a semi-aquatic lifestyle. The exceptional preservation of mineralized muscles, portions of the integument, cartilages and gut content provides unique sources of information about this extinct group of lizards. The new specimen may represent local persistence of a relict dolichosaur population until almost the end of the Cretaceous in the Mediterranean Tethys, and demonstrates the incompleteness of our knowledge of dolichosaur temporal and spatial distributions.

## 1. Introduction

Pythonomorpha (mosasauroids, dolichosaurs and snakes) is a clade including both extinct and extant squamates. While both snakes (Ophidia) and mosasauroids (Mosasauroidea) are recognized as monophyletic groups, dolichosaurs are typically reconstructed as a paraphyletic assemblage basal to mosasauroids [1–3]. The earliest fossil record of non-ophidian pythonomorphs dates back to the Early Cretaceous (Valanginian–Hauterivian) [4], while the latest discovery in non-marine deposits is reported from the late Campanian–early Maastrichtian of Spain [5]. By the Cenomanian–Turonian, non-ophidian pythonomorphs are found in marine deposits around the Mediterranean area, in western Europe, North America and possibly Australia [3,6–17], testifying to an ongoing radiation of these aquatic lizards at the beginning of the Late Cretaceous [15]. Of all non-ophidian pythonomorphs, only the more derived fully aquatic forms (Mosasauridae) survived up to the end of the Cretaceous, while aigialosaurs and dolichosaurs have so far been considered extinct by the Santonian [15].

Here, we present new data from an extremely well-preserved specimen, including soft tissue remains, of the first dolichosaur from the latest Cretaceous of southern Italy (Puglia), recovered from a new *Lagerstätte*-quality locality. This new finding not only fills a palaeogeographic gap in the Mediterranean Tethys for this group, being the first record from the Apulian Platform, but it also extends the range of dolichosaurs *sensu* Nopcsa [18] by about 10 million years (from the Santonian to the upper Campanian–lower Maastrichtian). The new taxon may well represent a Tethyan relict of its clade, a group that was presumed to be extinct much earlier in the Late Cretaceous. It also testifies to the survival of a quite conservative morphology (in terms of axial elongation and other aquatic adaptations) for marine non-ophidian pythonomorphs up to the late Campanian–early Maastrichtian. Moreover, the astonishing preservation of the soft tissues provides an unprecedented source of information to help us better understand the morphology of pythonomorphs and their interrelationships.

## 2. Material and methods

### 2.1. Specimen and images

The new specimen is housed at the Museum of Palaeontology of the ‘Sapienza’ University of Rome (MPUR, Museo Paleontologico dell'Università di Roma), Lazio, Italy. Natural light photos were taken using a Canon EOS 1000D digital single-lens reflex camera. A Nikon D3100 digital single-lens reflex camera was used for UV light photography. A Nikon Coolpix S3600 compact digital camera was used for dissecting scope photomicrography. Line drawings were made by hand using photographs of the material at both natural and ultraviolet light, and by direct observation of the specimen. Digitizing and figure construction were accomplished using Adobe^®^ Photoshop^®^ (outlines and colouring) and Adobe^®^ Illustrator^®^ (labelling and final production), both version CC 17 (2013 release).

### 2.2. Spectroscopic analysis

Scanning electron microscopy (SEM) using energy dispersive X-ray (EDX) microanalysis was performed on selected samples of cortical bone, muscles, gut contents and sediment in order to verify the composition of both hard and soft tissues, and to understand what factors might have led to such outstanding preservation. The samples were mounted on aluminium stubs using double-sided carbon tape, and examined with a SEM FEI Quanta 400 under low vacuum and uncoated (analysis time of 60** **s at 20 KeV) (see also electronic supplementary material).

### 2.3. Ultraviolet radiation

**Figure 1.**
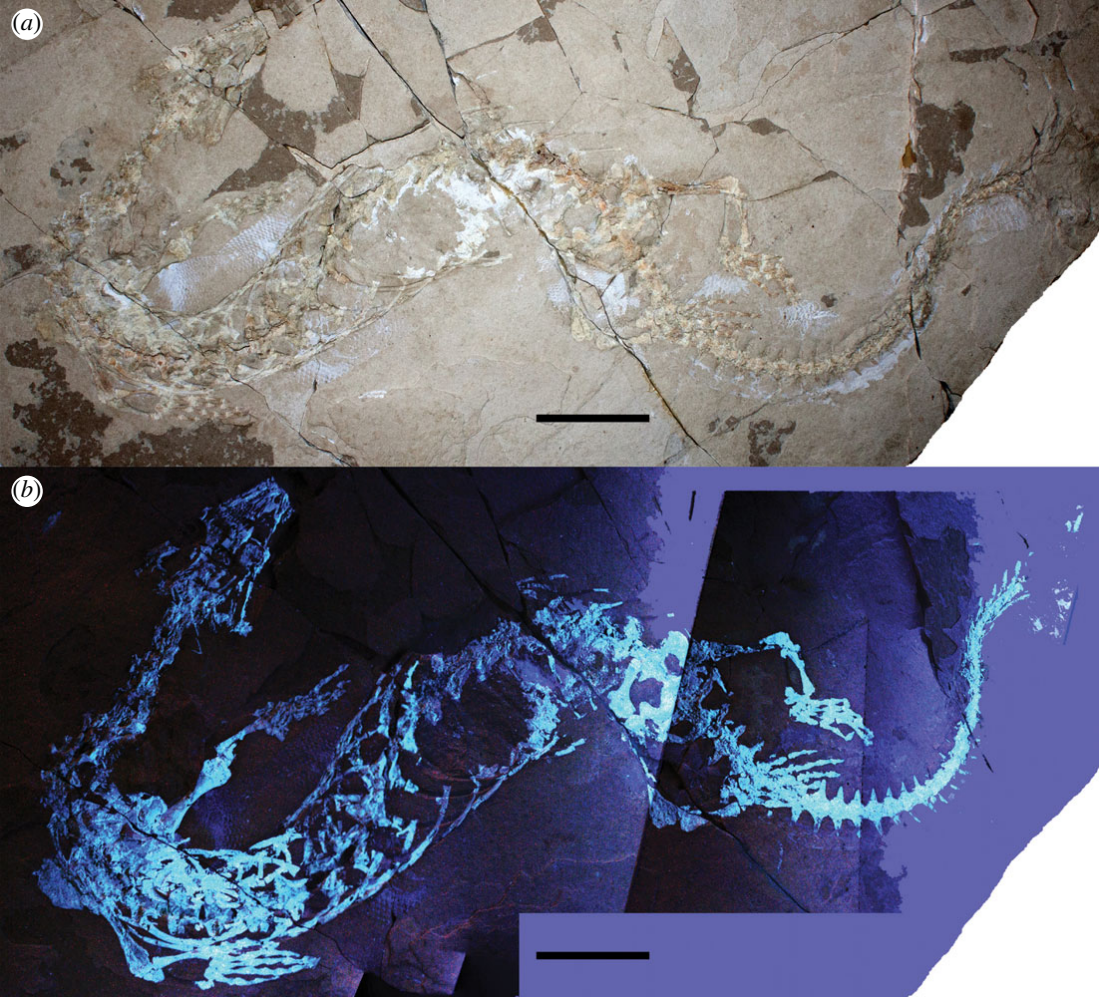
Holotype of *Primitivus manduriensis* gen. et sp. nov. (MPUR NS 161) at natural (*a*) and UV (*b*) light as exposed from the matrix in dorsal view. The imaging under UV radiations is a composite of two pictures, finalized with Adobe Photoshop CC 17 (2013 release). Scale bars: 5 cm.

**Figure 2.**
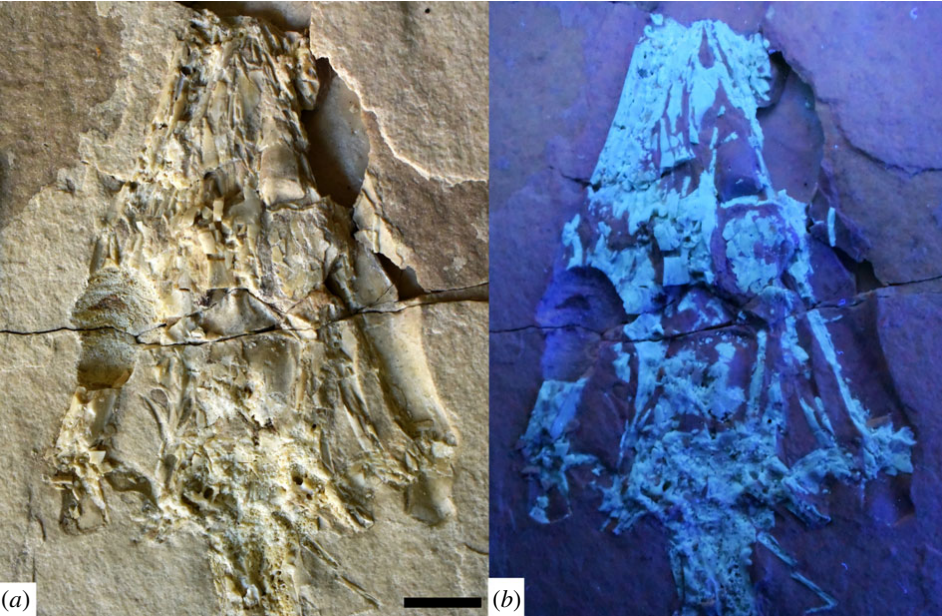
*Primitivus manduriensis* MPUR NS 161 imaging of the skull at natural (*a*) and UV (*b*) light. The skull of the holotype is heavily crushed (*a*), and part of the elements are only preserved as impressions on the matrix, as observed under UV light (*b*), where the bone material still preserved is bright white. Reconstruction and interpretation of the cranial skeleton are presented in electronic supplementary material (figure S2). Scale bar: 1 cm.

**Figure 3.**
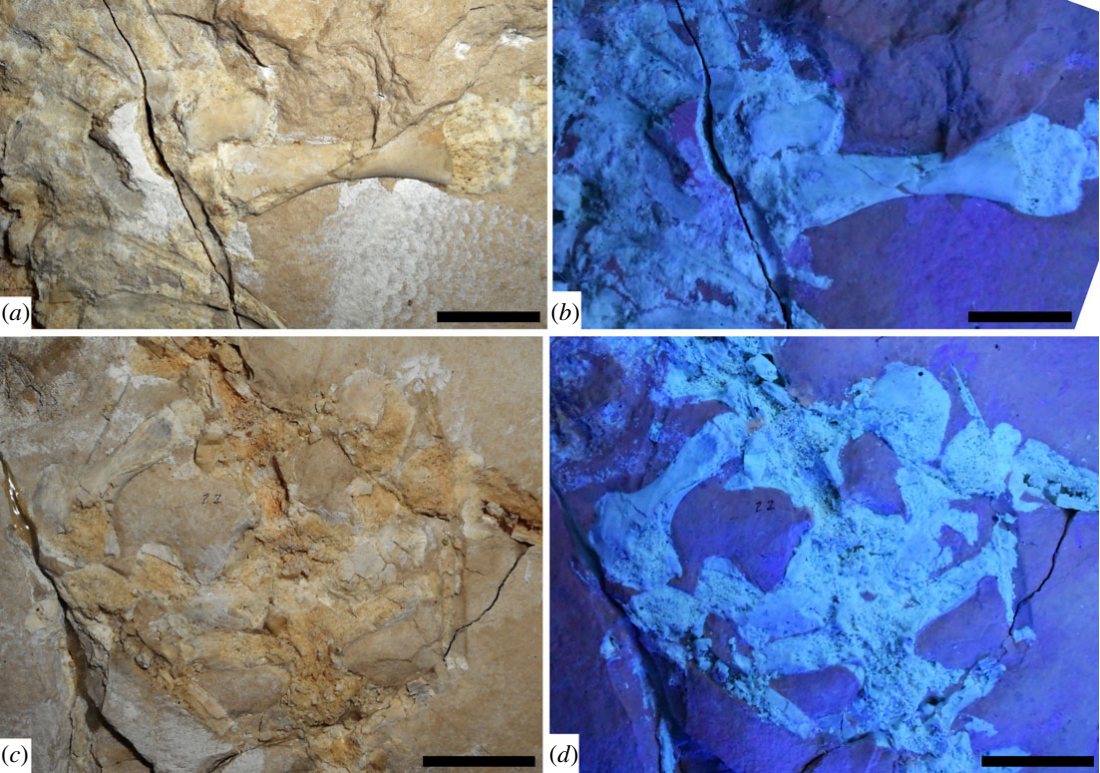
*Primitivus manduriensis* MPUR NS 161 imaging of pectoral and pelvic girdles at natural (*a*,*c*) and UV (*b*,*d*) light. As visible from the photographs under UV radiation, the pectoral region is extensively covered by cartilage (*b*), surrounding both right scapula and coracoid, up to the humeral proximal epiphyses, which are invisible under natural light. For the sacral region, the white material visible under natural light anteriorly to both ischia and first sacral rib becomes purple when exposed to the UV radiation, as well as the scales on both sides of the hips. Reconstruction of both pectoral and pelvic girdles is provided in electronic supplementary material (figure S4). Scale bars: (*a*,*b*) 1 cm; (*c*,*d*) 2 cm.

**Figure 4.**
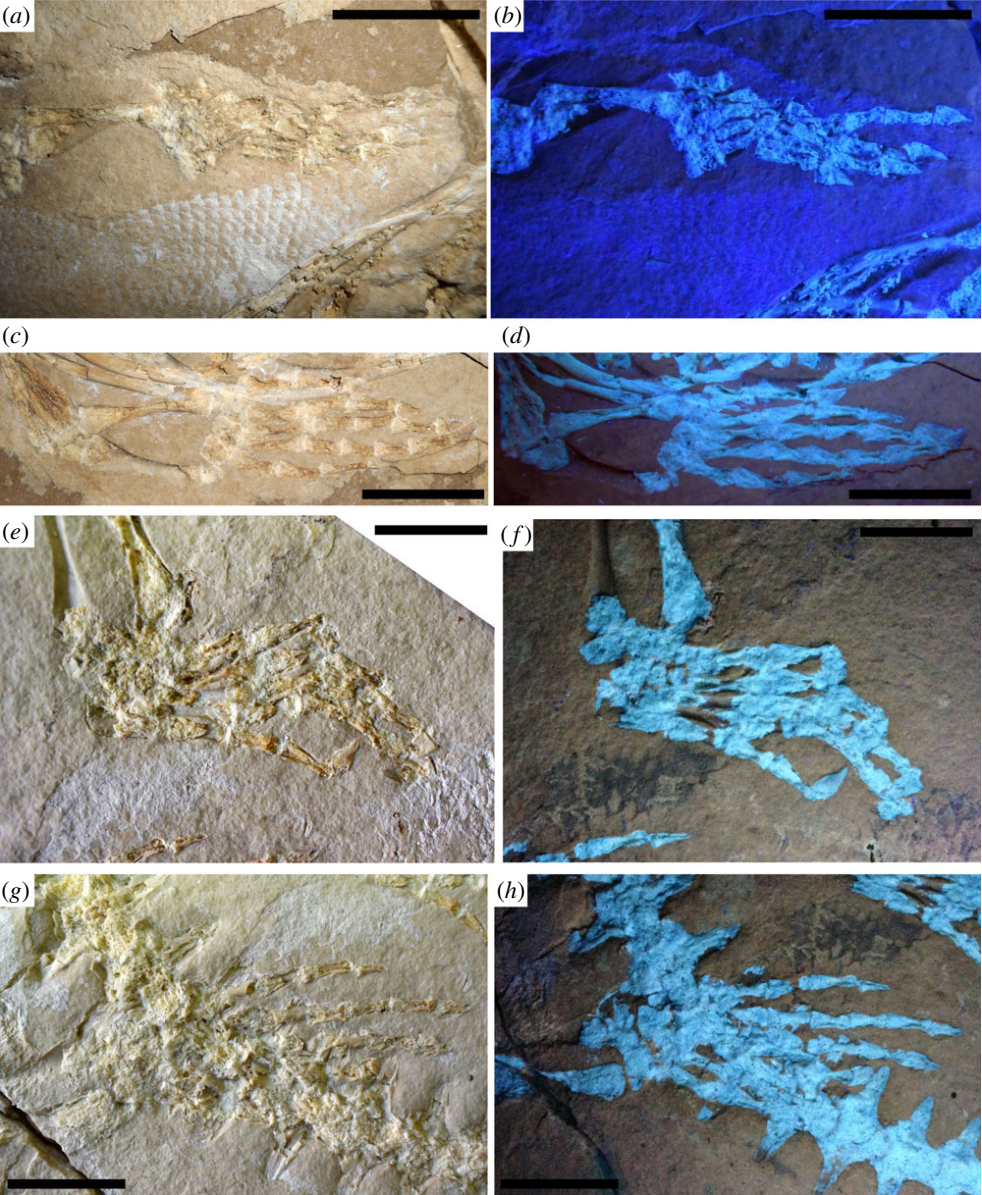
*Primitivus manduriensis* MPUR NS 161 imaging of appendicular elements at natural (*a*,*c*,*e*,*g*) and UV (*b*,*d*,*f*,*h*) light. Both manus and pes, as well as most of the limb bones are preserved and mostly articulated. The forelimb autopodium is pictured here as seen under both natural (*a*,*c*) and long-wave UV radiations (*b*,*d*), where the soft tissues are differentiated by a pink–purple colour range. For the hindlimb autopodium (*e*–*h*) the images are taken under short-wave UV radiations (*f*,*h*), with the soft tissues spanning a grey colour scale. Reconstruction of the limbs is presented in electronic supplementary material (figures S5–S8). Scale bars: 2 cm.

**Figure 5.**
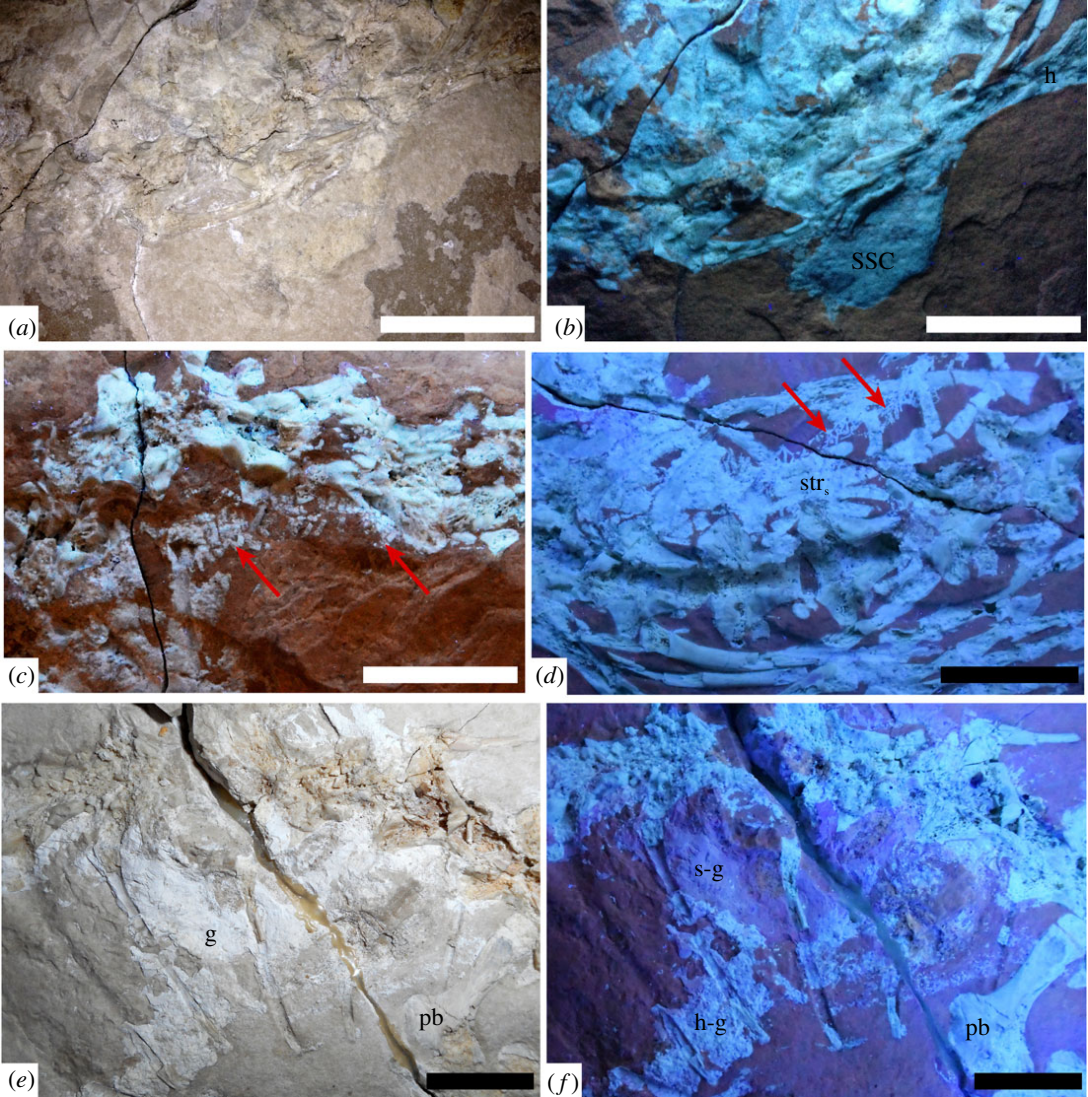
Cartilages and gut content preserved in MPUR NS 161. Cartilaginous elements, like suprascapula (*a*,*b*), tracheal rings (arrows in *c*), bronchial rings (arrows in *d*) and sternum (*d*) are also preserved in the specimen, and their assessment was possible owing to the use of UV radiations (*b*,*c*,*d*,*f*). For the gut content (*e*,*f*), under UV light it is possible to differentiate between a hard tissue component (emphasized in white) and a soft tissue component (emphasized in pink-purple). Scale bars: 2 cm. Abbreviations: g, gut content; h, humerus; h-g, hard tissue component in the gut; pb, pubis; s-g, soft tissue component in the gut; ssc, suprascapula; str_s_, sternal ribs.

**Figure 6.**
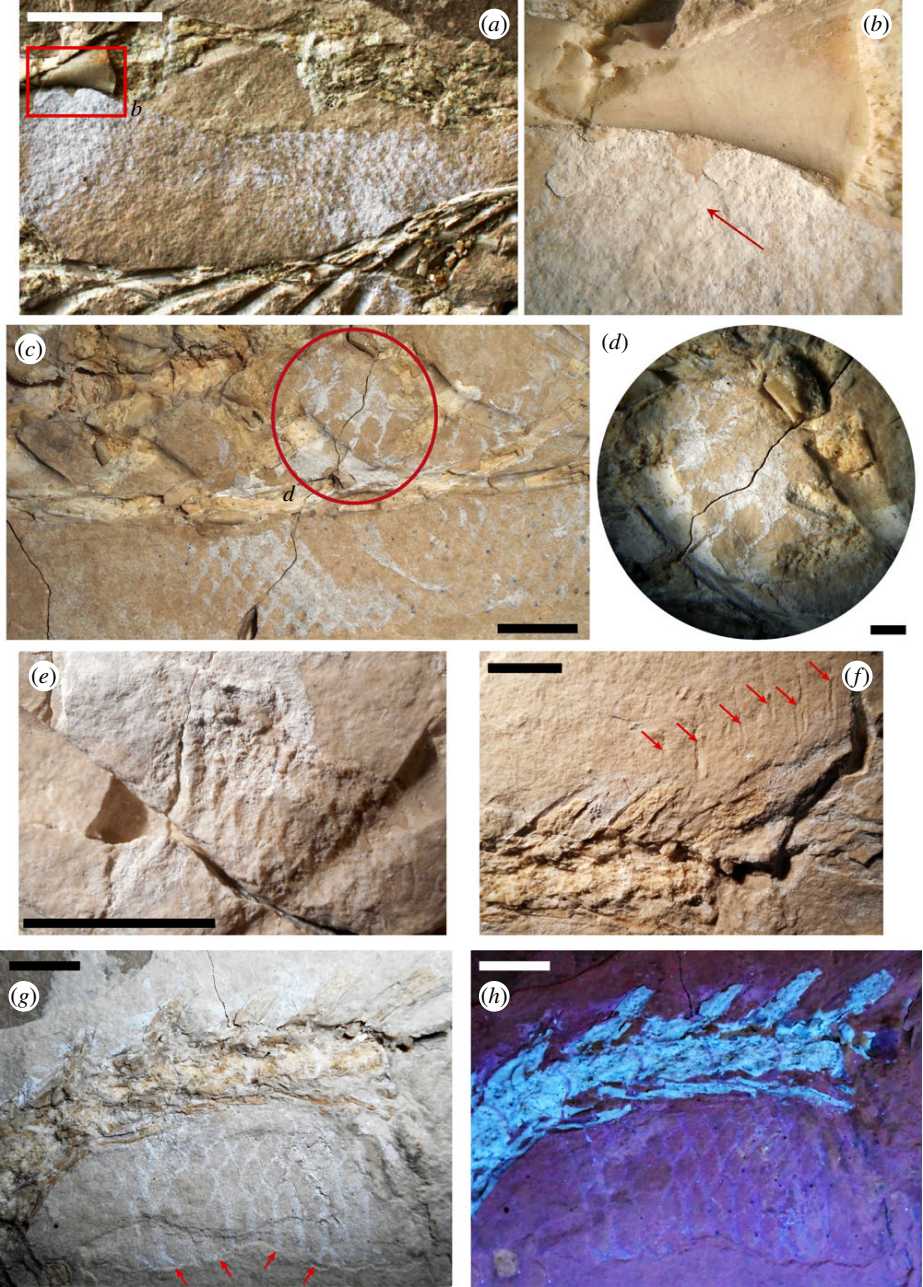
Different types of scales preserved in *Primitivus manduriensis*. Integument and scale impressions are present on both sides of the trunk (*a*–*d*), around the limbs (*e*), and along the tail (*f*–*h*). The different types of dorsal scales vary from polygonal (*a*,*c*,*e*,*g*,*h*) to diamond-shaped (*d*), but in the subcaudal region of the tail they are transversally elongated (*g*,*h*). Among extant squamates, transversally elongated belly or subcaudal scales are typical of snakes, while among fossils, similar scales are found in *Pontosaurus kornhuberi*. Arrows: *b*, pointing at the inner layer of the integument (dermis or hypodermis); *f*, pointing at scales of the caudal fin impressed on the matrix; *g*, pointing at body outline. Scale bars: (*a*) 2 cm; (*c*,*e*–*h*) 1 cm; (*d*) 5 mm.

Bones and matrix are of about the same colour under natural light; the distinction between preserved bone and moulds, as well as between soft and hard tissues, was facilitated by the use of ultraviolet (UV) radiation. The UV lamp used to analyse the specimen is a double-wavelength model that can radiate both short (254** **nm) and long (365** **nm) waves: the short waves highlight the different elements of the specimen in the grey spectrum of colours, while the long waves work in the scale of the colour purple (see figures 1–6; electronic supplementary material, figures S2, S6–S7, for further details). When exposed to UV light, the bony tissues (cartilage and bone) appear white, in high contrast to the coloured soft tissues (grey range with short waves, purple range with long waves). While the bones assume an off-white colour, the cartilaginous elements usually appear a brighter white, though they are mostly undistinguishable from the matrix under natural light. The contrast in colours is related to the presence of original phosphorus (P) (bone and cartilage = white range), and replacement P (soft tissues = pink–purple range) that replaces the original composition of both muscles and integument; from the SEM/EDX analyses we know that both hard and soft remains consist of calcium phosphate, interpreted as replacement calcium phosphate in the latter [19–21].

### 2.4. Phylogenetic procedures

To assess the phylogenetic position of MPUR NS 161, we added character scores to a modified version of the dataset of Palci & Caldwell [8]. The updated list of characters and other details about the results of our analyses are included in electronic supplementary material 2. Terminal taxa were modified to perform a mostly species-level analysis (except for *Adriosaurus* and *Aigialosaurus* scored as genera); all scorings are based on personal observation of the terminals. *Tetrapodophis amplectus* was also added to the data matrix, again with scorings based on personal observations (M.W.C. 2016). The final dataset consists of 27 taxa and 129 characters, with the anguid *Diploglossus millepunctatus* as the out-group. The data matrix was generated with Mesquite 3.04 [22].

#### 2.4.1. Parsimony

We performed both an equal-weight maximum parsimony (MP) and an implied weighting maximum parsimony (IWMP) analysis using TNT 1.5-beta [23–25]. The MP heuristic search was run using the tree-bisection-reconnection (TBR) algorithm, considered the best option for small datasets (27 taxa in our study) as per Goloboff *et al*. [23], with the number of maximum trees set to 99 999 and all the characters processed as unordered and unweighted. For the MP analysis, we applied the ‘Traditional Search’ to compute 1000 replicates of Wagner trees using additional random sequences and saving 10 trees per replicate; then, we employed a successive round of TBR branch swapping using trees from RAM, in order to increase the chance to find the actual shortest trees (see electronic supplementary material 2 and 3 for further information on analysis settings and outputs). Two optimal trees were retained after removing all the suboptimals, and the strict consensus topology is presented with relative supports in figure 6*a* (both optimal trees are included in electronic supplementary material, figure S11). Following Goloboff *et al*. [24,25], we also performed an IWMP analysis with K set to 3, and adopting the same steps described for the MP analysis. The IWMP resulted in a single optimal tree and the topology is presented in figure 6*b* (see also electronic supplementary material, figure S12). The nexus file used to run the analyses is provided as electronic supplementary material 3.

#### 2.4.2. Bayesian inference

The Bayesian inference (BI) analysis was performed with MrBayes 3.2.6 [26], under the Mk(V) model for variable characters [27]. As MrBayes does not handle polymorphic scorings, we converted all the polymorphisms in the dataset used to run the parsimony analysis into uncertainties using Mesquite 3.04 [22]. We used a gamma distribution for rate heterogeneity and treated the data as a single partition. We set generations to 10 000 000, frequency of sampling to 1000, burn-in fraction to 0.25 and the temperature parameter to 0.010 (which gave the best chain mixing values). We checked the optimality of the parameters for convergence and effective sample size from both MrBayes log file, and Tracer 1.6 [28]. LogCombiner [29] and Tree Annotator [30] were used to estimate the posterior tree (maximum clade credibility tree (MCCT)). The nexus file used to run the BI is provided as electronic supplementary material 4, and further details about the settings and outputs are included in electronic supplementary material 5.

## 3. Results

### 3.1. Geological aspects and age

The specimen was found near Nardò (Lecce, Puglia), a small town located in the Salento Peninsula (southern Italy) (electronic supplementary material, figure S1). This locality is particularly famous for its fossiliferous limestones containing abundant fossil fish remains [31,32]. The limestones are part of the informal geological unit ‘Calcari di Melissano’ (Cenomanian–Maastrichtian), which was deposited in a shallower portion of the inner lagoon of the Apulian Carbonate Platform [33]. The age of the limestone outcropping in the area of Nardò is considered to be upper Campanian–lower Maastrichtian based on nannofossils [31,32,34]. The specimen is preserved in a finely laminated (submillimetric laminae) carbonate mudstone that is light hazel in colour (figure 1). Spectroscopic analysis indicates that the carbonate is a Mg-rich calcite, i.e. dolomite (see also electronic supplementary material, figure S10). The macroscopic lamination results from small differences in the recrystallization of the mudstone into euhedral nanometric crystals (dolomitization process); the thickest lamina is 2** **mm thick and darker than the other laminae, suggesting hypoxic conditions at the sediment–water interface. Neither bioclasts nor microfossils are present in the sediment, and the only evidence of bioturbation is represented by one U-shaped tubular burrow (cf. *Terebellina*) preserved next to the specimen (figure 1*a*, top right). The densely packed laminae, the lack of microfossils and bioclasts, and the limited presence of bioturbation are consistent with deposition within anoxic to dysoxic waters in a tropical, semiarid environment.

### 3.2. Systematic palaeontology

Reptilia Linnaeus, 1758

Squamata Oppel, 1811

Pythonomorpha Cope, 1869

DOLICHOSAURIDAE Gervais, 1852

**Definition.** Dolichosauridae is here defined as the group including all taxa sharing a more recent common ancestor with *Dolichosaurus longicollis* than with *Aigialosaurus* sp. In our study, this includes the following genera: *Dolichosaurus*, *Pontosaurus*, *Primitivus* gen. nov., *Adriosaurus*, *Acteosaurus*, and *Aphanizocnemus* (cf. Nopcsa [18] and Conrad [35]).

**Diagnosis.** Dolichosauridae is here defined as the group of non-ophidian pythonomorphs characterized by the following combination of features: non-sutural contact between premaxilla and maxilla; jugal lacking large posterior process; postorbital portion of postfrontal + postorbital forming half or more of the posterior orbital margin; hypapophyses/hypapophyseal peduncles extending to the tenth presacral/precloacal vertebra or beyond (10–12 cervical vertebrae); 32–40 presacral/precloacal vertebrae; reduced scapula and coracoid; tail deep, laterally compressed (cf. Pierce & Caldwell [3], Caldwell [6,14], Palci & Caldwell [8]).

*Primitivus manduriensis* gen. et sp. nov.

**Etymology.** The genus is named after the famous red wine grape variety, ‘Primitivo’, native to and grown in great quantities in the Salento Peninsula (Puglia, southern Italy). The species name has been chosen to honour the full name of the wine, ‘Primitivo di Manduria’, which is not only produced around the town of Manduria (Taranto, Puglia), but also in other localities of the Salento Peninsula, including Nardò, where the specimen was found.

**Holotype**. MPUR NS 161, an almost complete skeleton mostly in articulation, exposed in dorsal view, partially embedded in the rock, and missing the terminal portion of the tail and some elements of the skull. Together with the skeleton, there are abundant soft tissues preserved, including permineralized muscle fibres and integument (figures 1–8; electronic supplementary material, figures S2–S9).

**Figure 7.**
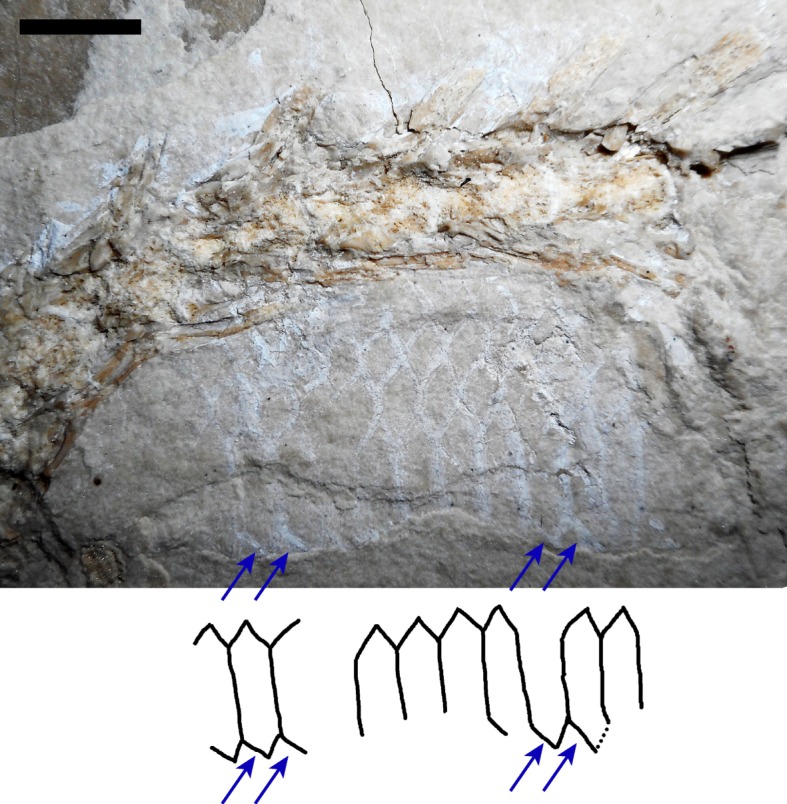
Close-up of *Primitivus* *manduriensis* MPUR NS 161 subcaudal scales. The proximal portion of the tail is exposed in dorsal view like the rest of the body, but at some point the tail rotates about 90° and the second part of the caudal column is exposed in left lateral view. Right after the torsion of the tail is where the transversally expanded ventral caudal scales (or subcaudals), similar to those of snakes, are visible. In snakes these scales can be present in a single row or as two adjacent rows; in MPUR NS 161, considering that this portion of the tail seems to be slightly twisted and compressed, we consider both interpretations as plausible. Scale bar: 1 cm.

**Figure 8.**
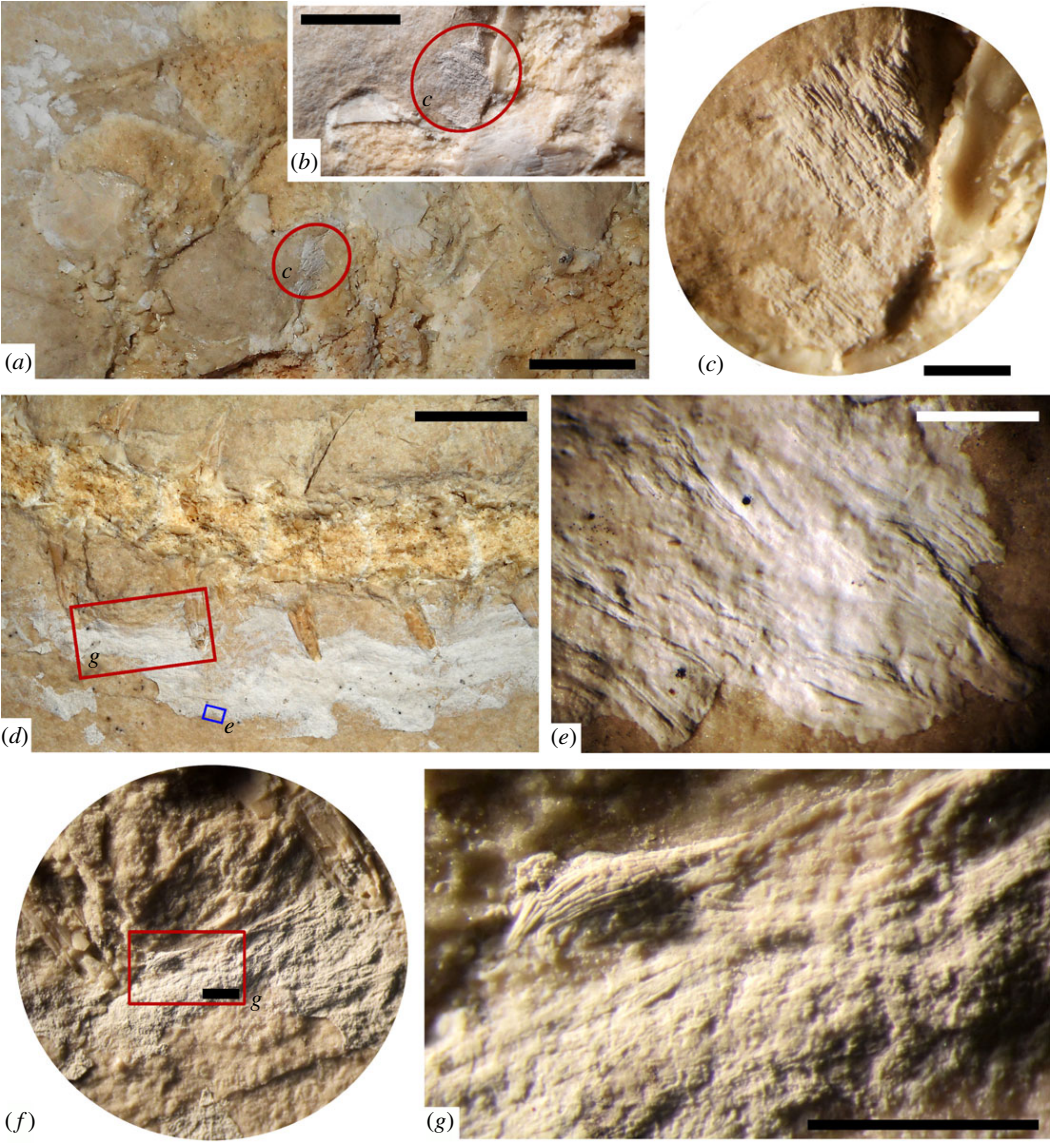
Mineralized epaxial and hypaxial muscles preserved in *Primitivus manduriensis*. Muscle fibres and bundles are well-recognizable along the posterior trunk, the pelvic girdle and the tail, even by the naked eye. Muscle fibres preserved between the first sacral vertebra and the left ischium (*a*–*c*) are about 30–35 µm in diameter. The muscle fibres along the anterior caudal region (*d*,*f*,*g*) are of the order of 10–15 µm. Under a compound microscope the single myomeres can in some instances also be distinguished (*e*). Scale bars: (*a*,*d*) 1 cm; (*b*) 5 mm; (*c*,*e*–*g*) 1 mm.

**Locality and stratigraphy.** Nardò, Lecce (Puglia, southern Italy); higher portion of the informal geological unit ‘Calcari di Melissano’, Apulian Carbonate Platform [31–34].

**Age**. Upper Campanian–lower Maastrichtian, based on microfossils [31,32,34].

**Diagnosis.** The new taxon can be distinguished from other dolichosaurids by the following unique combination of features: contact between frontal and prefrontal limited in the dorsal view; sutural contact between the septomaxilla anterolateral margin and the maxilla; the septomaxilla posterolateral margin in contact with the nasal; 10 cervical vertebrae + 22 dorsal vertebrae (32 presacrals); bowtie-shaped astragalus (with both a dorsal and a ventral notch); calcaneum with a proximal concavity for articulation with the fibula; deeply imbricated, small sub-circular scales on the lateral sides of the trunk and limbs; larger diamond-shaped scales on the trunk dorsal region; transversally expanded subcaudal scales.

### 3.3. Description

#### 3.3.1. Cranial skeleton

The skull is dorsoventrally crushed and exposed in dorsal view (figure 2; electronic supplementary material, figure S2). Many elements are fragmentary and preserved in part as impressions, as portions of the bones were lost with the unknown counterpart. This is the case for the parietal and frontal, both of which are preserved as impressions in the area adjacent to the fronto-parietal suture. The occipital region is badly crushed, and the cavities for the semicircular canals are partially exposed as the posterodorsal portion of the braincase (i.e. part of the otoccipitals) is missing. The limit between the basioccipital and the atlas is clear, and on the right side of the atlas, forming a 45° angle with its longitudinal axis, is a thin and long bone projecting posteriorly, that is part of the hyoid apparatus and most probably represents the first ceratobranchial. Both quadrates can be easily identified: the right quadrate is better preserved and almost complete; the left is mostly present as an impression on the matrix. On the right side, almost all the original contacts for the quadrate are preserved both dorsally (with the skull roof) and ventrally (with the pterygoid and the mandible). The anterior portions of both the lower and upper jaws are hard to differentiate, while posteriorly the mandibular elements are easier to recognize at least as impressions, with the retroarticular process being particularly well developed. The anterior portion of the skull preserves the septomaxillae and portions of the nasals and premaxilla.

The three large foramina on each side at the back of the braincase are most probably areas where the cavity of the inner ear (semicircular canals) is exposed due to the breakage and removal of the dorsal portions of the otoccipitals (electronic supplementary material, figure S2*a*). The anterior and posterior cavities correspond to coronal sections through the anterior and posterior semicircular canals, respectively; the largest and more medially placed cavity most probably represents a section through the crus commune, i.e. the portion of the inner ear where anterior and posterior semicircular canals meet dorsally. Between these foramina is a trapezoidal element that is probably a dorsoventrally flattened supraoccipital. Anterior to the semicircular canals, in what is probably a portion of prootic, a distinct foramen is visible on the right side, and is interpreted here as an opening for the VII cranial nerve (facial nerve). These identifications are based on braincase descriptions in Russell [36], Rieppel & Zaher [37], Bever *et al*. [38] and Head *et al*. [39]. Posterior to the otooccipital, the occipital condyle is partially exposed and its articulation with the atlas is visible as a slightly convex line (electronic supplementary material, figure S2*a*).

The parietal is very fragmentary, especially anteriorly, but a general outline of this bone can be resolved in the dorsal view on the left side, where the posterior process is broken but almost complete, and as an impression of its ventral surface on the right side (electronic supplementary material, figure S2*a*). The parietal table is broadly trapezoidal, while the posterolateral process (visible on the left side) is slender and triangular in dorsal view. It is not clear whether a large gap between the parietal and the supraoccipital represents the equivalent of the space for the *processus ascendens tecti synotici* or is an artefact of preservation. The fronto-parietal suture is preserved mostly as an impression, but it is clear that it was fairly straight, similar to that observed in aigialosaurs or even modern monitor lizards. The outline of the pineal foramen, located anteriorly in the parietal table, can be easily recognized, and anterior to it is a distinct mid-sagittal line that divides the left and right sides of the parietal. The posterior portion of the parietal, still mostly represented by bone, indicates that the bone was divided only anteriorly (parietal notch) as is typical of juvenile monitor lizards [40] and was not paired, an adult feature observed among extant lizards only within gekkotans [41]. The incomplete ossification of the left and right counterparts of the parietal can be interpreted as either a juvenile feature or a delay in ossification, a phenomenon common in aquatic forms (see Discussion).

On the right side of the skull, in front of the prootic and lying along the right side of the parietal table, is a rod-like element that we interpret as the epipterygoid, which must have rotated 90° due to the dorsoventral diagenetic compression of the skull.

The frontal is a very elongated unpaired element, much wider posteriorly at the suture with the parietal, then strongly constricted between the orbits and finally tapering anteriorly between the nasals. As in pontosaurs and coniasaurs, the posterior end is much more broadly expanded than the anterior one [3,6,13]. The posterior half of the frontal is mostly preserved as an impression, whereas anteriorly some fragments of the bone are still present, and the most anterior fragment (tip of the frontal) is located slightly anterior to the midpoint of the prefrontal. On the impression of the posterior ventral surface of the frontal, it is possible to recognize the natural mould of the olfactory canal (figure 2; electronic supplementary material, figure S2*a*). Whether or not the canal was completely enclosed by descending flanges of the frontal anteriorly cannot be determined.

Laterally and in front of the orbits, the frontal articulates with the prefrontals. A fragment of bone sandwiched between the frontal and right prefrontal may represent the posterior end of the right nasal. The extension of the impression of the premaxillary internarial bar suggests that originally it came in contact with the anterior tip of the frontal posteriorly (figure 2; electronic supplementary material, figure S2*a*).

A fragment of the left post-orbitofrontal is preserved on the left side of the skull. It clasps the fronto-parietal suture, forms the posterodorsal margin of the orbit and has a distinct squamosal ramus projecting posteriorly (electronic supplementary material, figure S2*a*). The squamosal ramus is cracked longitudinally, but its slender, distally tapering shape can nonetheless be inferred. Posterolateral to the squamosal ramus of the post-orbitofrontal is the impression of a pointed and slightly recurved element that must be the anterior portion of the squamosal. The impression can be followed posteriorly as it connects to a series of thin rod-like fragments.

Medial to the head of the right quadrate, and partially overlapping it, there is another rod-like element that we interpreted as a fragment of the right squamosal. This element can be followed anteriorly into a series of other fragments and impressions that together taper into a point, very similar in thickness and length to its left counterpart (electronic supplementary material, figure S2*a*).

The supratemporal is preserved on the left side as a small element inserted between the otoccipital and the posterior end of the squamosal. Its right counterpart can be identified in a similar position on the opposite side of the skull, where most of it is exposed due to breakage and displacement of the posterior end of the right squamosal (electronic supplementary material, figure S2*a*). The extension of the contact between the supratemporal and the quadrate seems to be greater than the contact between the quadrate and the squamosal, and also seems to prevent the contact between the quadrate and the paroccipital process (at least in the dorsal view).

Both quadrates are preserved in their articular position: the right one is quite complete and exposed in posterolateral view, whereas the left quadrate is present mostly as an impression of its medial face. The anterior outline of the quadrate is quite convex, and there is a distinct, posteriorly projecting suprastapedial process that is well preserved on the right quadrate. Details of the tympanic ala and tympanic crest cannot be resolved, but a distinct lateral conch is visible anterior to the suprastapedial process (figure 2; electronic supplementary material, figure S2*a*). Anterodorsally, the quadrate head is still in articulation with a rod-like element that probably represents a fragment of the squamosal (rather than the supratemporal); the rest of the dorsal–posterodorsal contact was probably occupied by the parietal ramus (which is missing in the right side of the skull, but is present on the left half), and then with at least one elongated element that projects farther more posteriorly than the quadrate itself, which we interpret as the supratemporal. This last bone seems also to prevent the contact between the quadrate and the paroccipital process, the position of which is indicated by the semicircular canal openings (electronic supplementary material, figure S2*a*). The right quadrate overlaps the posterior process (quadrate ramus) of the pterygoid, with which it is in contact medially. It is interesting to note that, according to the geometric relationship that the quadrates and mandibles have as compressed on the slab, the quadrates would turn to be almost vertical in lateral view, more similar to the condition seen in mosasaurs, aigialosaurs or even most iguanians, rather than, for instance, in varanids [2] (I.P. 2016, personal observation).

Only the right pterygoid is partially exposed in dorsal view, and part of it is only preserved as an impression (electronic supplementary material, figure S2*a*). The quadrate ramus consists of a robust, plate-like bone, posteriorly recurved towards the quadrate condyle (i.e. with the concavity facing laterally). The termination of the quadrate ramus does not taper significantly posteriorly, and terminates in a blunt, sub-rectangular end. On the left side the quadrate ramus is not exposed, but anteriorly a bone that could be interpreted as the ectopterygoid process is exposed in dorsal view, and forms the floor to the anterior portion of the orbit.

On the right side of the skull is a natural mould of the prefrontal, located just in front of the orbit; the element is rotated medially, so that what we see is the mould of its lateral and posterior walls. The left counterpart instead is not clearly identifiable among a mass of bone fragments. Owing to poor preservation, nothing can be said about the contact between the prefrontal and the maxilla. The contact between frontal and prefrontal appears to have been very limited, and must have occurred at the posterior end of the external naris. The lateral wall of the prefrontal tapers anteriorly, and has a straight lateral margin, while posteriorly it is both dorsoventrally deeper and mediolaterally wider. No indication of a lacrimal foramen or notch can be observed. The posterior wall shows a gently sinusoidal ventral margin, and a weak median concavity that is facing posteriorly, while its dorsal margin is smoothly rounded (figure 2; electronic supplementary material, figure S2*a*).

The premaxilla is missing, and only impressions and fragments of the internarial bar are visible between the two septomaxillae, and extending posteriorly towards the anterior tip of the frontal (electronic supplementary material, figure S2*a*). Close to the tip of the snout, two sub-triangular and paired elements are identified as the septomaxillae. The left septomaxilla is fractured, and only its medial portion is preserved. The right septomaxilla is complete, and its shape in dorsal view is extremely similar to that of *Coniasaurus gracilodens* [13]. The anterolateral margin shows a sutural contact with the maxilla, while posterolaterally it was at least in contact with nasal (electronic supplementary material, figure S2*a*): this sutural contact between the septomaxilla and the maxilla probably prevented the maxilla from moving independently of the rest of the skull (A.P. 2016, personal observation). The external naris must have been framed by the septomaxilla anteriorly, the medial margins of the maxilla and the prefrontal laterally, and the internarial bar of the premaxilla and the nasals medially, and must have terminated posteriorly in a tapering point where the prefrontal, nasal and possibly the frontal met.

Two paired elements, very narrow and pointed anteriorly, are identified as fragments of the anterior ends of the nasals, and are very similar in shape and topographical location to those of *Pontosaurus kornhuberi* [6]. A sub-triangular fragment at the posterior end of the external naris, between the prefrontal and the frontal, is also interpreted here as a fragment of the right nasal (posterior end); this is because of its shape, size, position and the presence of an impression in the sediment that connects this element to the anterior tip of the nasal described above. If this fragment is in its natural position, then the frontal may have been excluded from the posterior margin of the external naris by the nasals and the prefrontals.

Only fragments of the left and right maxillae are present. The left maxilla is the most complete, and appears to be preserved in dorsal view. The top portion has been sheared off and displaced medially, so that the canal for the second branch of the trigeminal nerve (V_2_) is exposed. Anteriorly, and located on the course of the canal for the above-mentioned nerve, is the section through a large tooth alveolus. Only one small tooth is preserved anteriorly on the maxilla. Just under the maxilla, and exposed only anteriorly, where a portion of the latter is missing, a fragment of the dentary can be observed. It bears one large tooth, complete with the root. Small fragments of dentary are also visible on the right side of the skull, but not much can be said about the general shape of this bone, except that it was probably extending posteriorly below the orbit.

Most of the lower jaws are preserved only as impressions (figure 2; electronic supplementary material, figure S2*a*). The condylar region appears to be exposed in dorsolateral view, as suggested by the shape of the retroarticular processes, but anteriorly the jaws are twisted somewhat more medially. On the impression of the right mandible, it is possible to identify one extensive suture line running anteroposteriorly, and starting in front of the condylar region: considering that the impression is that of the lateral face of the jaw, this suture is most probably that between the surangular and the angular (electronic supplementary material, figure S2*a*). On the left side this same suture can be observed dividing posterior fragments of the surangular and the angular. The retroarticular processes are preserved only as impressions, but it is possible to infer their size and shape very clearly. The retroarticular processes were broad and sub-rectangular, similar in shape and extension to those of *Pontosaurus* spp. and *Adriosaurus suessi* [3,6,42].

Scleral ossicles are visible inside both orbits, but an almost complete scleral ring is only visible on the right side. The shape of the individual ossicles cannot be established due to poor preservation, and a count of the number of elements is also not possible.

A jugal is not preserved, but the posterior extent of the orbit can be estimated by observing the posterior extension of the sclerotic ring. The orbits must have been anteroposteriorly elongate and relatively quite large, though not as large as in *Pontosaurus* (orbit diameter to skull length = 0.13; in *P. kornhuberi* = 0.19; in *P. lesinensis* = 0.19) (see electronic supplementary material, table S1, for measurements of the specimen).

As mentioned above, only two marginal teeth are preserved in association with the skull elements (electronic supplementary material, figure S3). They are both located towards the anterior end of the skull, and point in opposite directions. The most anterior of the two, missing the root and pointing laterally in dorsal view, is interpreted as a maxillary tooth; while the other one, almost complete and pointing medially, probably belongs to a fragment of the left dentary. Both teeth are conical, with no apparent lateral compression, and are slightly recurved posteriorly. The tooth crowns have multiple longitudinal facets separated by thin ridges (electronic supplementary material, figure S3*a*,*b*).

A slender and elongated element coming out from below the base of the skull, and exposed on the right side of the atlas and axis, is the first ceratobranchial (figure 2; electronic supplementary material, figure S2*a*). It is broken in two sections at mid-length and the posterior half is slightly displaced medially. This element terminates in a blunt end posteriorly, to which a thin rod of calcified cartilage is attached. The cartilaginous portion is preserved in a displaced position, and its longitudinal axis forms an angle of about 60° with that of the ossified portion of the ceratobranchial.

#### 3.3.2. Axial skeleton

Both vertebrae and ribs show some degree of pachyostosis, which is, *sensu stricto*, a thickening of the perichondral bone—as defined by Ricqlès & Buffrénil [43]. The mode of preservation of most of the skeleton facilitates the observation of the thicker walls (of vertebrae and ribs especially) and the brittle-like internal organization of the bony tissue. Unfortunately, without sectioning the bones it is not possible to verify if osteosclerosis had developed in the inner bone tissue [44–47].

Both the cervical and dorsal vertebrae are elongate, and roughly rectangular in shape, while the sacral and caudal vertebrae are shorter and more square (see measurements in electronic supplementary material, table S1). The dimensions of the vertebrae are, in general, quite different from typical ophidiomorph squamates, in which cervicals and dorsals are quite short relative to their width.

The precaudal vertebral column is complete, though for the tail the most distal part is missing. The boundary between cervical and dorsal series—as defined by Hoffstetter & Gasc [48]: the first dorsal vertebra bears the first rib pair that articulates to the sternum—can be determined due to some preserved costal cartilages (figure 5*d*). There are four well-preserved costal cartilages on the right side of the body, which although partially covered by dorsal ribs can be highlighted using UV light. The third of these costal cartilages still preserves its articulation with one of the ribs. By following a rib to its articulation with the corresponding vertebra, it is possible to determine that the latter must represent the third dorsal, and that therefore there are 10 cervical and 22 dorsal vertebrae. The atlas is identified as the shorter element articulating with the basioccipital condyle, just before the first cervical vertebra with a rib (i.e. the axis) (electronic supplementary material, figure S2*a*). The number of presacral vertebrae is very different from *Pontosaurus*, in which the number of dorsals is above 26, and in general from most ophidiomorphs that have a presacral vertebral count greater than 35 (39 in *Pontosaurus kornhuberi*) [3,6,7,49]. The presacral vertebral count in *Primitivus* is 32, just slightly higher than *Aigialosaurus dalmaticus*, where there are only 7 cervicals, followed by 22 dorsals in *Primitivus*, for a total of 29 presacral vertebrae.

The dorsal vertebrae in MPUR NS 161 are all broken through the neural arch or slightly ventral to it (figures 1 and 5). Their centra are cylindrical and robust, slightly expanded anteriorly and bear well-developed, laterally projecting synapophyses. The last dorsal bears a pair of very small ribs.

There are two sacrals, with the sacral ribs still in articulation with the posterior iliac blade. The first pair of sacral ribs is directed laterally, while the second pair is somewhat recurved anteriorly. The distal bony end of the second sacral rib bears an indentation on the posterior margin, which on both the left and right side is occupied by cartilage (figure 3*c*,*d*; electronic supplementary material, figure S4*b*). Such a morphology of the second sacral rib is similarly present in some iguanians, such as *Iguana* sp., *Agama* sp. and *Physignathus* sp., while in other lizards, such as *Gecko* sp., *Varanus* sp. and *Heloderma* sp., the distal bony margin of the second sacral rib appears quite square, and the posterior margin of the second sacral rib is nearly straight (I.P. 2016, personal observation). In the case of *Iguana* sp. or *Agama* sp., it looks like the posterior margin of the second sacral rib has a bony projection located about mid-length; in the case of MPUR NS 161 or *Physignathus* sp., this bony projection is located more distally, looking more like an indentation of the posterior distal corner of the sacral rib.

Posterior to the sacrals, there are several vertebrae with long distally tapering transverse processes that point laterally, but from about the tenth caudal vertebra the orientation of the transverse processes changes, becoming slightly posteriorly oriented. There are 19 caudal vertebrae preserved in dorsal view after the sacrum, then the tail rotates about 90° (between caudals 17 and 21) and the following caudals are exposed in left lateral view (figures 1, 6*g*,*h* and 7). The transverse processes are well developed in the first ten caudal vertebrae, then reduce in size between the 11th and 16th vertebra, where the tail begins to turn. After the curvature, there is no more evidence of transverse processes, although this might be due to preservation bias (these vertebrae are preserved in lateral view and the transverse processes may have broken off). In the portion of the tail exposed in lateral view, long haemal arches (or chevron bones) can be recognized between the 22nd and the 27th caudals (figures 6*g*,*h* and 7). After the 27th caudal vertebra, there is a gap due to matrix covering the specimen, followed by at least seven more caudals. In total, there are 37 caudals preserved, but considering that the last vertebrae present on the slab show no significant reduction in size, the tail was probably much longer. The chevron bones are slightly flattened against the vertebral centra, and most of them are disarticulated; their length is greater than the corresponding neural spines, at least for all the caudals preserved in lateral view (figure 6*g*,*h*). The caudal vertebral centra bear posteriorly a distinctive pedestal (haemapophysis) to which the chevron articulates, so the haemal arches are not fused to the haemapophyses, and the articular facet is posteroventrally oriented. Moreover, between two of the caudal vertebrae preserved in lateral view, it is possible to observe a zygosphene–zygantrum supplementary articulation (figure 6*h*).

The caudal neural spines in lateral view are inclined posteriorly about 45°, and narrow anteroposteriorly. Some scales preserved as both mineralizations and impressions in lateral view along the caudal region assist in determining the outline of the tail (figure 6*f*–*h*). The hypaxial portion is greatly dorsoventrally deepened in comparison to the epaxial portion; however, it is also clear that the epaxial portion of the tail must have extended dorsally beyond the neural spines. Indeed, along the dorsal edge of the tail, the scales impressed on the matrix indicate that there was some sort of caudal fin, similar to that of some modern sea snakes and sea kraits (e.g. *Hydrophis platurus*, *Laticauda colubrina*), or the water monitor, *Varanus salvator*. The width of the anterior portion of the tail, as inferred from the extension of the transverse processes, is quite remarkable, and is consistent with attachment for powerful caudofemoralis muscles. The depth of the posterior part of the tail suggests that it must have served as an excellent propelling organ during swimming.

Fragments of cervical ribs are preserved along the neck, but most of these ribs are either not fully exposed or too fragmentary to allow proper description. As was mentioned above, the limit between the neck and the trunk is recognizable owing to the preservation of four sternal ribs on the right side of the body, one of which (the third) retains its connection to one of the trunk ribs, which in turn is articulated to one of the vertebrae (the third dorsal vertebra).

All the ribs are single-headed. The proximal head is flared, and there is no neck-like constriction. The ribs have a thick, pachyostotic shaft, and taper distally towards the end, but expand again just before the tip, probably to form a surface for the attachment of a cartilaginous termination (figure 5*d*). The anterior dorsal ribs are uniformly recurved similar to *Pontosaurus lesinensis* or *Dolichosaurus longicollis*, and unlike *Pontosaurus kornhuberi* or *Mesoleptos zendrinii*, where the distal portion of the ribs is straight [3,6–8,11,14]. Some of the trunk ribs preserved as impressions on the slab also show a weak longitudinal groove, closer to the anterior margin of the shaft. The longest dorsal rib is about 81.5** **mm, which indicates that the trunk must have been fairly deep compared to other squamates, as can be expected in an animal adapted to swimming [50] (electronic supplementary material, table S1). Finally, in MPUR NS 161 there are five terminal dorsal ribs considerably shorter and straight in comparison to the rest of the thoracic series. This anatomy differs from that of mosasaurids where there is a much longer series of short presacral ribs, and is similar to more basal non-ophidian pythonomorphs where this feature can be observed. In *Pontosaurus kornhuberi* there are at least four shortened presacral ribs, while in *Adriosaurus* spp. the decrease in size is quite gradual, with the last two posterior dorsal ribs being significantly shorter. The condition is variable instead in aigialosaurs, because in *Aigialosaurus dalmaticus* all the presacral ribs are generally shorter and decrease in length gradually as in *Adriosaurus* spp., whereas *Aigialosaurus bucchichi* is similar to MPUR NS 161.

#### 3.3.3. Appendicular skeleton

With respect to *Pontosaurus kornhuberi*, *Acteosaurus tommasinii* and *Adriosaurus suessi*, the contrast in length between forelimbs and hindlimbs in MPUR NS 161 is not as pronounced, being more similar to the condition in both *Aigialosaurus* species. Following Palci & Caldwell [8], this can be quantified through the length ratios of the humerus and femur to the mean dorsal vertebra (mdv): the humerus to mdv ratio in MPUR NS 161 is up to 2.3, against a value of 1.3 in *Acteosaurus*, 2.0 in *P. kornhuberi*, between 1.6 and 2.2 in *Adriosaurus suessi*, and a similar value of 2.3 for *Aigialosaurus* spp.; for the femur to mdv ratio instead, the value in MPUR NS 161 is 2.9, slightly higher than that of both *Aigialosaurus* spp. (2.6–2.7) and *Acteosaurus* (2.7), and much lower in comparison to *P. kornhuberi* (3.3) and *Adriosaurus suessi* (3.3–3.6).

Only the right pectoral girdle is clearly recognizable on the skeleton as exposed, and this is partially overlapped by the ribs at the cervical–dorsal series transition, which are now lost but have left impressions on the surface of the bones (figure 3*a*,*b*; electronic supplementary material, figure S4*a*). Of the left pectoral girdle, only a large cartilaginous element is visible on the left side of the trunk, and this most probably represents the suprascapular cartilage (figures 1 and 5*a*,*b*).

The scapula and coracoid are single elements, and are not fused together. Under UV light, cartilage is identified in several places around both the scapula and coracoid (figure 3*a*,*b*; electronic supplementary material, figure S4*a*). Overall, the pectoral girdle is quite reduced in comparison to the rest of the body, a feature typical of pythonomorphs, and its morphology resembles very much the condition seen in *Carsosaurus marchesetti* and *Dolichosaurus longicollis*.

The scapula is hourglass-shaped, much smaller than the coracoid and with both ends about the same width. This is different from the condition identified, for instance, in *Adriosaurus skrbinensis*, and more similar instead to that of *Dolichosaurus longicollis*, *Carsosaurus marchesetti* and *Coniasaurus gracilodens*.

The coracoid is crescent-shaped, similar to that of *Aigialosaurus bucchichi* and *Carsosaurus marchesetti*. There seems to be no emargination on the anterior margin of the coracoid, while a scapulocoracoid fenestra seems to be present close to the glenoid fossa, as in *C. marchesetti*. The presence of a coracoid foramen cannot be confirmed, due to extensive cartilage material covering the median portion of the bone (epicoracoid cartilage), surrounding both the coracoid and the scapula, and probably in contact with the suprascapula (which is also preserved as cartilage).

The forelimbs are preserved pressed to the body and pointing posteriorly, so that the hand is exposed in ventral view (flexor aspect). The overall morphology of the humerus resembles that of *Carsosaurus marchesetti*, rather than either *Pontosaurus* or *Aigialosaurus*. The bone is hourglass-shaped but still quite elongated in comparison to the pectoral girdle elements and the presacral column, while in both *Pontosaurus* and *Aigialosaurus* the propodial is clearly shortened relative to the overall length of the limb (see measurements in electronic supplementary material, table S1). The distal end surface of the humerus is damaged, and the bone does not show the presence of either the ectepicondylar or the entepicondylar foramina. Although lack of an entepicondylar foramen is expected, as it is an autapomorphy of Squamata [51], lack of the ectepicondylar foramen may be preservational. The epiphyses are present on both humeri, but are not fully ossified, as observed when the skeleton is exposed to UV light (figures 3 and 4; electronic supplementary material, figures S4–S7).

The radius and ulna are best preserved on the left side. It is not possible to resolve their proximal epiphyses on either side of the body; however, on the left forelimb, distal unfused epiphyses (slightly disarticulated) are clearly visible on both bones. The two bones are close together proximally, contacting each other at the articulation with the distal margin of the humerus and then strongly diverge distally, although part of the divergence is artificial, because the ulna is no longer in articulation with the ulnare and its distal end is located dorsal to the pisiform (figure 4*a*–*d*; electronic supplementary material, figure S6). Divergent epipodials are also characteristic of other non-ophidian pythonomorphs, and are considered to be associated with an aquatic lifestyle [3,36,42,52,53]. The radius is a rod-like bone, slightly hourglass-shaped, with its posterior margin more prominently recurved than the anterior one; both its proximal and distal ends are only weakly enlarged in comparison to the thin shaft. The distal end has an oblique surface to which the distal epiphysis is attached. The ulna has a more evidently constricted shaft, with a more symmetrical anterior proximal end, and a posterior proximal end characterized by a distinct olecranon process.

In both fore- and hindlimbs the autopodium is much longer than the epipodial portion, consistent with the tendency of reduction of the proximal elements of the limb found in other non-ophidian pythonomorphs; however, in MPUR NS 161 this tendency is not as strong as in both *Pontosaurus* and *Aigialosaurus* for the propodials, even if the autopodial length is more than twice the length of the epipodials.

The right manus is very poorly preserved, so the following description is based on the left, which is complete, although surface preservation is not excellent (most of the perichondral bone has been sheared off) (figure 4*a*–*d*; electronic supplementary material, figure S6). In the left manus only the proximal carpals are readily recognizable, while, of the distal carpals, only the large fourth distal carpal can be seen close to the proximal end of the metacarpal IV.

The proximal carpals consist of a large square radiale, located between the epiphysis of the radius and metacarpals I and II; a large round central element (probably the lateral centrale), located postaxial to the radiale; a large oval ulnare, postaxial to the lateral centrale; and a small, comma-shaped pisiform, sandwiched between the ulnare and the distal epiphysis of the ulna (therefore, the distal end of the ulna is clearly disarticulated and shifted somewhat postaxially).

All metacarpals are hourglass-shaped (figure 4*a*–*d*; electronic supplementary material, figure S6). Metacarpal I is the shortest and broadest of the metacarpals, followed by metacarpal V, while metacarpal III is the longest.

The phalangeal formula for the manus is 2-3-4-5-3. The shape and size of all the phalanges (excluding the unguals) are similar in all digits with a flared proximal head and a less expanded distal condyle (electronic supplementary material, figure S6*b*). Most ungual phalanges are quite well preserved on both manus and pes, making up a distinct claw, posteriorly recurved and bearing two tubercles for attachment of the flexor musculature: a larger one located ventroproximally, and a smaller one located on the ventral margin (electronic supplementary material, figure S8).

All the unguals appear mediolaterally compressed and taper anteriorly into a blunted distal tip. On some ungual phalanges the articulation for the penultimate phalanx is also visible, and this facet appears slightly sinusoidal in the lateral view. On the dorsolateral surface of the ungual phalanges there are two foramina: one is located proximally, and the other one more distally, at about the mid-length of the dorsal margin leading to the distal tip of the ungual. Below this second foramen, running longitudinally along the tapering distal end of the ungual phalanges, there are some parallel grooves that do not reach the proximal end of the ungual (electronic supplementary material, figure S8).

The pelvic girdle is flattened on the slab and both sides are exposed in medial view, with the individual bones still in articulation or just slightly dislocated (figure 3*c*,*d*; electronic supplementary material, figure S4*b*). All the pelvic elements are preserved: pubes and ischia are complete, while the ilia are present part as actual bone and part as an impression in the matrix. Although tightly connected, the individual pelvic elements are not fused together.

In dorsomedial view, the ilium is characterized by an elongated and well-developed rod-like, posteriorly oriented process—here referred to as the posterior iliac or post-iliac process—and a long, thin anteroventrally oriented preacetabular process overlapping the pubis (electronic supplementary material, figure S4*b*). The ilium is still connected to both sacral ribs, and this articulation is visible on the medial aspect of the right post-iliac process, although most of the iliac process is preserved only as an impression. The contact between the second sacral rib and the ilium is intact, with a strip of cartilage completing the termination of the rib onto the posterior iliac process (figure 3*c*,*d*; electronic supplementary material, figure S4*b*). The posterior end of the post-iliac process is blunt in the mediolateral view, and on the left side it partially overlaps the left transverse process of the first caudal (pygal?) vertebra. The presence of the anterodorsally oriented supra-acetabular process, found in many terrestrial lizards (e.g. varanoids, iguanians) as well as mosasaurids, cannot be verified due to preservational factors: both left and right anterior iliac portions are flattened against the head of the femur, and their dorsal margin, where the supra-acetabular anterior iliac tubercle might be, is not clearly exposed. The two facets for articulation with the pubis and the ischium on the iliac head have about the same length: the right ilium is still weakly articulated to the ischium more posteriorly, while the left ilium is still articulated to the pubis but only partially with the ischium.

The left pubis is particularly well preserved, with a broad proximal head that is greatly expanded posteriorly in lateral view (resulting in the typical hatchet-like shape for this bone) (figure 3*c*,*d*; electronic supplementary material, figure S4*b*). The distal end of the right pubis is hidden underneath the last dorsal vertebra, and only its proximal head remains visible. Owing to the poor preservation of the bony surface, it is not possible to determine the location or presence of a pubic foramen. The anterior pubic process (or tubercle) is very inconspicuous, and appears only as a swollen eminence along the anterior margin of the proximal head of the pubis, not far from the acetabulum. The articular facet for the ilium occupies most of the dorsal and posterodorsal margin of the pubis in medial view; while the facet for the ischium is located posteriorly on the pubic head. The ventromedially directed pubic shaft is significantly narrower than the proximal head, and ends distally in a square termination, quite weakly expanded; still attached to the distal end, there is also a fragment of cartilage that is most probably part of the pubic symphysis. On the medial side of the pubic shaft, there is a well-preserved and dorsoventrally elongated, teardrop-shaped surface for the attachment of muscle tissues: considering the position (medial view), the surface was probably for the insertion of the *musculus puboischiofemoralis internus* [54] (figures 3*c*,*d* and 5*e*,*f*; electronic supplementary material, figure S4*b*).

The ischium is the shortest element of the hip. Both ischia are slightly dislocated from their original position in connection with the other pelvic bones, and are partially covered by the sacral vertebrae and ribs (electronic supplementary material, figure S4*b*). The ischium is strongly recurved along its anterior margin, and its proximal head is narrower than the distal end. The ischial expansion opens posteriorly right below the ischial neck to form a steep angle and then continues along the posterior margin of the ventromedially directed shaft, almost until the distal end. The distal termination of the ischium, where the element would contact its counterpart along the midsagittal plane, is fairly straight and at least twice the size of the distal extremity of the pubis. The proximal head of the ischium articulates with the pubis along an anterior facet, and with the ilium along its dorsal margin.

Unlike in *Aigialosaurus* spp., in MPUR NS 161 the femur is still quite long relative to the axial skeleton, and the morphology of the pes is not significantly modified in comparison to a terrestrial lizard, as observed in *Pontosaurus* [3,6,7]. However, the epipodials in the hindlimbs, similar to those described for the forelimbs, are strongly divergent distally, a feature that is associated with swimming [3,42].

As in the humerus, the epiphyses of the femur are not completely ossified (figure 3*c*,*d*; electronic supplementary material, figure S5*b*). The shaft of the femur is quite long and robust, almost twice the length of the epipodials (electronic supplementary material, table S1). The proximal epiphyses are partially overlapped by the anterior portions of the ilia. The proximal head of the femur is expanded, but less than the distal end; a gently rounded condyle for articulation with the pelvic girdle is visible at least on the left femur, especially when the element is exposed to UV light. On both sides of this condyle, in posterior view, there are two weak trochanters, with the internal one being slightly lower than the external (figure 3*c*,*d*; electronic supplementary material, figures S4*b* and S5*b*). For the articulation with the tibia and fibula, the distal femoral end in posterior view bears a more prominent mesial (tibiofibular) condyle, and a less prominent but wider lateral (tibial) condyle.

As a result of the flattening of the hindlimbs, with most of the axial skeleton visible in dorsal view, both pairs of epipodials are exposed on the slab in posterolateral view (electronic supplementary material, figure S5*b*). The epiphyses of both the tibia and fibula are not fully ossified, nor fused to the diaphyses. Tibia and fibula are in close contact proximally, at the articulation with the femur, but then diverge distally, as described for the radius and ulna.

The tibia is more robust than the fibula, and its proximal head is larger than the distal end (electronic supplementary material, figure S5*b*). On the right tibia, both medial and lateral condyles can be recognized, and still in contact with the articular cartilage of the distal end of the femur. The distal end of the tibia is fan-shaped, and articulates with both the astragalus and a small preaxial element that is identified as part of the tibial epiphysis. The tibial shaft is strongly constricted at mid-length and its internal margin is more prominently recurved than the external one.

The fibula is preserved mostly as an impression on both sides (only proximal and distal extremities are represented by bone). It is more gracile than the tibia, and both proximal and distal terminations are about half as wide as those of the latter bone (electronic supplementary material, figure S5*b*). The internal articulation of the left fibula with the medial condyle of the tibia is particularly well preserved, showing the close proximal contact of the two bones. The distal end of the fibula is somewhat irregular, due to the greatly expanded articular cartilage, and it contacts both the calcaneum (distally) and the astragalus (preaxially).

The mesopodium of MPUR NS 161 is one of the most complete found in dolichosaurs (figure 4*e*,*f*; electronic supplementary material, figure S7). Both distal and proximal rows of tarsals are preserved on the hindlimbs, though better exposed in the right pes, which is not overlapped by the tail. The proximal row consists of a bowtie-shaped astragalus, and a trapezoidal calcaneum, which shows a weak proximal concavity for articulation with the fibula (electronic supplementary material, figure S7*b*). The two bones are clearly not fused together. The astragalus articulates proximally with the epipodials, and postaxially with the calcaneum.

In the right pes, distal to the astragalus and calcaneum, a large centrale and four distal tarsals are preserved. The first three distal tarsals increase gradually in size postaxially, while the fourth is the smallest (electronic supplementary material, figure S7). There is no fifth distal tarsal. The centrale, which lies between the astragalus and the fourth distal tarsal is a large trapezoidal element, roughly as large as the calcaneum.

In all metatarsals the epiphyses are poorly ossified, especially distally (figure 4*e*–*h*; electronic supplementary material, figure S7). Metatarsals I and V are shorter and stouter in comparison to the elongated and slender metatarsals II to IV (electronic supplementary material, table S1). Metatarsal I is the only element of the metapodium with a distal termination that is smaller than the proximal one, and it also has a greatly recurved preaxial margin. Metatarsal IV is the longest element of the metapodium, and like metatarsals II and III, is characterized by a relatively thin shaft. Metatarsal V is broadly expanded proximally, and slightly hooked.

Common to both metatarsals and phalanges is the presence of a flexor groove on the ventral aspect: this structure is visible on the distal half of the autopodial elements of the right pes that are preserved as impressions in the matrix (figure 4*e*,*f*; electronic supplementary material, figure S7).

The phalangeal formula for the pes shows the typical primitive condition seen in lepidosaurs, corresponding to 2-3-4-5-4, so there is no reduction of the fifth digit, contrary to what is observed in aigialosaurs and mosasaurs. The overall shape of the phalanges of the pes is similar to that of the same elements in the manus: they have a broad square proximal end, a constricted shaft and a smaller distal end (electronic supplementary material, figure S7*b*). The terminal phalanges consist of well-developed claw-like unguals, and the same description given for the ungual phalanges of the manus also applies to the pes (electronic supplementary material, figure S8).

#### 3.3.4. Cartilage

With the use of UV light, it was possible to distinguish between bone and cartilage, and even identify cartilaginous elements that are not readily visible under natural light. These elements include: tracheal and bronchial rings, calcified sternal ribs, epicoracoid and suprascapular cartilages, and all the unossified appendicular epiphyses (figure 5). On the right side of the neck, starting from the fifth cervical vertebra—at the beginning of the bend in the neck—a set of tracheal half-rings are visible under UV light (figure 5*c*). Unlike *Pontosaurus kornhuberi*, where complete rings are preserved, in MPUR NS 161 we only see incomplete rings, which appear as narrow rod-like fragments of cartilage compressed against the cervical vertebrae. The rings disappear next to the eighth cervical, where the cartilaginous portion of the pectoral girdle covers up most of the right side of the anterior region of the trunk. Under UV light, beneath both the ribs and vertebrae of the anterior trunk, several cartilaginous elements are observed (figure 5*d*). These include both the cartilaginous sternal ribs and the bronchial rings. The bronchial rings are well exposed along the right side of the trunk: they appear as tiny rod-like fragments among the anterior dorsal ribs, and extend anteriorly up to a point just posterior to the right coracoid. Four pairs of cartilaginous sternal ribs, each formed by at least two segments, can be recognized, and are especially evident on the right side of the body. The sternal ribs can be distinguished from the other trunk ribs because of their greatly expanded distal ends. The third cartilaginous rib still retains its articulation with one of the dorsal ribs (articulating with the 13th presacral vertebra). On the left side of the trunk, close to the proximal head of the left humerus, another broad cartilaginous element was UV-illuminated, which we interpreted as the suprascapula (figure 5*a*,*b*). The element is trapezoidal in shape, with the distal margin greatly expanded. Under UV light, the epicoracoid cartilage appears preserved in connection with the coracoid on the right side, and surrounds most of the scapula (figure 3*a*,*b*; electronic supplementary material, figure S4*a*). This cartilage was crushed underneath some trunk ribs and the rest of the pectoral girdle; it is hard to tell if the right suprascapula is preserved; however, the cartilaginous portion visible along the anterior margin of the scapula is quite extensive, so it is likely that the epicoracoid cartilage and suprascapula were in contact. With regard to the appendicular skeleton, all the epiphyses are unossified: the lack of ossification becomes more evident when the bones are exposed to UV radiation and the epiphyses are emphasized in a much brighter white colour, in comparison to the diaphysis, indicating a contrast in density and mineralization (figures 1–4; electronic supplementary material, figures S4–S7).

#### 3.3.5. Integument

Different types of squamation are recognizable together with the skeletal remains. Even accounting for some post-mortem flattening of the body during compaction of the sediments, the scales appear to be preserved in their original position (figures 1 and 6). However, the external morphology of the scales is not preserved anywhere along the body, suggesting that it is not the outer layer of the epidermis (keratinized stratum corneum) that is preserved, but rather an inner layer of the integument, resulting in the reproduction of the scale outlines (here referred to as ‘scale ghosts’) and not the actual epidermal scales.

Deeply imbricated, small sub-circular scale ghosts are visible on both sides of the specimen next to the trunk and limbs (figure 6*a*–*e*). Between the right forelimb and the trunk, the permineralized integument follows the bend of the anterior portion of the skeleton, so that the scales look compressed against each other, highlighting the position of the original body outline. These small scales must have covered the sides and part of the belly of the animal, at least from the pectoral girdle to the sacral region (figure 6*c*). Larger diamond-shaped scales are preserved between the trunk ribs, and in particular on the left side of the posterior trunk region (figure 6*c*,*d*). These scales most probably covered the dorsal region of the body, because they appear to overlap the ribs (where present) and are abruptly interrupted where the bone is missing leaving only natural moulds. Diamond-shaped scales are also present in lateral view along the tail, where in the ventral region there are at least four rows of rhomboidal scales before the beginning of the broader, transversely expanded, subcaudal scales. Dorsal caudal scales are also preserved, but are not as clear as the subcaudals (figure 6*g*). Impressions of rhomboidal scales are clearly visible above the neural spines of the caudals in lateral view (figure 6*f*), suggesting the presence of a fin-like dorsal expansion along the top of the tail, probably similar to that of modern sea snakes and sea kraits (e.g. *Hydrophis platurus* and *Laticauda colubrina*), or the water monitor, *Varanus salvator*. One of the most important features characterizing the new marine lizard is the presence of transversally expanded scales, visible along the ventral margin of the last preserved caudal vertebra, where the tail is exposed in left lateral view (figures 1, 6*g*,*h* and 7). Among extant squamates such scales are typical of some snakes [55], while among fossils a similar squamation has been found in another basal pythonomorph, *Pontosaurus kornhuberi* [6,7]. Lee & Scanlon [55] and Lee [2] refer to these scales as subcaudal scales—or simply subcaudals—and in snakes they can be present as singles (i.e. one row) or pairs (i.e. two adjacent rows). In MPUR NS 161, either the tail is exactly in the left lateral view, with the scales visible along the ventral edge representing part of the right-side counterparts (like the paired condition in snakes), or due to twisting and compression of the soft tissues—as suggested by the irregularity of the tail base outline—the long scales represent complete, transversally expanded ventral scales (like the single condition in snakes). According to this second interpretation, the partially exposed scale ghosts along the ventral edge would belong to the other flank (right) of the tail (figures 6*g*,*h* and 7). The subcaudals in MPUR NS 161 are anteroposteriorly shorter than the caudal centra, and the length of one centrum corresponds to that of about two scales, similar to *Pontosaurus kornhuberi*; in snakes this ratio is highly variable. The scale ghosts are preserved owing to permineralization of the integument, or as impressions in the sediment. Being only the outline of the scales present, it is difficult to determine if the scales were smooth or keeled, as in the mosasauroids *Tylosaurus proriger* and *Ectenosaurus clidastoides*, or in *Pontosaurus kornhuberi* [6,56,57]. In MPUR NS 161, the most complete scales are found in the posterior trunk region and around the hindlimbs (figure 6*c*–*e*), but even in these cases the permineralization of the soft tissue does not allow full assessment of their original external morphology. Finally, in some parts of the specimen there are extensive portions of permineralized soft tissues where no distinct squamation is recognizable. Here the scale ghosts are not discernible, and there is no fibre-like arrangement either, suggesting that no mineralized muscle fibres are present: what is exposed may be the dermis (with the epidermal layer being degraded) or even the superficial fascia (or hypodermis), which lies between the integument and the external musculature (figure 6*a*,*b*).

#### 3.3.6. Muscles

Portions of permineralized epaxial and hypaxial musculature are preserved along the trunk and tail (figures 1, 5*e*,*f* and 8). Collagen fibres and muscle bundles are visible around the pelvic girdle and in close association with the anterior caudal vertebrae. To assess the identity of the muscles in MPUR NS 161, we made comparisons with studies on the musculature of several squamates [54,58–62]. On the right lateral posterior region of the trunk, where the body curves, small portions of permineralized muscles are preserved between the last dorsal ribs (figure 5*e*,*f*), with the fibres being obliquely oriented relative to the vertebral column. These muscles must have been part of the more internal layers, because they attach to the lateral surface of the vertebral centra and seem to arise from the ventrolateral aspect of the trunk (hypaxial musculature), and are overlapped by both ribs and vertebrae. Based on their position, they may be part of the *musculus* (*m*.) *obliquus internus*, or of the *m. transversus abdominis* [60,62]. More muscle tissue is visible along the anterior margin of the ischia: these muscle fibres are so well preserved that the single myomeres can be easily distinguished with the naked eye (figures 3*c*,*d* and 8*a*–*c*). Considering that the hips are visible in medial view, and that the muscles appear to attach to the anterolateral surface of the ischia, most probably they represent a mineralized portion of the *m. puboischiofemoralis externus*, which usually originates from the lateral surface of the ischium and inserts onto the proximal portion of the shaft of the femur [54,58]. Along the anterior caudal region, before the tail bend, a large portion of permineralized muscle tissue is preserved on the left side of the vertebral column (figure 8*d*–*g*; electronic supplementary material, figure S9). This part of the tail is exposed in dorsal view, and according to the relationship between vertebrae and soft tissues, and the changes in orientation and organization of the muscle fibres, it is possible to identify at least two different types of fascicles probably belonging to different epaxial muscles: (i) anteroposteriorly oriented, thin and tubular-like fibres overlapping the transverse processes of some caudal vertebrae, interpreted as part of the *m. transversospinalis* (figure 8*d*,*f*,*g*); (ii) bundles of broad and flat muscle sheets, positioned more laterally than the previous type (further away from the vertebrae), and oriented obliquely relative to the long axis of the body, interpreted to be part of the *m. iliocaudalis* [54,61,62] (figure 8*d*,*e*).

#### 3.3.7. Gastric and gut contents

In the posterior trunk region, near the transition to the sacrum, a large mass of permineralized soft tissue is preserved that is not observable under natural light (figures 1 and 5*e*,*f*). Under UV light, some parts of this mass acquire a pink-to-purple colouring, typically assumed by the soft tissues, but most of the mass remains white, indicating the large presence of bony material in the gut (figure 5*f*). Several tiny, rod-like fragments of bones are visible under UV light, and although their identity cannot be clearly assessed, this suggests that the animal was feeding on small vertebrates (e.g. fish). More anteriorly, on the left side of the mid-trunk, a small bone is visible between the dorsal ribs of the specimen (figure 6*d*): the small and slightly recurved element does not seem to be consistent with the rest of the skeleton, because its shape and size do not match those of any other bones from this body region, and it is clearly overlapped by a trunk rib. Considering its position in the trunk, and its etched surface, we can confidently interpret this element to be a partially digested bone (possibly from a fish) that was present in the stomach at the time of death.

## 4. Discussion

### 4.1. Taphonomy

**Figure 9.**
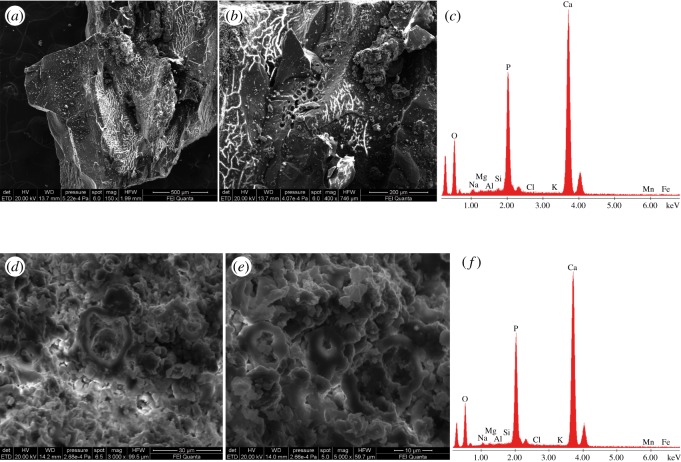
Results of SEM/EDX analyses for bony and soft tissues. Samples of hard and soft tissues have been selected in order to find their composition: a fragment of cortical bone from a trunk rib (*a*–*c*), and a sample from the muscles preserved along the trunk (*d*–*f*). Results for additional samples from the sediment and the gut content are reported in electronic supplementary material, figure S10. From the two spectra (*c*,*f*) it is clear that both bony and soft tissues have been permineralized with calcium phosphate, presenting the same composition. By comparison with Cosmidis *et al*. [68], the spherical forms visible in (*d*) and (*e*) can be interpreted as the presence of fossil bacteria. Bone vascularization is also perfectly preserved as visible on the bone fragment (*a*), and the diameter of the blood vessels is between 3.5 µm and 5 µm.

The spectra resulting from the SEM/EDX analyses of the soft tissues, bones and sediment are presented in figure 9, and electronic supplementary material, figure S10. Both bony and soft tissues are rich in phosphorus (P), and display a very similar composition, while there is no P in the embedding sediment. The soft tissues have been permineralized with calcium phosphate and thus preserved. According to Briggs [20], the replication of the original morphology of the soft tissues, resulting in exceptional preservation, is dependent upon rapid authigenic mineralization due to the steep geochemical gradients generated by microbes associated with decomposition. The mineralization of the soft tissues is not bacterially controlled but can be bacterially induced, because bacterial decay contributes to establishing the conditions for the retention of high concentrations of P during fossilization [21]. Two requirements have been proved to co-occur for the permineralization of soft tissues: reducing conditions in the surroundings of the carcass to slow decay, and the establishment of environmental isolation, either physical or chemical [19,20,63,64]. After such conditions are established, the availability of P ions is fundamental, and sources can be internal (from the animal's decay), or even external (other decaying organisms releasing P into the micro-environment) [63,65,66]. The establishment of an anoxic environment and a drop in the pH of the micro-environment around the specimen would inhibit the precipitation of calcium carbonate from the surrounding sediment, and the abundance of P would favour instead precipitation of calcium phosphate to permineralize hard and soft tissues [19,63,65,67]. In our specimens there are signs of at least partial decay of the carcass, as the outer layer of the epidermis (i.e. the keratinized stratum corneum) is not preserved: the integument is found in multiple areas of the body, but not perfectly preserved, and is lacking where the muscle fibres are more extensively exposed, suggesting that decomposition had started to some extent (figures 1 and 6). The permineralization of the soft tissues happened at the ‘expense’ of internal sources of P, such as bony tissue and possibly muscle fibres [20,65]: the P was first trapped and then re-used, in order to permineralize both integument and muscles. While the integument was greatly affected by decomposition, we see that muscles are almost perfectly replicated in three dimensions, where preserved: this should be related to both the fact that the muscle fibres are a source of the ‘recycled’ P themselves, and that the sediment burial had prevented the degradation to penetrate deep into the carcass. The lack of interaction between the carcass and the sediment—i.e. release of P ions from the carcass into the sediment—which is suggested by the lack of P in the surrounding matrix (see electronic supplementary material, figure S10) must be related to the formation of a film around the internal sources of P produced by bacteria (figure 9*d*,*e*: cf. Cosmidis *et al*. [68]). Films are accretions of bacteria that concentrate and attach to a surface, usually at a solid–liquid interface, producing in the process a substance matrix [69]. Looking at the way the abdominal region of the specimen is preserved, with some of the posterior trunk ribs missing or poorly impressed on the matrix, it is possible that gaseous rupture of the body also occurred before the carcass was completely covered by the sediment (figures 1 and 5*e*,*f*). However, due to the lack of evidence of scavenging, the great degree of skeletal articulation and the preservation of abundant soft tissues, we believe that only a short time passed between the death of the animal, the floating phase, sinking and burial after landing on the seafloor. At that point, the microbial film (with bacteria related to the initial decay and possibly internal gut bacteria [20,70]), together with the sediment cover, established anoxic to dysoxic conditions in the micro-environment surrounding the corpse, triggering the process that led to such exceptional preservation [21,63]. The fact that the spectroscopic analysis found the same composition for both the skeletal elements and soft tissues, coupled with the complete lack of P in the surrounding sediment, corroborates the diagenetic hypothesis above.

### 4.2. Phylogeny

**Figure 10.**
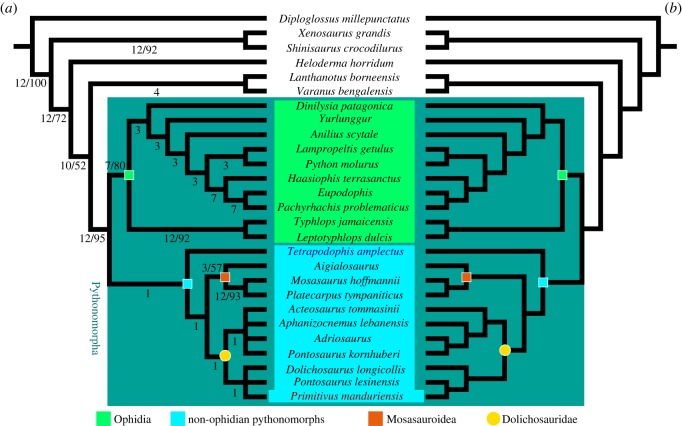
*Primitivus manduriensis* phylogenetic relationships based on parsimony. Phylogenetic hypotheses on the interrelationships of the new taxon are based on equal-weight maximum parsimony (*a*) and implied weighting maximum parsimony (*b*).

**Figure 11.**
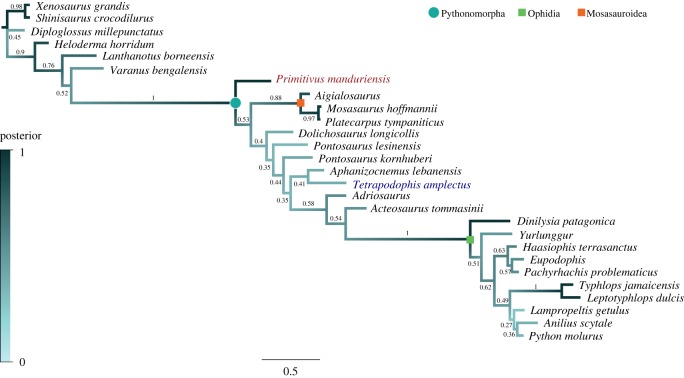
*Primitivus manduriensis* phylogenetic relationships based on Bayesian inference. The MCCT resulting from our model-based analysis recovers the new taxon as basal to all other pythonomorphs. The gradient colour of the branches is based on values of the posterior probabilities (numbers on each branch).

Based on anatomical comparisons, we identified *Primitivus* as a non-ophidian pythonomorph, and assessed its phylogenetic relationships using an updated version of the dataset of Palci & Caldwell [8] (electronic supplementary material 2–3). The main difference between the results of the MP and IWMP analyses centres on the resolution of the resulting trees, greater in the IWMP (see also electronic supplementary material 2). Final topologies for both MP (strict consensus of two optimal trees) and IWMP (single optimal tree) agree in recovering a monophyletic Pythonomorpha, with snakes (Ophidia) as the sister group to the clade *Tetrapodophis* + Mosasauroidea + Dolichosauridae (i.e. non-ophidian pythonomorphs) (figure 10). In the parsimony analysis, *Tetrapodophis* is recovered at the stem of a monophyletic mosasauroids + dolichosaurs grouping, and although its placement with the other non-ophidian pythonomorphs is poorly supported by Bremer and bootstrap values on a relatively long branch, it is consistent in both MP strict consensus and IWMP optimal tree (figure 10). The BI analysis offers instead a different scenario (figure 11): Pythonomorpha is still monophyletic, but *Primitivus* is recovered as the sister taxon to all other pythonomorphs which consists of Mosasauroidea + Ophidiomorpha *sensu* Palci & Caldwell [49]. In the model-based topology, *Tetrapodophis* is more deeply nested as the sister taxon to *Aphanizocnemus* and together they represent the sister clade to *Adriosaurus* + (*Acteosaurus* + Ophidia).

A monophyletic Dolichosauridae, as recovered in our parsimony analysis, includes most of the taxa traditionally assigned to the family by Nopcsa [18]—i.e. *Dolichosaurus*, *Pontosaurus*, *Adriosaurus* and *Acteosaurus*—with the addition of *Aphanizocnemus*, already placed within Dolichosauridae by Conrad [35]—and the new taxon *Primitivus*. The different placement of *Primitivus* between the model-based and parsimony-based topologies can be interpreted as consistent with our observations based on its anatomy. The new specimen shares numerous similarities with *Pontosaurus*, and in particular with *P. lesinensis*, as we emphasize in the description, and this is reflected in the MP and IWMP trees (figure 10). However, it also displays some conservative traits in terms of aquatic adaptations that make it comparable to both aigialosaurs and dolichosaurs (see description), and this justifies the results of the BI (figure 11). *Primitivus* may well represent an early-diverging pythonomorph, maintaining a more conservative body plan (e.g. limited axial elongation, poorly modified paddle-like limbs) in contrast to more derived forms such as obligatory aquatic mosasauroids or even greatly elongated adriosaurs and snakes. Its persistence until the end of the Cretaceous was probably favoured by the relatively isolated conditions of the Apulian Platform (see discussion about palaeobiogeography and palaeoecology below).

Unlike previous phylogenies [6,8], our results suggest that the genus *Pontosaurus* is not monophyletic, with *P. kornhuberi* forming a clade with *Adriosaurus* (as the sister taxon in the IWMP optimal tree), *Aphanizocnemus* and *Acteosaurus*, while *P. lesinensis* is grouped with *Primitivus* (its sister taxon in the IWMP topology) and *Dolichosaurus* (figure 10). Although *P. kornhuberi* shares several anatomical features with *P. lesinensis*, details of the anatomy reveal a closer affinity with *Adriosaurus*. These include a fused postorbital and postfrontal (separated in *P. lesinensis*), a wider parietal table (reduced to a thin midsagittal crest posteriorly in *P. lesinensis*) and pachyostotic ribs that are straight distally (uniformly recurved in *P. lesinensis*).

Ophidia is recovered as monophyletic in both model-based and parsimony results, as the sister group of *Tetrapodophis* + (Mosasauroidea + Dolichosauridae) in the MP and IWMP trees, and as sister clade to *Acteosaurus* in the MCCT (figures 10 and 11). In the parsimony-based topologies, the two extant taxa *Typhlops* and *Leptotyphlops* are sister taxa and together form the sister group to the rest of the snakes included in our phylogeny; the Upper Cretaceous snakes (*Dinilysia*, *Pachyrhachis*, *Haasiophis*, *Eupodophis*) are part of a clade with *Yurlunggur* (Oligo-Miocene [71]), and the extant taxa *Anilius*, *Lampropeltis* and *Python*. The South American fossil snake *Dinilysia* represents the most basal member of this latter clade, while the Middle Eastern taxa *Pachyrhachis*, *Haasiophis* and *Eupodophis* are more deeply nested, as the sister group to modern macrostomatan snakes, *Lampropeltis* + *Python*. The phylogenetic placement of the hind-limbed pachyophiids (*Pachyrhachis*, *Haasiophis* and *Eupodophis*) as the sister group to modern macrostomatan snakes is consistent with their skull morphology (adaptation for large gape), while their retention of well-developed hindlimbs would suggest that these have been independently reduced in snakes such as *Anilius* and *Python* (or more unlikely that pachyophiids re-evolved some distal limb elements). In the BI topology, *Pachyrhachis*, *Haasiophis* and *Eupodophis* form a clade that is the sister group to the modern taxa, while *Dinilysia* and *Yurlunggur* occupy the most basal branches of Ophidia (figure 11). Importantly, in this topology scolecophidians (*Typhlops* and *Leptotyphlops*) are no longer placed at the base of Ophidia, but are nested above all fossil forms.

The oldest known snake is Middle Jurassic in age [72], while non-ophidian pythonomorphs seem to make their first appearance in the Lower Cretaceous [4]. This means that the divergence between the ophidian and non-ophidian lineages within Pythonomorpha happened in, or is older than, the Middle Jurassic, and the longer branch leading to the non-ophidian pythonomorph clade in the parsimony-based topologies or the longer branch leading to Ophidia in the model-based tree can be probably shortened by including the earliest snakes, and hopefully more complete specimens of early dolichosaurids (e.g. *Kaganaias hakusanensis*).

### 4.3. Ontogeny and lifestyle

**Figure 12.**
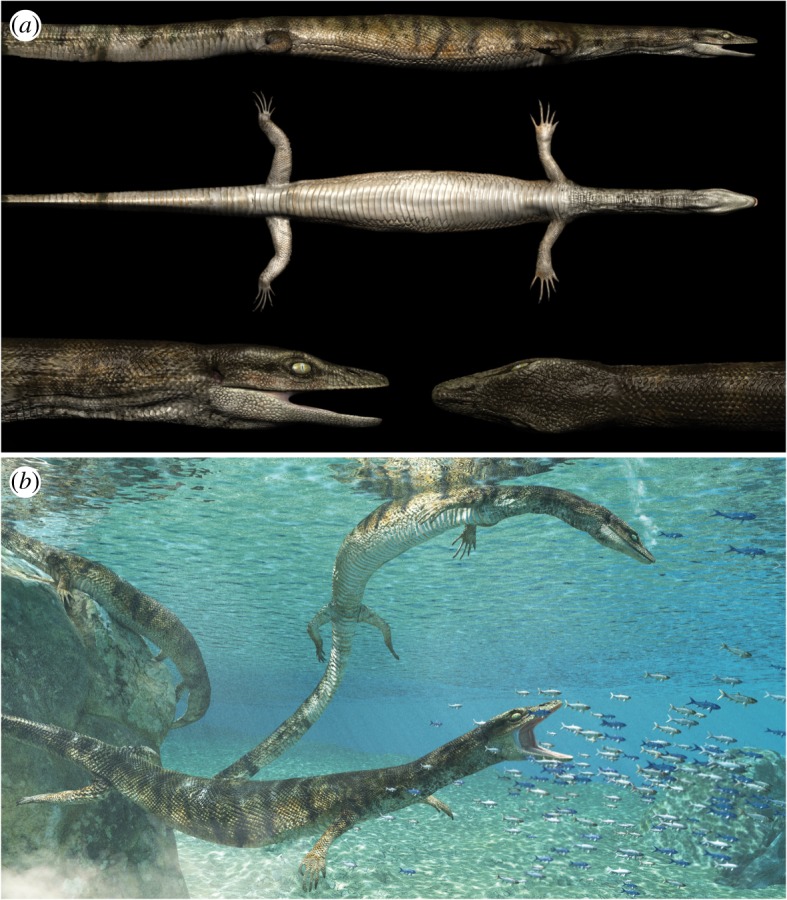
*Primitivus manduriensis* three-dimensional model and life reconstruction. The specimen is preserved in sediments deposited in the shallower portion of an inner lagoon of the Apulian Carbonate Platform, and is inferred to have a semi-aquatic lifestyle. Three-dimensional model (*a*) and life reconstruction (*b*) created by Fabio Manucci.

Because secondary adaptations to an aquatic lifestyle often lead to reduced or delayed ossification in the limb bones [73], in aquatic animals it is not always straightforward to separate juveniles from adults. The reduced ossification observed in the limbs of *Primitivus* is an example of this problem. Characters such as the unossified bony epiphyses, unfused epiphyses or unfused hip elements can all be interpreted either as indicative of an earlier ontogenetic stage [74], or as the retention of paedomorphic traits linked to adaptations to an aquatic lifestyle [75,76]. The final interpretation relies upon the combination of these morphologies with other relevant characters that instead are not affected by a similar dualistic explanation. The presence of an open parietal notch, with the parietal table apparently divided in two halves anterior to the parietal foramen, is a typical juvenile feature in different groups of lizards [40]. This character suggests that the specimen most probably represents a sub-adult. On the other hand, features like the elongated neck (increased number of cervical vertebrae, as well as an elongated cervical centrum), the reduced pectoral girdle, distally diverging epipodials on both fore- and hindlimbs, the elongated autopodium (phalanges long and slender) and the laterally flattened tail (tail much taller than wide, emphasized by the presence of the scaled caudal fin) are all indicative of aquatic adaptations. Another important aspect to consider in order to reconstruct the lifestyle of *Primitivus* is the morphology of the sacral region. *Primitivus* retains a functional sacrum, preserving a firm connection between the pelvic girdle and the sacral vertebrae, similar to other dolichosaurs. The terrestrial-like configuration of the articulation between the posterior process of the ilium and the two sacral ribs together with the configuration of the limbs suggests that this lizard was probably still capable of moving about on land, and was not obligatorily aquatic like mosasaurs (figure 12).

### 4.4. Palaeobiogeography and palaeoecology

Despite the possession of more derived traits such as the increased number of cervical vertebrae and reduced ossification of both axial and appendicular elements, *Primitivus* displays a low degree of axial elongation in the trunk region in comparison to most other non-mosasauroid pythonomorphs, possessing only 22 dorsal vertebrae, for a total of 32 presacrals. Moreover, we do not see in the new specimen an evident reduction of the forelimbs as in most dolichosaurids, and the hindlimbs clearly retain a more terrestrial-like configuration (similar to *Pontosaurus* spp.). *Primitivus manduriensis* may represent a relict form that survived until the latest Cretaceous in an isolated area of the Mediterranean Tethys, or may just be the first evidence of a more diverse and long-lived dolichosaur fauna. As suggested by Citton *et al*. [77], the southern Italian Carbonate Platforms (e.g. the Apulian Platform) must have had an archipelago-like arrangement of small, short-lived, but continuously alternating emerged lands throughout the Late Jurassic to Late Cretaceous. In such a framework, the dispersal of terrestrial faunas was highly limited [77], but for aquatic/semi-aquatic animals feeding on fish and small invertebrates (e.g. molluscs)—that we know were abundant in the area [31,32,34,78,79]—this environment might have offered a favourable refuge, guaranteeing longer survivorship to groups that instead were facing extinction elsewhere. Further corroboration for the hypothesis that *Primitivus manduriensis* is a relict taxon representative of a clade that was declining (or presumed to be extinct in the uppermost Cretaceous) may rely on the collection of additional evidence from these poorly explored and quite promising deposits of the Mediterranean realm.

## Supplementary Material

Additional information and figures

## Supplementary Material

Characters list and additional results of the parsimony analysis

## Supplementary Material

Parsimony data matrix

## Supplementary Material

Bayesian data matrix

## Supplementary Material

Bayesian Inference results

## Data accessibility

Additional data supporting this article can be found as electronic supplementary material (electronic supplementary material 1–5); the dataset for the phylogenetic analyses has been also uploaded to the Dryad Digital Repository (https://doi.org/10.5061/dryad.6t4f27g). The taxon name is registered with a Nomenclature Act on Zoobank (http://zoobank.org/urn:lsid:zoobank.org:act:157568C0-3A9F-4A49-ABAC-458A14CEA9CA).
