# Supplementary material for: A new fossil marine lizard with soft tissues from the Late Cretaceous of southern Italy
Source: R Soc Open Sci. 2018 Jun 20;5(6):172411. doi: 10.1098/rsos.172411 (PMC6030324; doi:10.1098/rsos.172411)
Supplement: Additional information and figures [file rsos172411supp1.pdf]

A new fossil marine lizard with soft tissues from the Late Cretaceous of Southern Italy

Ilaria Paparella[1,2,*], Alessandro Palci[3], Umberto Nicosia[2] & Michael W. Caldwell[1,4]

[1] *Department of Biological Sciences, University of Alberta, Edmonton, AB T6G 2E9, Canada*
[2] *Dipartimento di Scienze della Terra, Sapienza Università di Roma, 00185 Rome, Italy*
[3] *College of Science and Engineering, Flinders University, GPO Box 2100, Adelaide 5001, Australia*
[4] *Department of Earth and Atmospheric Sciences, University of Alberta, Edmonton, AB T6G 2E9, Canada*

Supplementary Material 1

Content

Provenance of the specimen

Supplementary Figures S1-S10

Supplementary Table S1

References

Provenance of the specimen

In 2014, the specimen was presented to one of us (UN), after which it was transferred to the Museum of Paleontology of the University of Rome 'Sapienza' (MPUR). The exact finding place for the specimen was unclear, and the only associated data mentioned a small outcrop of limestones in the town of Nardò (Lecce, Puglia, Southern Italy). During subsequent fieldwork in the area to collect rock samples, and to prospect possible outcrops of interest, we identified the outcrop as a long abandoned quarry that had previously been reported as a site of paleontological excavations: the so called "Cava" locality in Sorbini (1), and Guidotti *et al*. (2). We are currently conducting a project reassessing the geology and stratigraphy of the area based on the macro (e.g., rudists) and nannofossil content in order to refine the age of the strata (upper Campanian – lower Maastrichtian (1, 3, 4)), and further explore these potential *Lagerstätte*-type beds (Cipriani *et al*., work in progress). Uncovering more material from this site represents a unique opportunity to not only improve our knowledge about such poorly sampled deposits, but especially to study the peculiarity of the faunas of the Apulian Platform that could represent the key for understanding the radiation and dispersal of at least non-ophidian pythonomorphs in the Mediterranean Tethys.

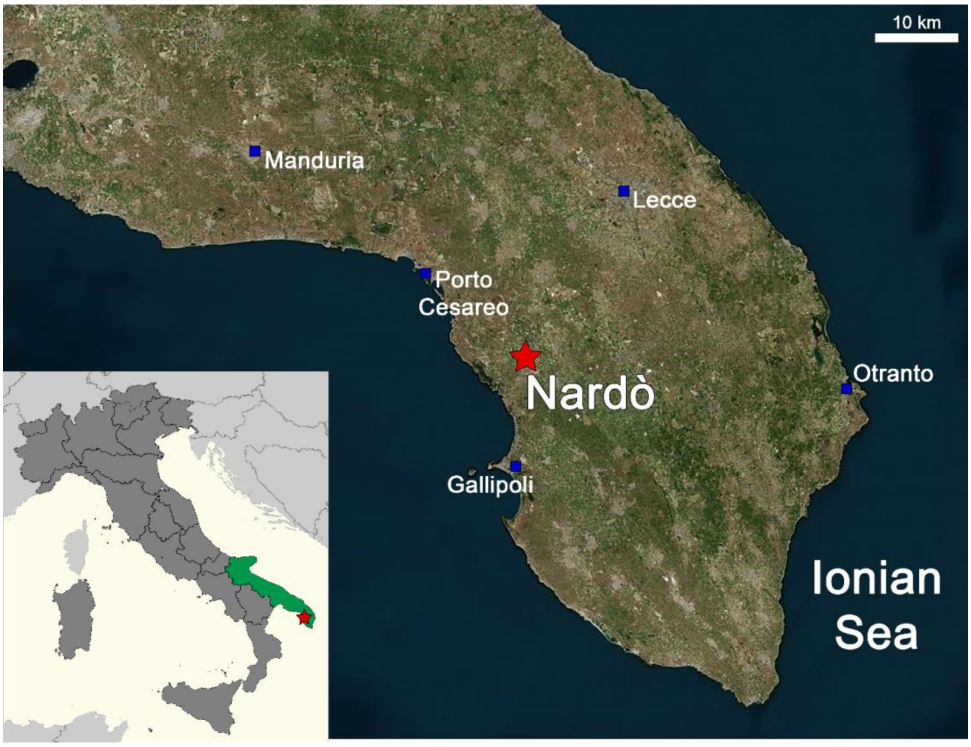

Supplementary Figure S1. Map illustrating the position of the town of Nardò (Lecce, Puglia), finding locality of *Primitivus manduriensis*, MPUR NS 161. The specimen was found in deposits of the upper Campanian – lower Maastrichtian of the Apulian Platform, which makes *Primitivus manduriensis* the latest dolichosaurid up to date. The satellite image was retrieved and modified from Zoom Earth (www.zoom.earth; accessed 10 Nov. 2016), whereas the map of Italy was retrieved and modified from Wikimedia Commons (www.commons.wikimedia.org/wiki/File:Italy_location_map.svg; accessed 9 Nov. 2016).

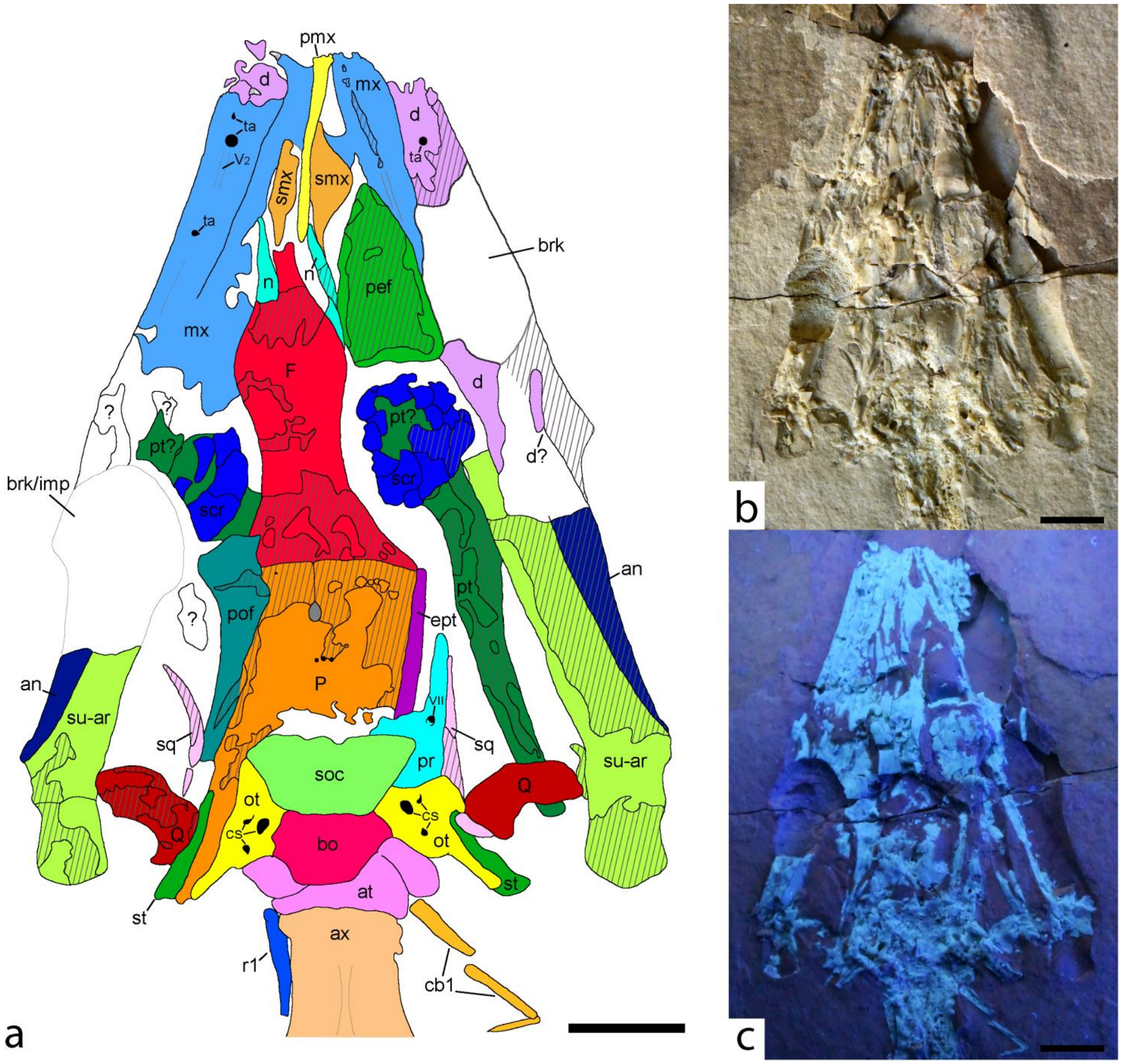

Supplementary Figure S2. Interpretation of the cranial skeleton of *Primitivus manduriensis*, MPUR NS 161. Reconstruction (a) of the cranium of the holotype, realised by combining the observations under natural (b) and UV (c) light. Striped areas indicate where the elements are only preserved as impressions. Scale bars: 1 cm. Abbreviations: an, angular; at, atlas; ax, axis; bo, basioccipital; brk, breakage; cb1, first ceratobranchial; cs, canalis semicircularis; d, dentary; ept, epipterygoid; F, frontal; imp, impression; imp, impression on the matrix; mx, maxillary; n, nasal; ot, otoccipital; P, parietal; pef, prefrontal; pmx, premaxilla; pof, postorbitofrontal; pr, prootic; pt, pterygoid; q, quadrate; r1, first cervical rib; su-ar, surangular-articular; smx, septomaxilla; soc, supraoccipital; sq, squamosal; scr; sclerotic ring ossicles; st, supratemporal; ta, tooth alveolus; V_2, canal for the maxillary branch of the trigeminal nerve; VII, facial nerve foramen; ?, unidentified element.

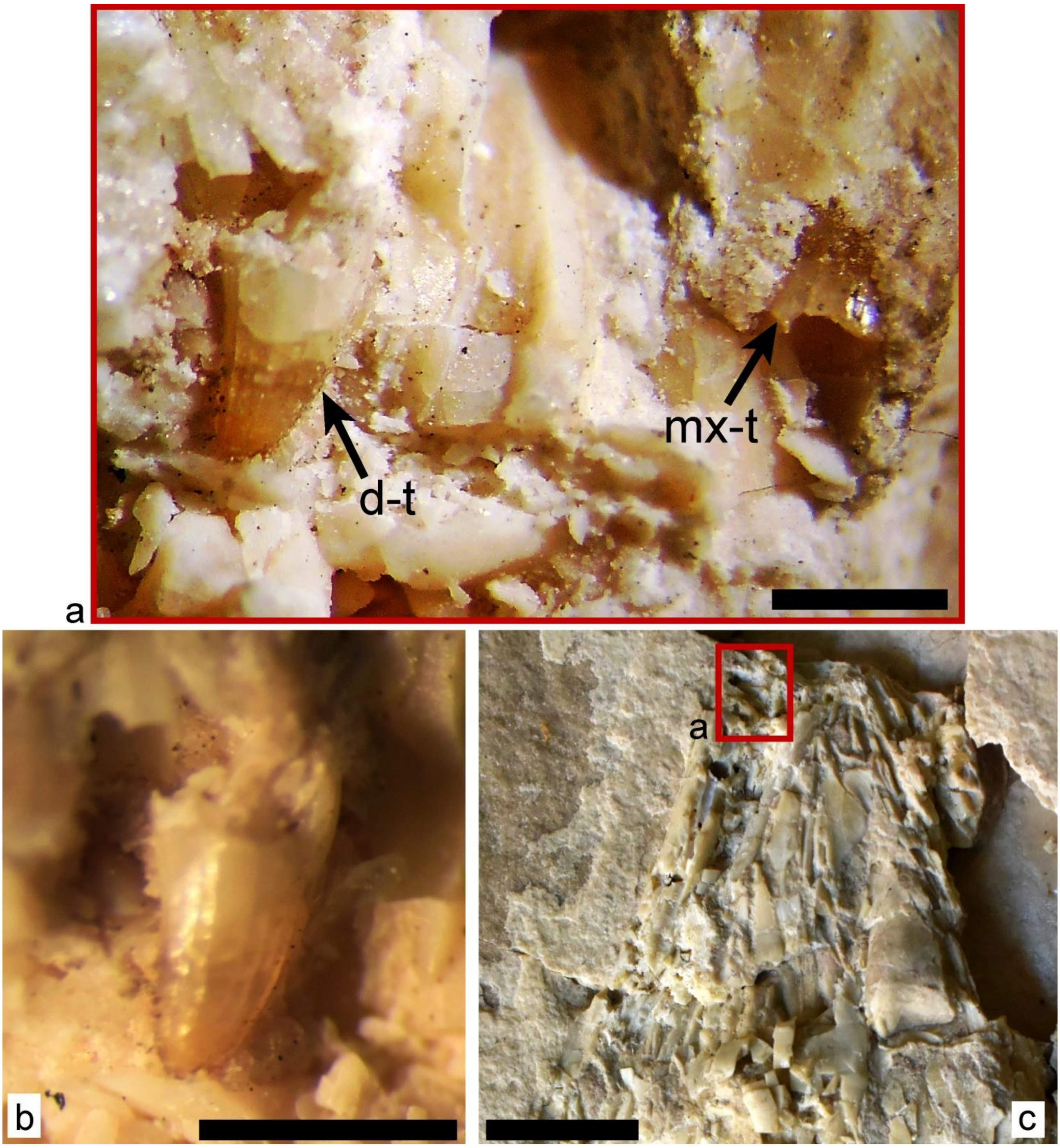

Supplementary Figure S3. Teeth of *Primitivus manduriensis*, MPUR NS 161. Maxillary (a) and dentary (a, b) teeth preserved on the anterior left portion of the skull, pictured under a compound microscope. Both teeth are conical in shape and slightly posteriorly recurved, with multiple longitudinal facets along the tooth crown. Scale bars: a, b, 1 mm; c, 3 cm. Abbreviations: d-t, dentary tooth; mx-t, maxillary tooth.

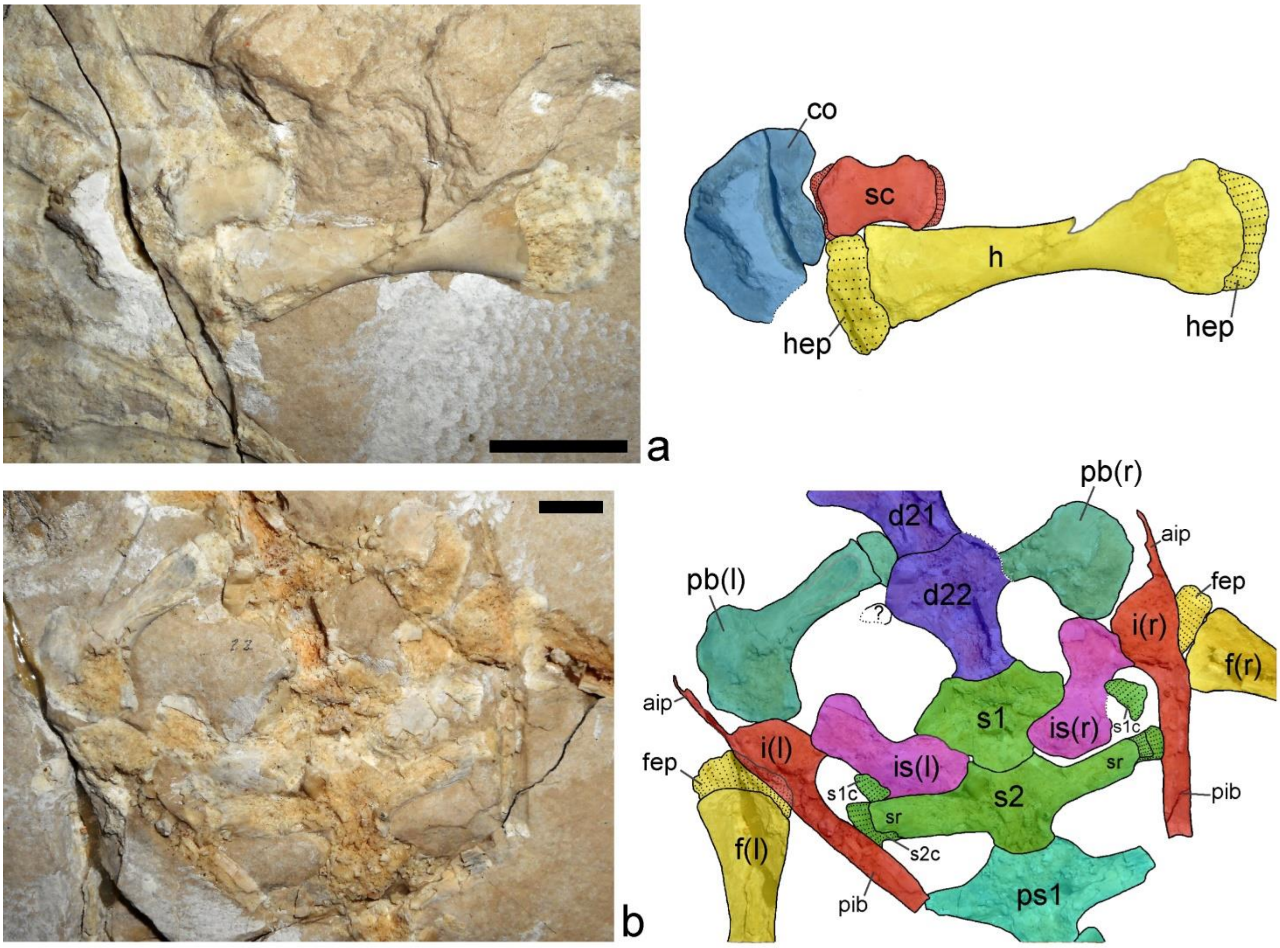

Supplementary Figure S4. Interpretation of *Primitivus manduriensis* pectoral and pelvic regions. Both coracoid and scapula (a), are preserved for the right limb: the elements are reduced and unfused as typical for non-ophidian pythonomorphs. The elements of the pelvic girdle (b) are unfused as well, although the elements must have been tightly articulated together in anatomical position, similarly to 'dolichosaurs' and plesiopelvic mosasauroids. The articulation between the posterior iliac blade and the second sacral rib is quite well preserved, with the contact mediated by cartilage (dotted areas). Scale bars: 1 cm. Abbreviations: aip, anterior preacetabular process of the ilium; co, coracoid; d, dorsal vertebra; f, femur; fep, epiphysis of the femur; h, humerus; hep, epiphysis of the humerus; i, ilium; is, ischium; (l), left; pb, pubis; pib, posterior iliac blade; ps, post-sacral vertebra; (r), right; s, sacral vertebra; s1c, cartilaginous end of the first sacral rib; s2c, cartilaginous end of the second sacral rib; sc, scapula; sr, sacral rib.

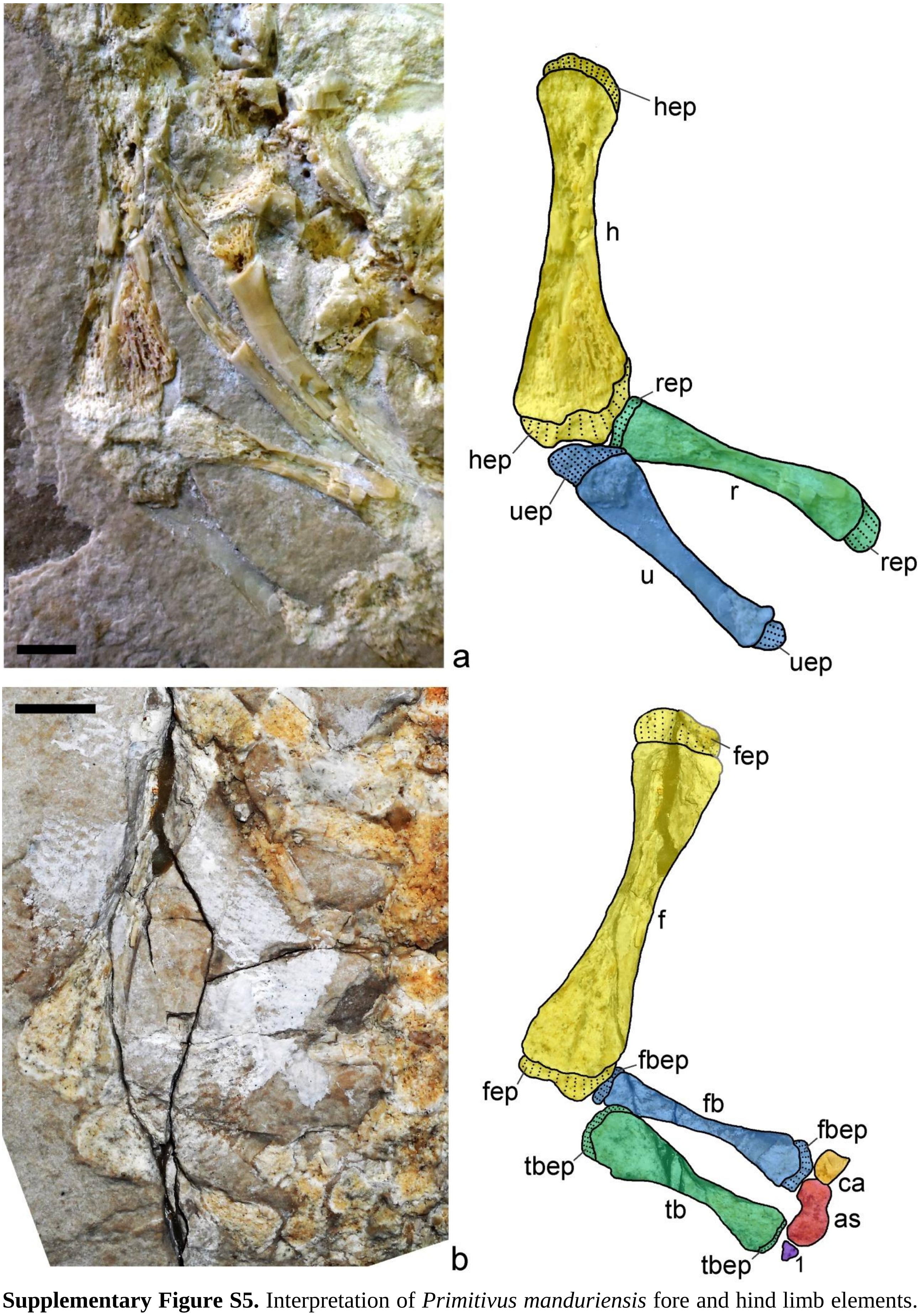

Supplementary Figure S5. Interpretation of *Primitivus manduriensis* fore and hind limb elements. Reconstruction of the left forelimb propodial and epipodials (a), and of the left hindlimb propodial,

epipodials and tarsals (b), emphasizing the presence of incompletely ossified and unfused epiphyses (dotted areas) of the long bones and distally divergent epipodials. Scale bars: 1 cm. Abbreviations: as, astragalus; ca, calcaneum; f, femur; fb, fibula; fep, epiphysis of the femur; fbep, epiphysis of the fibula; h, humerus; hep, epiphysis of the humerus; r, radius; rep, epiphysis of the radius; tb, tibia; tbep, epiphysis of the tibia; u, ulna; uep, epiphysis of the ulna; 1, first distal tarsal.

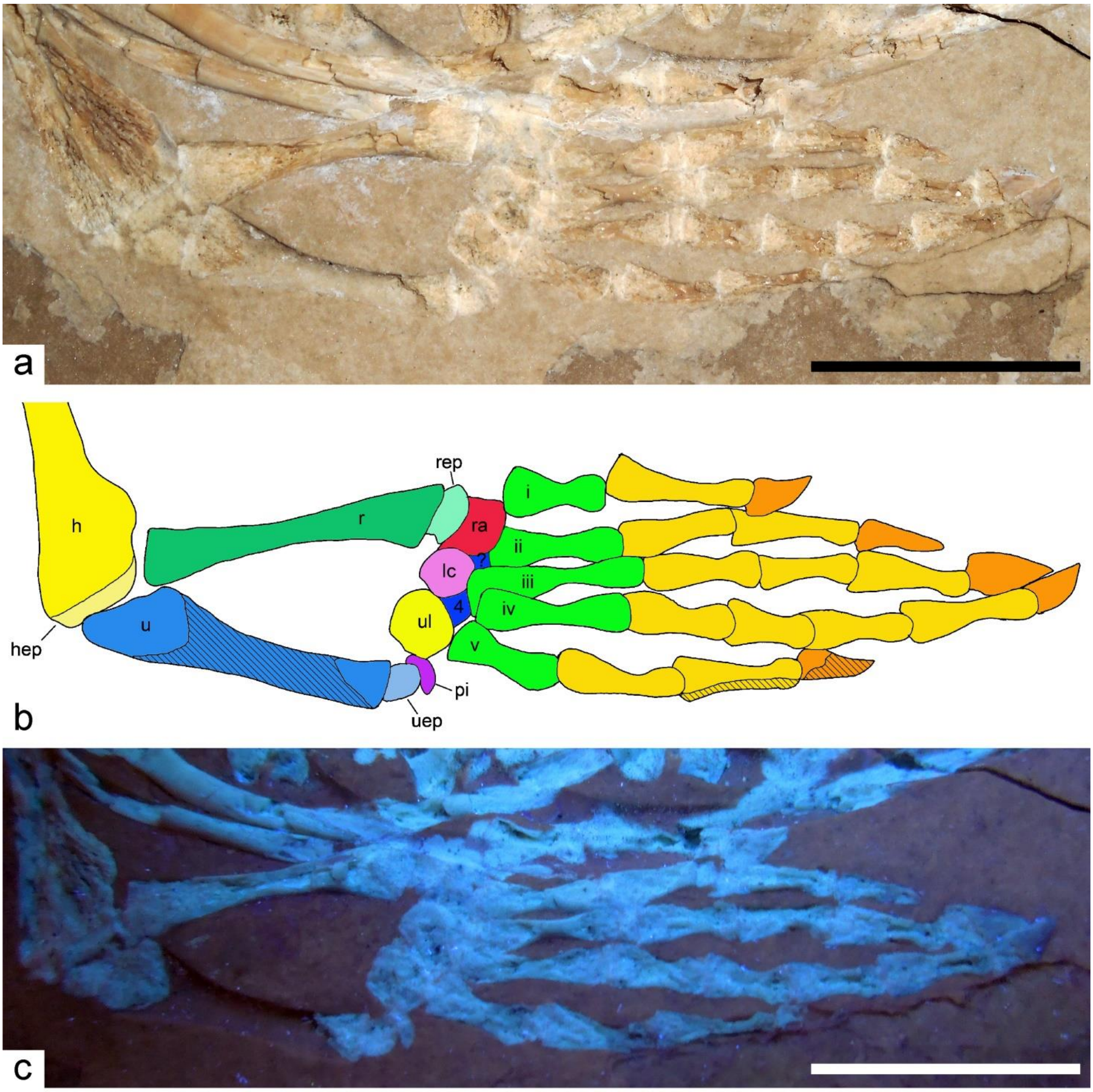

Supplementary Figure S6. Interpretation of the zeugopodial and autopodial elements of *Primitivus manduriensis* forelimb. Reconstruction (b) of the epipodials and manus of the new specimen realised by combining the observations under natural (a) and UV (c) light. Portion preserved only as impressions on the matrix are indicated by stripes. Scale bars: 2 cm. Abbreviations: h, humerus; hep, epiphysis of the humerus; lc, lateral centrale; pi, pisiform; r, radius; ra, radial; rep, epiphysis of the radius; u, ulna; uep, epiphysis of the ulna; ul, ulnar; 4, fourth distal carpal; i-v, first to fifth metacarpals; ?, unidentified distal carpal.

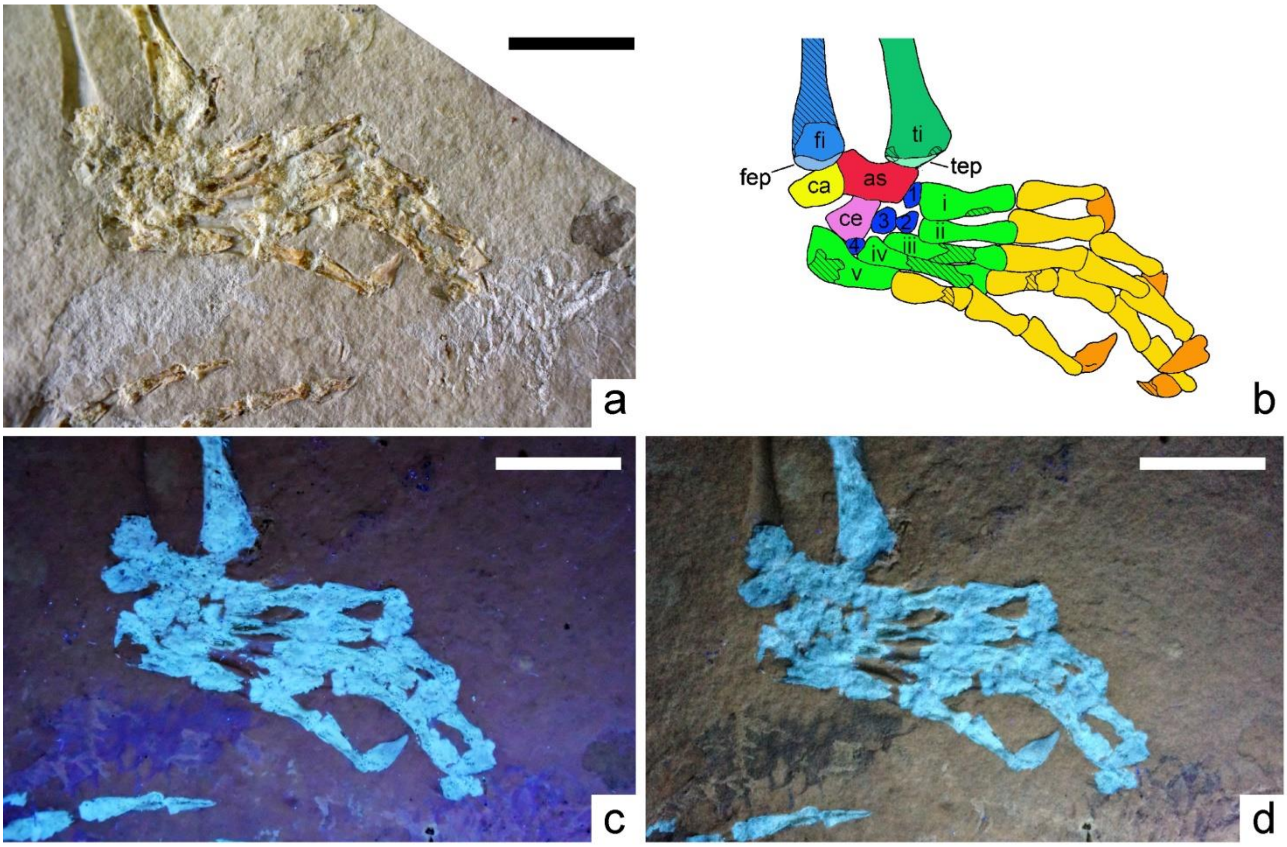

Supplementary Figure S7. Interpretation of the zeugopodial and autopodial elements of *Primitivus manduriensis* hindlimb. Reconstruction (b) of the epipodials and manus of the new specimen realised by combining the observations under natural (a) and UV (c, d) light. The UV lamp used for analysing the specimen can radiate both short (254 nm) and long (365 nm) waves, and the difference is in the resulting spectrum of colours: grey for the short waves (d), and purple of the long waves (c). Striped areas indicate where the bones are only preserved as impressions. Scale bars: 1 cm. Abbreviations: as, astragalus; ca, calcaneum; ce, centrale; fep, epiphysis of the fibula; fi, fibula; tep, epiphysis of the tibia; ti, tibia; 1-4, first to fourth distal tarsals; i-v, first to fifth metatarsals.

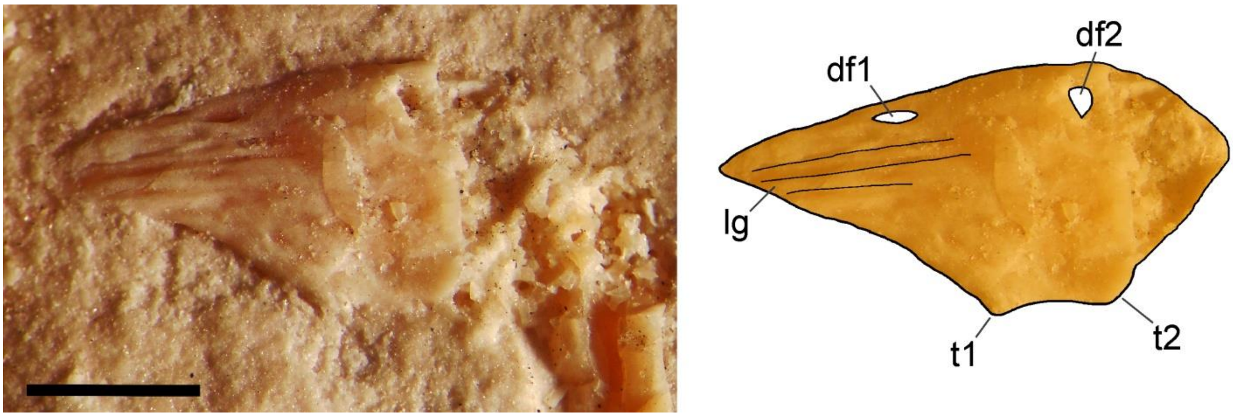

Supplementary Figure S8. Interpretation of the ungual phalanx of *Primitivus manduriensis* pes. Illustration of the anatomical details preserved for one of the claw-like ungual phalanges of MPUR NS 161. Scale bar: 2 mm. Abbreviations: df, dorsal foramen; lg, longitudinal grooves; t, tubercle.

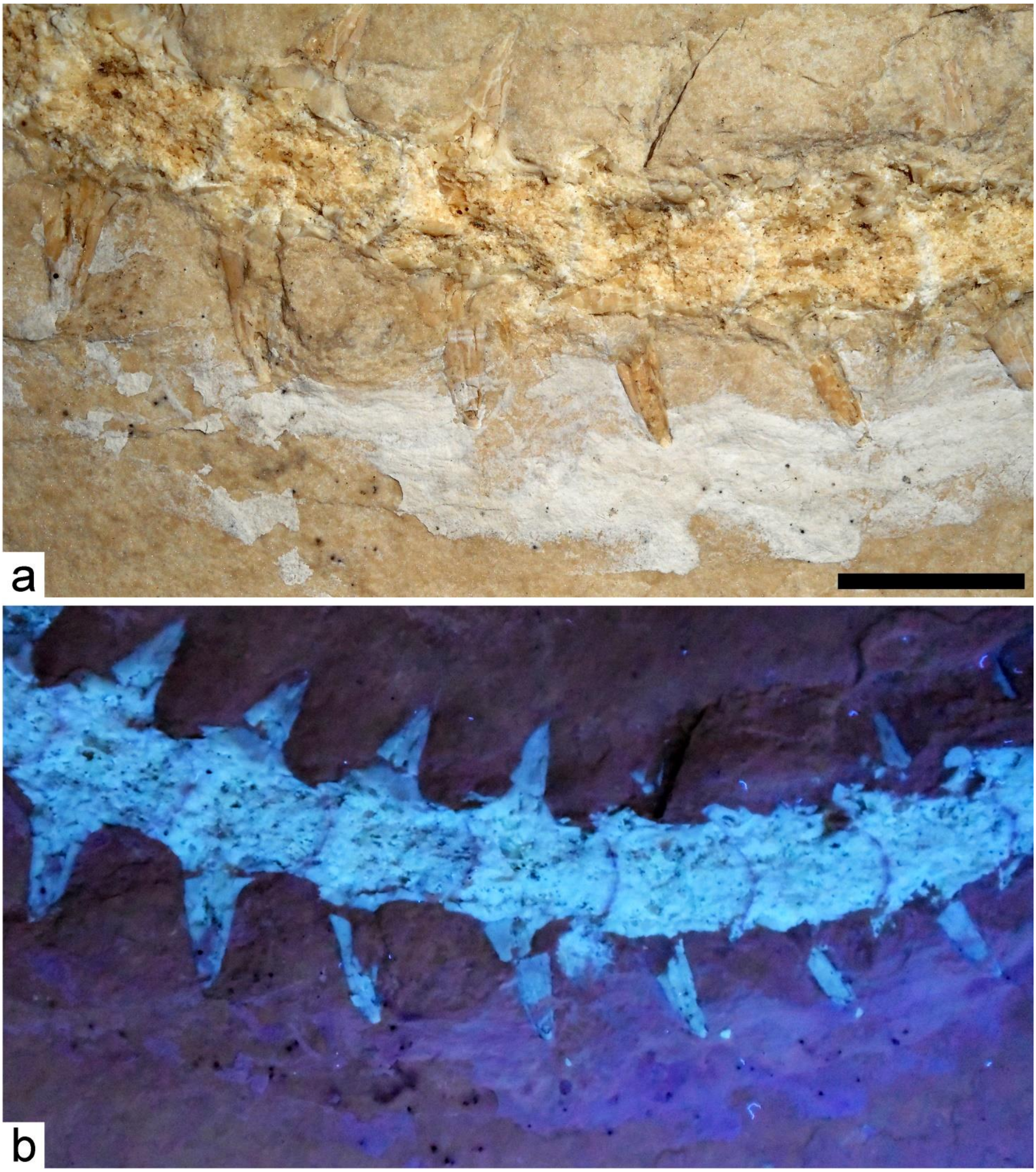

Supplementary Figure S9. *Primitivus manduriensis* MPUR NS 161 imaging of anterior portion of the tail at natural (a) and UV (b) light. Anterior portion of the tail is exposed in dorsal view, and an extended portion of muscle bundles (dark purple in b) is visible on the left side of the column. According to the position and difference in fibers and bundles orientation and size, we recognize these muscles as part of the *musculus transversospinalis* and *musculus iliocaudalis* (see also Figure 8d-g). Scale bar: 1 cm.

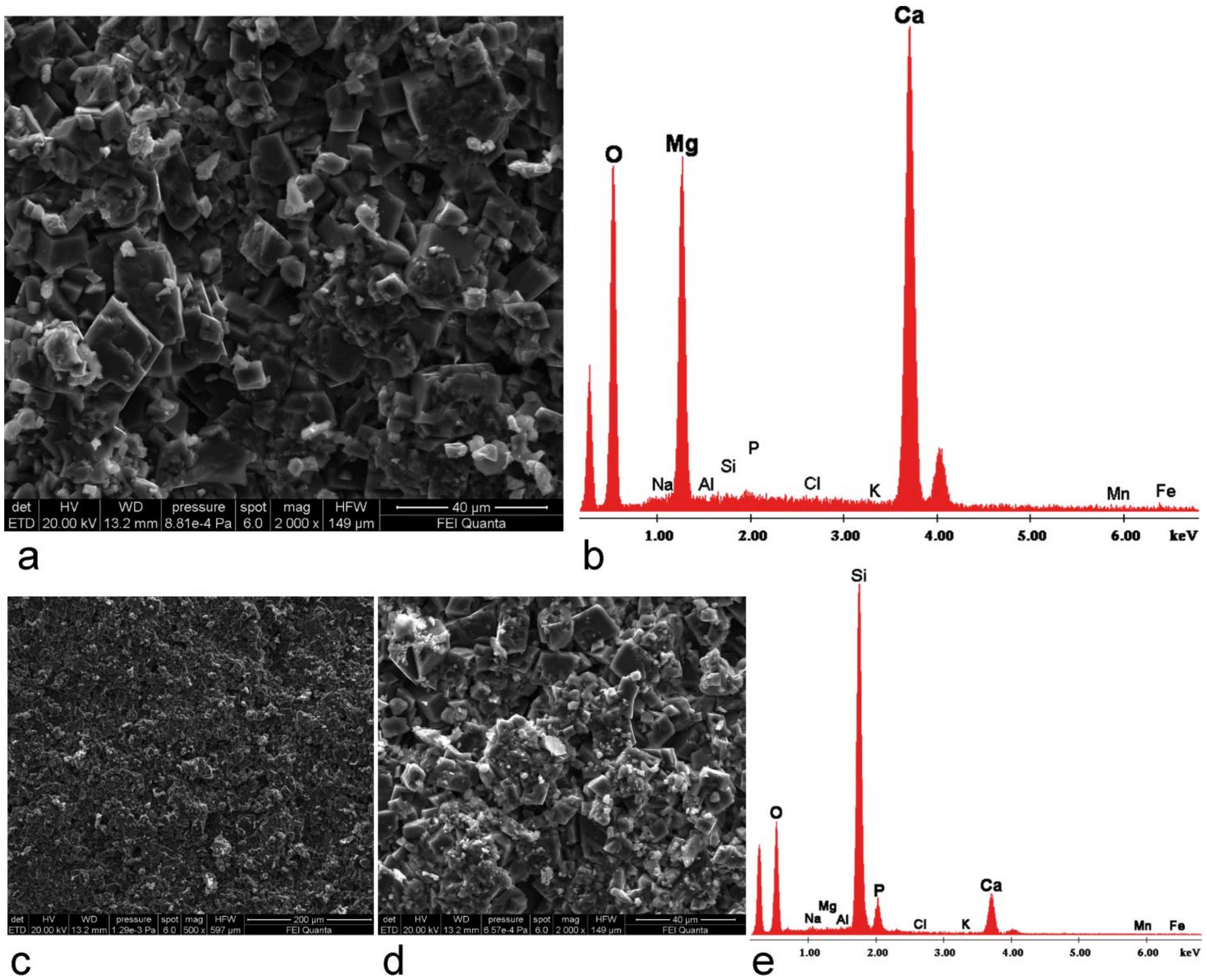

Supplementary Figure S10. Results of the EDX analyses for sediment (a-b) and gut contents (c-e). As shown in Figure 9 for the bony and soft tissues, the composition for the material preserved in the posterior trunk region consists of replacement calcium phosphate (rich in Ca and P), while the sediment is rich in Ca and Mg, but there is no significant content of P. The high peak of silicon (Si) in the gut content spectrum must be related to the substrate bearing the sample which is made of silica-glass.

Supplementary Table S1. Measurements and counts for *Primitivus manduriensis*, MPUR NS 161. Values reported for each vertebral series are average measurements. Abbreviations: d, diameter; dist, distal; H, height; L, length; max, maximum; min, minimum; mc, metacarpal; mt, metatarsal; n., number of; prox, proximal; seg., segment; W, width.

**Element**	**Measurement (mm) or count**	**Element**	**Measurement (mm) or count**
skull L	~70	ulna W(prox)	5.76
skull W	42.51	ulna W(mid-shaft)	2.67
mandible L	62	ulna W(dist)	4.23
postorbital L	25	mc–I L	9.59
orbit d(antero-posterior)	9	mc–I W	2.71
orbit d(transversal)	6.5	mc–II L	9.39
parietal foramen d(antero-posterior)	3.6	mc–II W	2.5
parietal foramen d(transversal)	1.9	mc–III L	9.78
n. cervicals	9-10	mc–III W	2.5
n. dorsals	22	mc–IV L	10.12
n. sacrals	2	mc–IV W	2.5
n. caudals (postsacral vertebrae)	>40	mc–V L	8.34
cervical centrum L	13.29	mc–V W	2.5
cervical centrum W	11.05	manus phalangeal formula	2-3-4-5-3
dorsal centrum L	15.09	hindlimb L	138
dorsal centrum W	11.92	hindlimb W(prox)	13.01
sacral centrum L	17.3	hindlimb W(dist)	17.42
sacral centrum W	19.08	femur L	43.23
anterior caudal centrum L	10.38	femur W(prox)	13.01
anterior caudal centrum W	12.48	femur W(mid-shaft)	5.41
mid-caudal centrum L	8.45	femur W(dist)	9.72
mid-caudal centrum H	5.67	tibia L	28.1
mid-caudal centrum W	9.14	tibia W(prox)	8.04
mid-caudal neural spine H	10.85	tibia W(mid-shaft)	3.27
mid-caudal neural spine W	2.93	tibia W(dist)	6.93
longest trunk rib L	81.5	fibula L	23.91
trunk rib W (head)	4.02	fibula W(prox)	4.21
tail H	38	fibula W(mid-shaft)	1.98
precaudal L	571.85	fibula W(dist)	5.59
forelimb L	107.5	mt–I L	9.35
forelimb W(prox)	8.72	mt–I W	2.11
forelimb W(dist)	16.5	mt–II L	10.53
humerus L	34.74	mt–II W	1.46

humerus W(prox)	8.72	mt–III L	12.19
humerus W(mid-shaft)	4.62	mt–III W	1.69
humerus W(dist)	11.5	mt–IV L	10.94
radius L	22.83	mt–IV W	2
radius W(prox)	5.34	mt–V L	10.37
radius W(mid-shaft)	2.25	mt–V W	2.96
radius W(dist)	5.48	pes phalangeal formula	2-3-4-5-4
ulna L	21.13		

References

1. Sorbini L. The Cretaceous fishes of Nardò. I. Order Gasterosteiformes (Pisces). Bollettino del Museo Civico di Storia Naturale di Verona. 1981;VIII:1-27.
2. Guidotti G, Landini W, Sorbini L, Varola A, editors. Le ittiofaune del Cretaceo di Alessano e Nardò. Guida alle escursioni. XII Convegno della Società Paleontologica Italiana; 1993 28 Settembre - 2 Ottobre, 1993; Terra d’Otranto, Lecce, Puglia, Italy.
3. Medizza F, Sorbini L. Il giacimento del Salento (Lecce). I vertebrati fossili italiani. Verona1980. p. 131-4.
4. Sorbini L. New fish bed localities of latest Campanian age. 1978;V:607-8.
