# Supplementary material for: A new fossil marine lizard with soft tissues from the Late Cretaceous of southern Italy
Source: R Soc Open Sci. 2018 Jun 20;5(6):172411. doi: 10.1098/rsos.172411 (PMC6030324; doi:10.1098/rsos.172411)
Supplement: Characters list and additional results of the parsimony analysis [file rsos172411supp2.pdf]

# A new fossil marine lizard with soft tissues from the Late Cretaceous of Southern Italy

Ilaria Paparella[1,2,*], Alessandro Palci[3], Umberto Nicosia[2] & Michael W. Caldwell[1,4]

*[1] Department of Biological Sciences, University of Alberta, Edmonton, AB T6G 2E9, Canada*
*[2] Dipartimento di Scienze della Terra, Sapienza Università di Roma, 00185 Rome, Italy*
*[3] College of Science and Engineering, Flinders University, GPO Box 2100, Adelaide 5001, Australia*
*[4] Department of Earth and Atmospheric Sciences, University of Alberta, Edmonton, AB T6G 2E9, Canada*

## Supplementary Material 2

*Content*

Characters list

MP optimal trees

IWMP optimal tree

List of apomorphies from the MP analysis

References

## Characters list

Description of characters used to assess the phylogenetic relationships of the new taxon in the parsimony (MP and IWMP) analyses. All scorings are based on personal observation on relevant fossil and extant specimens, the literature, or images available on the Digimorph online database (www.digimorph.org). Most of the characters are taken or modified from Palci & Caldwell[1]; some of the characters are new, as indicated in the remarks.

**Institutional Abbreviations** – **HUJ-Pal**, Hebrew University of Jerusalem, Palaeontology Collections, Jerusalem, Israel; **MACN**, Museo Argentino de Ciencias Naturales "Bernardino Rivadavia," Buenos Aires, Argentina; **MLP**, Museo de La Plata, La Plata, Argentina; **NHML**, Natural History Museum, London, England; **QM**, Queensland Museum, Brisbane, Queensland, Australia.

1. Premaxilla: does not contact frontals (0) / contacts frontals (1).
2. Premaxilla with median palatal ramus bearing foramina: absent (0) / present (1).
3. Premaxillary lateral foramina: absent (0) / present (1). *Yurlunggur* was rescored from state 1 to state 0[2]; *Pontosaurus kornhuberi* was rescored from unknown (?) to state 0[3].
4. Premaxilla-maxilla contact: immobile and sutural (0) / mobile and non-sutural (1).
5. Posterior process of maxilla: long, reaching or extending past middle of ventral margin of orbit (0) / short, not reaching middle of ventral margin of orbit (1).
6. Lacrimal: present, either permanently separate or fusing with prefrontal during ontogeny (0) / absent, never present as a discrete element (1).
7. Lacrimal foramen: single opening (0) / double opening (1).
8. Jugal: does not extend anteriorly past orbit (0) / extends anteriorly past orbit (1).
9. Jugal: with large posterior process (0) / without large posterior process (1). *Xenosaurus* and *Shinisaurus* were rescored from state 1 to state 0[4, 5]; *Lanthanotus* and *Varanus* were rescored from state 0 to state 1[4]; *Eupodophis* was rescored from inapplicable (-) to unknown (?); *Pachyrhachis* was rescored from state 0 to unknown (?). *Eupodophis* and *Pachyrhachis* do have a jugal, but it is not clear whether the posteroventral corner of the bone represents a posterior process homologous to that of lizards (point of attachment of quadratojugal ligament) or simply the posterior end of the expanded articular surface beteween jugal and maxilla.
10. Jugal: lacking dermal sculpture (0) / with dermal sculpture (1).
11. Nasals: large (0) / greatly reduced or absent (1).
12. Nasals: paired elements (0) / single median element (1).
13. External naris: not retracted, prefrontal and frontal both excluded from posterior narial margin by nasal and maxilla (0) / slightly retracted, prefrontal (but not frontal) enters posterior narial margin (1) / greatly retracted, prefrontal and frontal enter posterior narial margin (2).
14. Frontals: single median element (0) / paired elements (1).
15. Frontal: enters orbital margin, prefrontal does not contact postfrontal or postorbital (0) / excluded from orbital margin, prefrontal contacts postfrontal or postorbital (1). *Aigialosaurus* has been rescored from state 0&1 to state 0[6, 7]; *Pachyrhachis* has been rescored from state 1 to unknown (?). The skull reconstruction of *Pachyrhachis* in Caldwell [8] suggests that the frontal was excluded from the orbital margin (state 0), however, due to poor preservation,

disarticulation, and crushing of the only known skull of *Pachyrhachis* this character is best left as unknown (?).

16. Frontoparietal suture: in dorsal view, simple straight transverse contact (0) / in dorsal view, complex curved or interdigitating contact (1).
17. Postfrontal (or dorsomedial portion of single posterior orbital bone): forked medially, with an anterior process along the frontal and a posterior process along the parietal (0) / not forked medially, does not extend a long distance along frontal or parietal (1). *Heloderma* was rescored from state 1 to state 0 [4]; *Haasiophis* was rescored from state 1 to state unknown (?) (a postfrontal cannot be clearly identified in *Haasiophis*, the homology of the element preserved dorsal to the orbit in the type and only specimen is dubious [8]).
18. Palpebral (superciliary) ossifications on dorsal margin of orbit: present (0) / absent (1). *Aigialosaurus* was rescored from unknown (?) to state 1. No palpebral is known in *Aigialosaurus* [6, 7].
19. Postorbital: present (0) / absent (1). *Dinilysia, Eupodophis, Haasiophis, Pachyrhachis* and *Yurlunggur* were rescored from state 0 to state 1 [9].
20. Postorbital ventral process: small, forming less than half of posterior orbital margin, postorbital primarily a temporal bone (0) / prominent, forming half or more of posterior orbital margin, postorbital primarily an orbital bone (1). *Dinilysia, Eupodophis, Haasiophis* and *Pachyrhachis* were rescored from state 1 to inapplicable (-). *Yurlunggur* was rescored from state 0 to inapplicable (-). All these snakes lack a postorbital.
21. Pineal foramen: present (0) / absent (1).
22. Parietal table and jaw adductor muscles: parietal table very wide, jaw adductors restricted entirely to ventral surface of parietal (0) / parietal table much narrower posteriorly, jaw adductors extend onto lateral margin of parietal (1) / parietal table tapers posteriorly into a sagittal crest (2). Modified from Palci & Caldwell[1].
23. Suspensorial ramus (posterolateral process) of parietal: well developed (0) / extremely short or absent (1).
24. Upper temporal arch: complete, upper and lower temporal fenestrae separated (0) / incomplete, upper and lowertemporal fenestra confluent (1). *Haasiophis* and *Yurlunggur* were rescored from inapplicable (-) to state 1 [2, 8].
25. Temporal arch: without canthal crest (0) / with canthal crest (1).
26. Dorsal process of squamosal: absent (0) / present (1). *Pachyrhachis* was rescored from state 0 to unknown (?). *Pachyrhachis* lacks a squamosal, what has been tentatively identified as a possible squamosal e.g., [10] may also be the shaft of the stapes or part of the hyoid apparatus.
27. Supratemporal: in deep position, on ventrolateral surface of parietal (0) / in superficial position, on dorsolateral surface of parietal (1). *Yurlunggur* was rescored from unknown (?) to state 1 (AP pers. obs. on QMF45391); *Adriosaurus* was rescored from state 1 to unknown (?) (AP pers. obs. on NHML R2867); *Pontosaurus lesinensis* was rescored from state 1 to state 0 [11].
28. Supratemporal: confined to skull roof (0) / forms part of paroccipital process and/or braincase (1).
29. Supratemporal: present (0) / absent (1).
30. Supratemporal-prootic contact: absent (0) / present (1). *Haasiophis* was rescored from state 0 to state 1 (AP pers. obs. on HUJ-Pal. EJ 695); *Yurlunggur* was rescored from unknown (?) to state 1 (AP pers. obs. on QMF45391).

31. Quadrate suspension: mobile, articulates dorsally with squamosal, supratemporal and opisthotic (0) / mobile, articulates dorsally with supratemporal, little or no contribution from other elements (1) / mobile, articulates dorsally with opisthotic, little or no contribution from other elements (2). *Pontosaurus kornhuberi* was rescored from state 0 to unknown (?); *Pontosaurus lesinensis* was rescored from unknown (?) to state 0 [3, 11].
32. Quadrate: tympanic crest (outer conch) directed laterally and a well-developed wall (0) / tympanic crest directed laterally but a low ridge (1) / distinct tympanic crest absent and external surface of quadrate only weakly concave (2).
33. Quadrate suprastapedial process: directed mostly posteriorly (0) / recurved posteroventrally (1) / absent (2). As examples, *Varanus bengalensis* represents state 0, while *Mosasaurus hoffmannii* represents state 1. Modified from Palci & Caldwell[1].
34. Mandibular articulation of quadrate: saddle-shaped, with lateral and medial condyles (0) / flat, a single continuous condyle (1). *Aigialosaurus* was rescored from state 1 to unknown (?), because the articulation is not visible in either *A. bucchichi* or *A. dalmaticus* [6, 7]; *Dinilysia* was rescored from unknown (?) to state 0 (AP pers. obs. on MACN-RN 1013); *Pontosaurus lesinensis* was rescored from state 1 to unknown (?) because the articulation is not visible [11].
35. Ventromedial processes of frontals: not contacting anything below olfactory tracts (0); abutting or sutured with each other below olfactory tracts (1); contacting each other and parabasisphenoid below olfactory tracts (2). *Dinilysia* was rescored from states 0&1 to state 2 [12]. Modified from Palci & Caldwell[1].
36. Parietal downgrowths: absent or weakly developed ridges (0) / prominent flanges (1). *Pontosaurus lesinensis* was rescored from state 0 to unknown (?). This feature is not clear in the type and only specimen [11].
37. Parietal downgrowths: not sutured to prootic (0) / sutured to prootic (1).
38. Parietal downgrowths: not contacting parabasisphenoid or orbitosphenoid (0) / contacting parabasisphenoid (1). *Haasiophis* was rescored from unknown (?) to state 1 (AP pers. obs. on HUJ-Pal. EJ 695).
39. Optic foramina: not enclosed in bone (0) / enclosed partly or entirely by frontals (1). *Pachyrhachis* was rescored from state 1 to unknown (?) because the optic foramen is not exposed in *Pachyrhachys* [8].
40. Trigeminal foramen or foramina: anterior margin not enclosed in bone (0) / anterior margin enclosed by descending flange of parietal and/or prootic (1). *Pachyrhachis* was rescored from state 1 to unknown (?) (the trigeminal foramen is not exposed in *Pachyrhachys* [8]).
41. Crista prootica (ridge on lateral surface of the prootic, overhanging foramen pro nervi facialis): well-developed lateral flange (0) / reduced to weak ridge, or absent (1). *Yurlunggur* was rescored from state 0 to state 1 (AP pers. obs. on QMF45111).
42. Foramen pro nervi facialis: single (0) / double (1) /exit confluent with trigeminal opening (2). *Yurlunggur* was rescored from unknown (?) to state 0 (AP pers. obs. on QMF45111). Modified from Palci & Caldwell[1].
43. Basipterygoid process: long, i.e., projecting far anterolaterally beyond the body of the basisphenoid (0) / short, i.e., not projecting very far beyond the body of the basisphenoid (1). *Aigialosaurus*, *Pontosaurus lesinensis* and *Pachyrhachis* were rescored from state 1 to unknown (?) because the processes are not visible in any of the available specimens for these taxa [6, 7, 8, 11]; *Haasiophis* was rescored from unknown to state 1 (AP pers. obs. on HUJ-Pal. EJ 695).

44. Basal tubera: posteriorly located, very near to occipital condyle (0) / anteriorly located, well away from occipital condyle (1). *Yurlunggur* was rescored from state 1 to unknown (?). *Yurlunggur* lacks proper basal tubera, and has instead an elongate crest directed posterolaterally.
45. Posterior opening of vidian canal: at basisphenoidprootic suture (0) / situated within basisphenoid (1).
46. Posterior opening of vidian canal: situated anteriorly, well in front of the posterior end of the basisphenoid (0) / situated posteriorly, near the posterior end of the basisphenoid (1).
47. Opisthotic sub-horizontal flange posterior to basal tubera: weak or absent, with most of the stapes exposed in ventral view (0) / wide, extending posterolaterally from basal tubera, and obscuring much of the stapes in ventral view (1). *Eupodophis* was rescored from state 0 to unknown (?). All known specimens of *Eupodophis* are too porly preserved to score for this character [13, 14]. Modified from Palci & Caldwell[1].
48. Supraoccipital: does not contact parietal, unossified gap persists between the two elements (0) / abuts parietal, the two elements meet but contact is non-sutural, and a tiny gap might remain between the two elements along part of the dorsal edge of the supraoccipital (1) / sutural contact with parietal, entire anterodorsal edge of supraoccipital contacts parietal (2).
49. Supraoccipital: situated ventral or posteroventral to parietal, does not form part of posterior skull roof (0) / situated posterior to parietal, forms part of posterior skull roof (1).
50. Postemporal fenestra: present as an opening (0) / completely closed via sutural contact of the skull roof and otic region of braincase (1). *Aigialosaurus* was rescored from state 0 to unknown (?) because the posterior region of the skull is not visible in either *A. bucchichi* or *A. dalmaticus* [6, 7]; *Haasiophis* was rescored from unknown (?) to state 1 because the position of the parietal on the skull roof and the lack of paroccipital processes in *Haasiophis* exclude the possibility that a postemporal fenestra was present [15].
51. Septomaxilla-maxilla contact: rigid, septomaxilla extensively sutured to the dorsal surface of the palatal flange of the maxilla (0) / septomaxilla not sutured to maxilla (1). *Dolichosaurus* and *Pachyrhachis* were rescored from 1 to unknown (?). The septomaxilla is unknown in both taxa [8, 16].
52. Median flange of septomaxilla: short, not reaching level of prefrontal (0) / long, extends posteriorly to reach level of prefrontal (1). *Yurlunggur* was rescored from state 1 to state 0 (AP pers. obs. on QMF45391).
53. Opening of Jacobson's organ: enclosed fully by maxilla and vomer, sometimes with a tiny contribution from the septomaxilla, not confluent with choana (0) / enclosed partly by maxilla and vomer, confluent posteriorly with choana (1) / enclosed fully by vomer and septomaxilla only, not confluent with choana (2). *Pachyrhachis* was rescored from state 2 to unknown (?) as the vomeronasal opening is not visible in *Pachyrhachys* [8]; *Yurlunggur* was rescored from state 0 to state 2 (AP pers. obs. on QMF45391).
54. Palatine-vomer contact: immobile, sutural contact (0) / mobile, non-sutural contact (1). *Dinilysia* was rescored from unknown (?) to state 0 [12]; *Eupodophis* was rescored from unknown to state 1 as a long tapering choanal process of the palatine of *Eupodophis* suggests that this element must have had a loose mobile connection with the vomer [14]; *Pachyrhachis* was rescored from state 1 to unknown (?) because the vomer is not visible in *Pachyrhachis* [8].
55. Palatine anterior dentigerous process: absent (0) / present (1).

56. Ectopterygoid: does not enter cheek (0) / enters cheek as a sliver sandwiched between maxilla and jugal (1). *Pachyrhachis* was rescored from state 0 to unknown (?). Because of disarticulation and compression it is not clear whether the ectopterygoid of *Pachyrhachys* would have entered the cheek region or not [8].
57. Ectopterygoid-palatine contact: absent, maxilla enters suborbital fenestra (0) / present, maxilla excluded from suborbital fenestra (1). *Pachyrhachis* was rescored from state 0 to unknown (?). The contact between ectopterygoid and palatine is not visible in this taxon [8].
58. Interpterygoid vacuity (‘‘pyriform recess’’ of Estes et al.[17]): open and wide (0) / open and narrow (1).
59. Pterygoid: anterior (palatine) process merges gradually, in a gentle curve, with the lateral (ectopterygoid) process (0) / anterior process distinctly set off from lateral process, the two portions meeting at a distinct ‘corner’ (1). *Pontosaurus lesinensis* was rescored from state 0 to unknown (?). The region of interest is not exposed in the skull of *P. lesinensis* [11].
60. Mandibular symphysis: rigid anterior tips of dentary with a distinct flat symphysial area (0) / mobile anterior tips of dentary smoothly rounded and without distinct symphysial area (1). *Yurlunggur* was rescored from unknown (?) to state 1 (AP pers. obs. on QMF45391).
61. Mental foramina on lateral surface of dentary: three or more foramina (0) / two or fewer foramina (1). *Pachyrhachis* was rescored from state 1 to state 0 [9]; *Pontosaurus lesinensis* was rescored from state 1 to unknown (?) because no mental foramina are visible in the type and only specimen [11].
62. Dentary: curved in lateral view, with concave dorsal (alveolar) edge (0) / straight in lateral view, with straight dorsal edge (1). *Haasiophis* was rescored from state 0 to state 1 because its dentary is curved medially, but in lateral view its dorsal margin would appear quite straight [15].
63. Dentary: with small posterodorsal extension onto anterolateral part of coronoid process (0) / does not cover lateral surface of coronoid process (1). *Dolichosaurus* was rescored from state 1 to unknown (?): the only dentary known for *Dolichosaurus longicollis* is too fragmentary to be scored for this character [16].
64. Anterior (symphysial) end of Meckel’s canal: extends along ventral margin of lower jaw (0) / confined to medial surface of lower jaw (1). *Dolichosaurus* was rescored from state 1 to unknown (?): the only dentary known for *Dolichosaurus longicollis* is too fragmentary to be scored for this character [16].
65. Subdental shelf: large (0) / weakly developed (1) / absent (2). Modified from Palci & Caldwell (2010). For example the shelf is large in *Platecarpus tympaniticus* (state 0), weakly developed in *Shinisaurus crocodilurus* (state 1) and absent in *Anilius scytale* (state 2). *Dinilysia* and *Yurlunggur* were rescored from state 2 to state 0 [2, 12]; *Haasiophis* was rescored from unknown (?) to state 2 (AP pers. obs. on HUJ-Pal. EJ 695); *Pachyrhachis* was rescored from state 2 to unknown (?) because the medial aspect of the dentary is partially exposed only in the paratype, HUJ-Pal 3775, but the condition of the subdental shelf is unclear [8]; *Dolichosaurus* was rescored from state 1 to unknown (?) as the only dentary known for *Dolichosaurus longicollis* is too fragmentary to be scored for this character [16].
66. Posterior margin of lateral surface of dentary: no notch present (0); shallow notch present (1); deep notch present (2). For example, the notch is absent in *Platecarpus tympaniticus* (state 0), shallow in *Shinisaurus crocodilurus* (state 1), and deep in *Python molurus* (state 2). *Yurlunggur* was rescored from state 0 to state 2 (AP pers. obs. on QMF45391); *Dolichosaurus* was rescored from state 1 to unknown (?) as the only dentary known for *Dolichosaurus*

*longicollis* is too fragmentary to be scored for this character [16]. Modified from Palci & Caldwell[1].

67. Dentary-postdentary articulation: extensive overlap (0) / reduced overlap (1). *Yurlunggur* was rescored from state 2 to state 1 (although a postdentary is not known in *Yurlunggur*, the articular surface on the dentary indicates that the connection between these two bones was fairly loose and overlap was reduced; AP pers. obs. on QMF45391).
68. Splenial: large, extending anteriorly past middle of tooth row (0) / small, only reaching middle of tooth row (1).
69. Splenial: extends posteriorly onto postdentary bones, past apex of coronoid process (0) / extends posteriorly onto postdentary bones but does not reach level of apex of coronoid process (1) / does not substantially overlap postdentary elements (2).
70. Anterior tip of splenial: on ventral edge of dentary (0) / on medial surface of dentary (1). *Dinilysia* was rescored from state 0 to unknown (?) (due to proximity of the anterior tip of the splenial to the ventral edge of the dentary and uncertainty in the original orientation of the dentary this character cannot be confidently assessed); *Eupodophis* was rescored from unkown (?) to state 1 [14]; *Haasiophis* was rescored from state 0 to state 1 [15].
71. Splenial-dentary contact: extensive bony contact (0) / reduced bony contact, much intervening connective tissue (1).
72. Splenial-angular contact: in medial view, overlapping, irregular, and with limited mobility (0) / inmedial view, abutting, straight (vertical), and highly mobile (1). *Lanthanotus* was rescored from state 0 to state 1 [18].
73. Splenial-angular contact: not, or very slightly, exposed in lateral view (0) / greatly exposed in lateral view (1).
74. Anteromedial process of coronoid: long, extensive overlap on medial surface of dentary in front of coronoid process (0) / short, coronoid does not greatly overlap medial surface of dentary in front of coronoid process (1). *Pontosaurus lesinensis* was rescored from state 1 to state 0 [11].
75. Anterolateral process of coronoid: present, overlapping lateral surface of dentary (0) / absent, coronoid does not overlap lateral surface of dentary (1). *Dolichosaurus* was rescored from state 0 to unknown (?) as the only dentary known for *Dolichosaurus longicollis* is too fragmentary to allow evaluation of this character [16].
76. Coronoid: anteromedial margin contacts splenial (0) / anteromedial margin does not contact splenial (1). *Haasiophis* was rescored from state 1 to state 0 [15]; *Pachyrhachis* was rescored from state 1 to unknown (?) because, although the medial aspect of the lower jaw is partially exposed only in the paratype, HUJ-Pal 3775, it is unclear whether the splenial would have been in contact with the coronoid or not in an undistorted jaw [8].
77. Coronoid: ventral margin of medial surface concave (0) / ventral margin of medial surface straight or convex (1).
78. Subcoronoid fenestra on medial surface of the mandible: fenestra present as distinct gap between coronoid and prearticular, surangular exposed in medial view (0) / fenestra absent, prearticular expands dorsally and contacts the entire ventral edge of the coronoid, surangular covered by these elements in medial view (1). *Dolichosaurus* was rescored from state 1 to unknown (?) as this character cannot be evaluated in *Dolichosaurus* [16]; *Pontosaurus lesinensis* was rescored from state 1 to state 0 [11].

79. Surangular: does not form large portion of articular cotyle (0) / forms half of articular cotyle (1).
80. Angular: not exposed, or exposed as only a very narrow splint, on the medial surface of the mandible (0) / with wide exposure on medial surface of the mandible (1). *Dinilysia* was rescored from state 0 to state 1 [12]; *Yurlunggur* was rescored from unknown (?) to state 1 (AP pers. obs. on QMF45391).
81. Prearticular (in medial view with dentary and splenial removed): extends well anterior to coronoid process, past posterior teeth (0) / extends only a short distance in front of coronoid process, not past posterior teeth (1).
82. Adductor fossa: faces dorsomedially (0) / faces dorsally (1). *Pachyrhachis* was rescored from state 1 to unknown (?) because the fossa is not exposed in either of the available specimens [8].
83. Prearticular: not fused with surangular (0) / fused with surangular (compound bone) (1). Modified from Palci & Caldwell[1].
84. Retroarticular process size: short, shorter than articular cotyle (0) / intermediate, between 1 and 2 times articular cotyle (1) / long, over 2 times longer than articular cotyle (2). Modified from Palci & Caldwell[1].
85. Retroarticular process: not tapering, broad distally (0) / tapering, narrow distally (1). Note: not applicable for taxa with a very short retroarticular process. *Dinilysia* was rescored from unknown (?) to inapplicable (-).
86. Marginal teeth: pleurodont, teeth set in a continuous groove (0) / thecodont, teeth ankylosed in discrete alveoli and separated by well-developed interdental plates (1). *Aigialosaurus* was rescored from state 1 to state 0 because, although tooth implantation cannot be assessed in *A. dalmaticus* due to lack of exposure, in *A. bucchichi* the roots of the teeth are clearly exposed medially and there are no interdental plates [6, 7]; *Dolichosaurus* was rescored from state 1 to unknown (?) as no marginal dentition is known for this taxon [16]; *Pontosauurs kornhuberi* was rescored from state 0 to unknown (?) because tooth attachment cannot be evaluated due to lack of exposure in the only specimen [3].
87. Marginal teeth: without high pedestals (0) / with high pedestals (1).
88. Resorption pits: at base of teeth (0) / on bony tooth pedicel (1) / absent (2). *Aigialosaurus* and *Dolichosaurus* were rescored from state 2 to unknown (?) as these states cannot be evaluated in either *Aigialosaurus* [6, 7] or *Dolichosaurus* [16] material.
89. Orientation of replacement teeth: erupt upright, growing straight upwards into functional position (0) / erupt horizontally, and then rotating through ninety degrees about the base into functional position (1). *Dolichosaurus* was rescored from state 1 to unknown (?) as this character cannot be evaluated for this taxon [16].
90. Premaxillary teeth: 6 or more (0) / 2–5 (1) / none (2). *Aigialosaurus* was rescored from state 1 to unknown (?) as a premaxilla is not preserved in either *A. dalmaticus* or *A. bucchichi* [6, 7]; *Eupodophis* and *Haasiophis* were rescored from state 2 to unknown (?) because the premaxilla is not visible in either taxon [9, 13, 14]; *Pachyrhachis* and *Adriosaurus* were rescored from state 1 to unknown (?) because, although a premaxilla is preserved for both taxa, it cannot be determined how many tooth positions were present on its ventral surface [8, 19].
91. Premaxillary teeth (apart from median tooth): similar size or larger than anterior maxillary teeth (0) / distinctly smaller than anterior maxillary teeth (1). *Adriosaurus* was rescored from state 1 to state 0 [19].

92. Palatine teeth: absent (0) / present (1). *Heloderma* was rescored from states 0&1 to state 0 (AP pers. obs. on *H. horridum*); *Dinilysia* was rescored from unknown (?) to state 1 (AP pers. obs. on type specimen MLP 26-410).
93. Palatine teeth: small conical denticles (0) / similar in size to marginal teeth (1).
94. Pterygoid teeth: present (0) / absent (1). *Heloderma* was rescored from state 0 to state 1 (while this state does appear to be polymorphic in *Heloderma suspectum*, in the specimens of *Heloderma horridum* examined there were no pterygoid teeth).
95. Pterygoid teeth: small conical denticles (0) / similar in size to marginal teeth (1). *Xenosaurus* and *Heloderma* were rescored from state 0 to inapplicable (-).
96. Zygosphenes and zygantra: absent (0) / present (1). *Aigialosaurus* was rescored from state 1 to unknown (?) as the presence or absence of these accessory intervertebral articulations cannot be evaluated in either *A. dalmaticus* or *A. bucchichi* [6, 7].
97. Hypapophyses/hypapophyseal peduncles: only extending to the posterior end of the ninth presacral-precloacal vertebra at most (0) / extending to the tenth presacral/precloacal vertebra or beyond (1).
98. Transverse processes of cervicals: on anterior end of centrum (0) / on middle of centrum (1). *Yurlunggur* was rescored from state 1 to state 0 (AP. pers. obs. on QMF45391).
99. Cervical (anterior) intercentra (excluding atlas and axis) as individual ossifications: present (0) / absent (1). For example, intercentra are present as individual ossifications in the anterior (cervical) vertebrae of *Platecarpus tympaniticus*, but are fused to the centrum in *Diploglossus millepunctatus*. Modified from Palci & Caldwell[1].
100. Pachyostosis of mid-dorsal vertebrae and ribs: absent (0) / present (1). *Yurlunggur* was rescored from unknown (?) to state 0 (AP. pers. obs. on QMF45391).
101. Ventral surface of caudal vertebrae: has facets for articulation with intercentra (chevron bones; V- or Y-shaped ossifications) (0) / lacks facets for articulation with intercentra (intercentra absent) (1) / lacks facets for articulation with intercentra (intercentra absent) but has long paired processes (2).
102. Trunk ribs: smoothly curved (0) / middle and distal regions of ribs totally straight (1). In state 0, the body results in a more rounded shape, while state 1 accounts for the laterally compressed condition of the body. *Dinilysia* was rescored from unknown (?) to state 0 (AP pers. obs. on MACN-RN 976); *Pontosaurus lesinensis* was rescored from state 1 to state 0 [11]. Modified from character 107 (‘Body shape’) of Palci & Caldwell[1].
103. Ribs: begin from third (or more anterior) cervical vertebra (0) / begin from fourth (or more posterior) cervical vertebra (1).
104. Distally forked cloacal ribs (‘‘lymphapophyses’’): absent (0) / present (1). *Yurlunggur* was rescored from unknown (?) to state 1 (AP pers. obs. on QMF45391).
105. Tail: cylindrical or only slightly lateral compressed, transverse processes well-developed, chevrons and neural spines not elongated (0) / very laterally compressed, transverse processes reduced anteriorly and absent posteriorly, chevrons and neural spines elongated (1). *Pachyrhachis* was rescored from state 1 to unknown (?) as only the base of the tail is known in *Pachyrhachys* so it is not possible to determine whether the mid and distal portions of the tail were compressed or not [8].
106. Neural spines of posterior caudal vertebrae: projecting dorsally or posterodorsally (0) / projecting almost horizontally, highly inclined posteriorly (1). *Yurlunggur* was rescored from unknown (?) to state 0 (AP pers. obs. on QMF45391).

107. Scapulocoracoid: present and large (0) / present but reduced (1) / absent (2).
108. Anterior (primary) coracoid emargination: present (0) / absent (1).
109. Clavicle: present (0) / absent (1). *Dolichosaurus* was rescored from unknown (?) to state 0[16].
110. Interclavicle: present (0) / absent (1). *Dolichosaurus* was rescored from state 1 to unknown (?)[16].
111. Interclavicle: cross-shaped, with lateral processes (0) / simple rod, without lateral processes (1).
112. Calcified sternum: present (0) / absent (1). *Aigialosaurus* was rescored from state 0 to unknown (?) because a calcified sternum is not preserved in either *A. dalamticus* or *A. bucchichi*[6, 7].
113. Number of rib attachment points to sternum: five pairs (0); four pairs (1); three pairs (2); two pairs or fewer (3). *Aigialosaurus* was rescored from state 2 to unknown (?) because the number of attachment points for ribs on the sternum cannot be determined in either *A. dalamticus* or *A. bucchichi*[6, 7]. Modified from Palci & Caldwell[1].
114. Forelimbs: large (0) / small, humerus as long as or shorter than two dorsal vertebrae (1) / absent (2). *Yurlunggur* was rescored from state 2 to unknown (?): although it is likely that *Yurlunggur* lacked forelimbs, their presence cannot be excluded from disarticulated remains only.
115. Ectepicondylar foramen of humerus: present (0) / absent (1).
116. Pelvis: present and large (0) / present and small (1) / absent (2).
117. Supracetabular iliac process: has the size of a tubercle or spine, being short to very reduced (0) / is elongated and rod-like (1). The supracetabular iliac process departs from the anterodorsal margin of the iliac shaft; state 0 accounts for the condition seen in most extant terrestrial lizards (e.g., *Varanus*, *Xenosaurus*, *Shinisaurus*) and basal mosasauroids (e.g., *Tethysaurus*, *Pannoniasaurus*) while state 1 accounts for the modified condition in derived mosasaurids (e.g., *Mosasaurus*, *Platecarpus*) where the anterodorsal process is elongated and more or less cylindrical (in some mosasaurid taxa not included in this dataset the process can also be dorsoventrally compressed, like for example in *Tylosaurus*). *Aigialosaurus* is scored as ?, because the presence of a supracetabular tubercle cannot be verified due to the way the ilium is exposed; the anteroventrally extended process visible in *A. dalmaticus* and described by Dutchak & Caldwell[6] is instead the preacetabular process departing from the anterior margin of the iliac shaft and not homologous to the supracetabular tubercle (both processes are present in most extant lizards, like for instance *Varanus* and *Iguana*) (IP pers. obs.). New character.
118. Pubis: without expanded plate-like proximal end (0) / with expanded plate-like proximal end (1). For example, *Varanus bengalensis* has state 0, while *Acteosaurus tommasinii* has state 1. Modified from Palci & Caldwell[1].
119. Hindlimbs: present (0) / absent or vestigial (i.e., only femur clearly recognizable inside rib cage) (1).
120. Distal end of tibia: with notch fitting into a ridge on astragalocalcaneum (0) / gently convex for astragalocalcaneal articulation (1).
121. Astragalus and calcaneum: co-ossified (0) / separate (1).
122. Fifth metatarsal: hooked (0) / not hooked (1). *Pachyrhachis* was rescored from state 1 to unknown (?) as no metatarsals are preserved for this taxon [20].

123. Dorsal body osteoderms: present (0) / absent (1). *Yurlunggur* was rescored from state 1 to unknown (?): although it is likely that *Yurlunggur* lacked osteoderms, their presence cannot be excluded from the available disarticulated material.
124. Separable cranial osteoderms: present over entire skull table (0) / absent (1).
125. Scleral ossicles: present (0) / absent (1).
126. Epiphyses on skull and axial skeleton: present (0) / absent (1).
127. Epiphyses on appendicular skeleton: present (0) / absent (1). In *Tetrapodophis* the absence of epyphises is possibly due to its likely early ontogenetic stage; therefore this taxon was conservatively scored as unknown (?) for this character.
128. Posterodistal process of fibula: weakly developed to absent (0) / strongly developed, triangular process extends posteriorly beyond calcaneum (1).
129. Opening for trigeminal nerve: not subdivided for exits of V2 and V3 branches of trigeminal nerve (0) / subdivided for exits of V2 and V3 branches of trigeminal nerve (laterosphenoid bridge present) (1). New character.

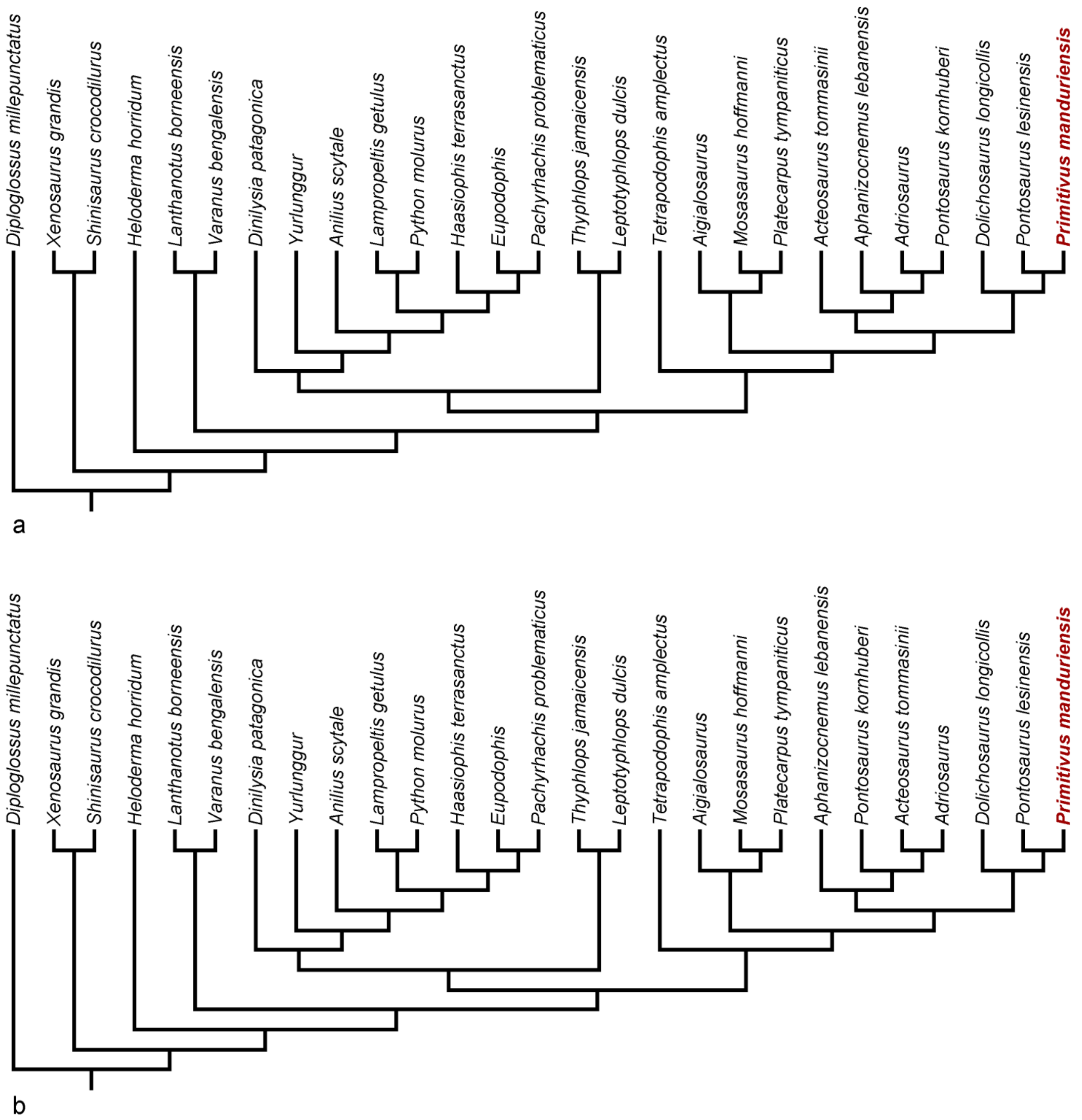


**Supplementary Figure S11.** Optimal trees resulted from the MP analysis. The heuristic search for the MP analysis was performed using the TBR algorithm, and applying two successive rounds of tree searching and swapping. The first round resulted in 967 suboptimal trees (or simply suboptimals), while the second round overflowed the number of Max Trees (99,999). After removing the suboptimals, two optimal trees were retained, and the only difference between them is the arrangement of the clade *Aphanizocnemus* + *Pontosaurus kornhuberi* + *Acteosaurus* + *Adriosaurus*: in one case, *Adriosaurus* and *P. kornhuberi* are sister taxa, with *Aphanizocnemus* at their base, and *Acteosaurus* as the most basal member of the clade (a); alternatively, *Aphanizocnemus* is recovered as the most basal taxon, and *Acteosaurus* + *Adriosaurus* as the most deeply nested, with *P. kornhuberi* in sister relationship to them (b). The length of the two optimal trees is equal to 283 steps, with a consistency index (CI) of 0.53497 and a retention index (RI) of 0.80149.

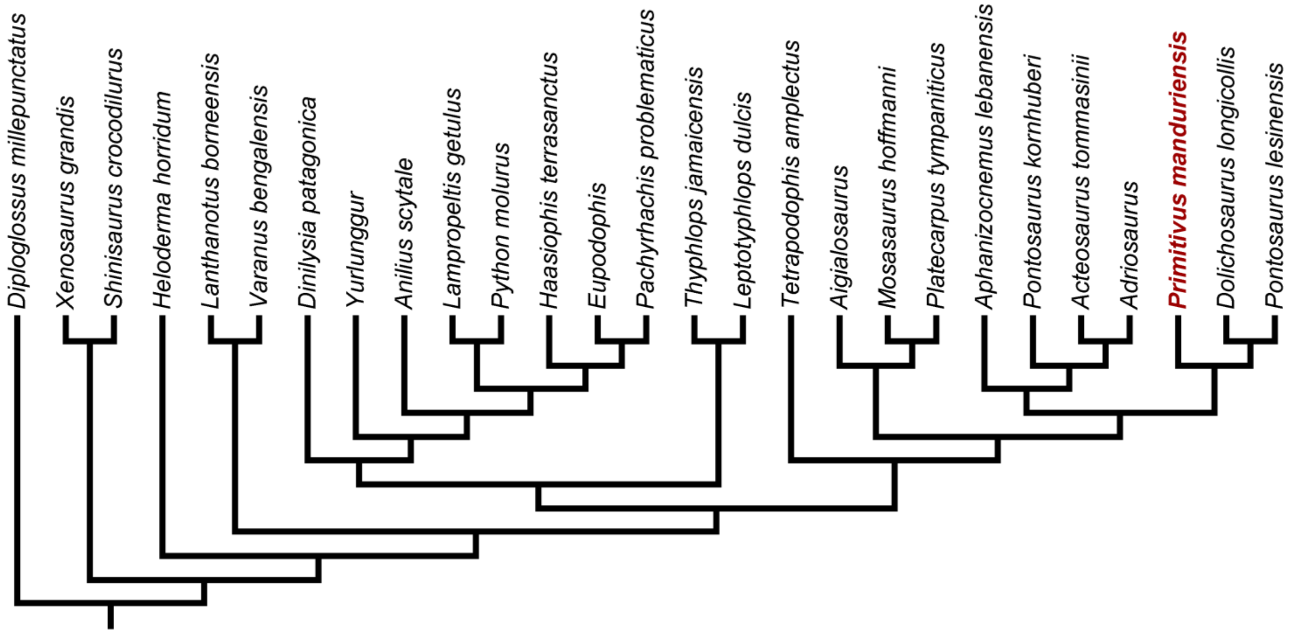


**Supplementary Figure S12.** Single optimal tree resulted from the IWMP analysis. For the IWMP analysis we followed the same procedure as the MP method, enabling the implied weighting option (under K = 3) before running the TBR heuristic search. 271 suboptimal trees were found with the first round of TBR, while the successive swapping of these trees overflowed the limit of 99,999 Max Trees. Only one shortest tree is retained after removing all the suboptimals, corroborating with a better resolution the hypothesis offered by the MP strict consensus of *Primitivus* forming a clade with *Dolichosaurus* and *P. lesinensis*, and *Tetrapodophis* at the stem of a clade consisting of Mosasauroidea (aigialosaurs + mosasaurids) + Dolichosauridae.

## List of apomorphies from the MP analysis

Here we report the list of apomorphies derived from the equal-weight maximum parsimony analysis (strict consensus tree). The corresponding number of the character is given in brackets, and the node numbers are given in the figure below.

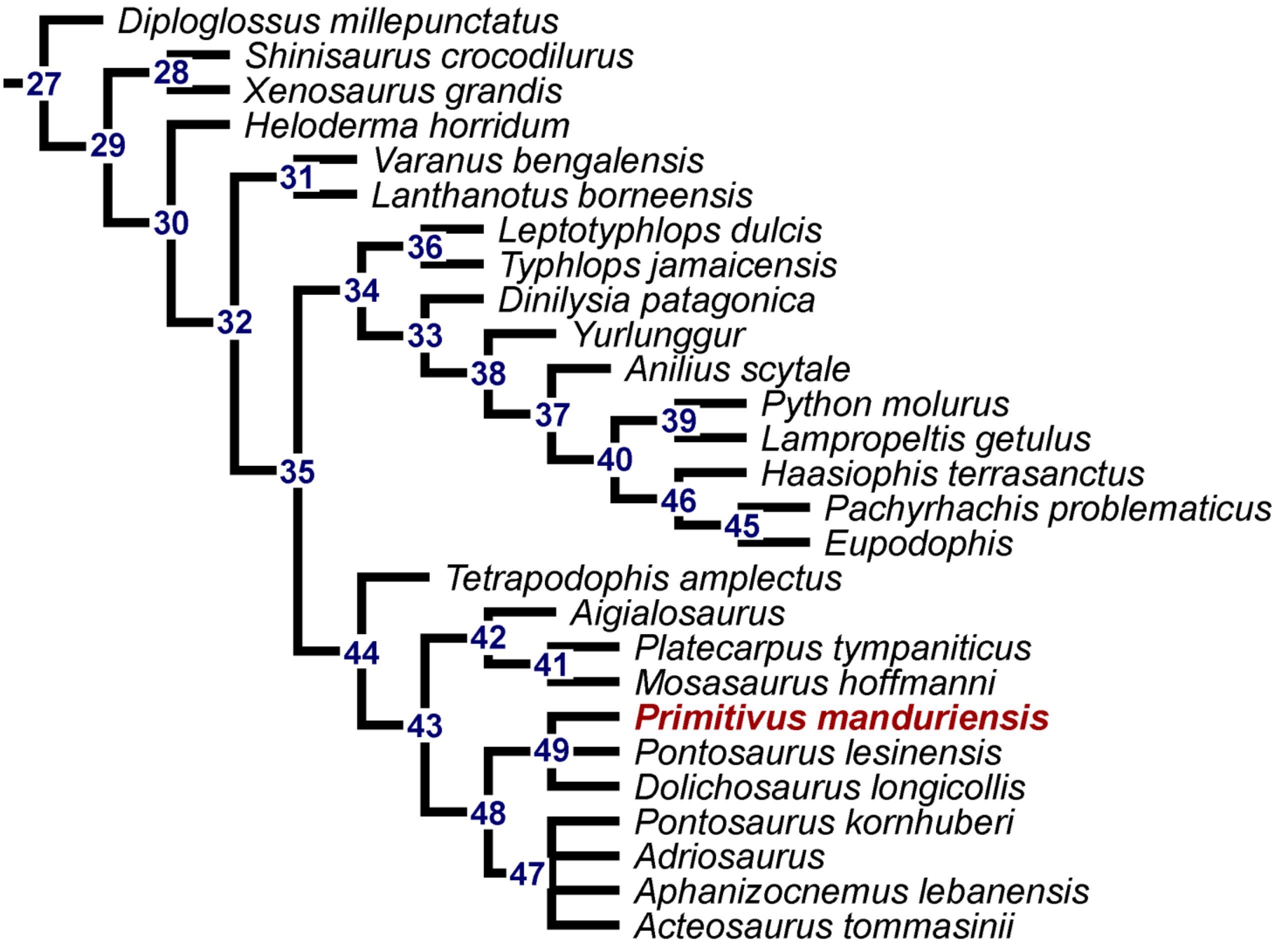


***Diploglossus millepunctatus***
No autapomorphies

***Xenosaurus grandis***
squamosal_dorsal_proc. (26): 0 --> 1
post.opening_vidian_canal_1 (45): 0 --> 1

***Shinisaurus crocodilurus***
parietal-jaw_muscles (22): 0 --> 1
pterygoid_teeth (94): 1 --> 0

***Heloderma horridum***
frontal-orbit (15): 0 --> 1
frontal_ventromedial_proc. (35): 0 --> 1
splenial_size (68): 0 --> 1
coracoid_anterior_emargination (108): 0 --> 1
interclavicle_shape (111): 0 --> 1

***Lanthanotus borneensis***
frontal-orbit (15): 0 --> 1
splenial_size (68): 0 --> 1
palatine_teeth (92): 0 --> 1
pterygoid_teeth (94): 1 --> 0

***Varanus bengalensis***
pineal_foramen (21): 1 --> 0
frontal_ventromedial_proc. (35): 0 --> 1
crista_prootica (41): 1 --> 0
opisthotic_flange (47): 0 --> 1

***Dinilysia patagonica***
basipterygoid_proc. (43): 1 --> 0
Meckel's_canal (64): 1 --> 0
anteromedial_coronoid-splenial (76): 0 --> 1
adductor_fossa (82): 0 --> 1

***Typhlops jamaicensis***
palatine-vomer (54): 0 --> 1
angular (80): 1 --> 0
Retroarticular_proc._size (84): 0 --> 2

***Leptotyphlops dulcis***
pmx_foramina (3): 0 --> 1
nasals_fused/unfused (12): 0 --> 1
dentary_mental_foramina (61): 0 --> 1
splenial_size (68): 0 --> 1
splenial-angular_2 (73): 0 --> 1
anteromedial_coronoid-splenial (76): 0 --> 1
marginal_teeth_implantation (86): 1 --> 0

***Anilius scytale***
parietal_post.rami (23): 1 --> 0
dentary_dorsal_margin (62): 1 --> 0
dentary-coronoid_proc. (63): 1 --> 0
ventral_coronoid (77): 1 --> 0
caudal_vertebrae (101): 0 --> 1

***Lampropeltis getulus***
adductor_fossa (82): 0 --> 1
pelvis_size (116): 1 --> 2

***Python molurus***
frontal-orbit (15): 0 --> 1

***Mosasaurus hoffmanni***
frontal-orbit (15): 0 --> 1

***Platecarpus tympaniticus***
No autapomorphies

***Aigialosaurus***
tympanic_crest (32): 0 --> 1
marginal_teeth_implantation (86): 1 --> 0
coracoid_anterior_emargination (108): 0 --> 1
pelvis_size (116): 1 --> 0

***Eupodophis***
Meckel's_canal (64): 1 --> 0

***Haasiophis terrasanctus***
nasals_size (11): 0 --> 1

***Pachyrhachis problematicus***
No autapomorphies

***Yurlunggur***
external_naris (13): 1 --> 2
frontal-orbit (15): 0 --> 1
opisthotic_flange (47): 0 --> 1
smx_median_flange (52): 1 --> 0

***Acteosaurus tommasinii***
post.caudal_neural_spines (106): 1 --> 0
clavicle (109): 0 --> 1
sternum (112): 0 --> 1

***Aphanizocnemus lebanensis***
No autapomorphies

***Adriosaurus***
frontal_fused/unfused (14): 0 --> 1
pachyostosis_vertebrae_&_ribs (100): 0 --> 1
clavicle (109): 0 --> 1

***Dolichosaurus longicollis***
nasals_size (11): 1 --> 0

***Pontosaurus kornhuberi***
Retroarticular_proc._size (84): 1 --> 2
pachyostosis_vertebrae_&_ribs (100): 0 --> 1

***Pontosaurus lesinensis***
supratemporal (29): 0 --> 1
pachyostosis_vertebrae_&_ribs (100): 0 --> 1
sternum (112): 0 --> 1

***Primitivus manduriensis***
supraoccipital-parietal_1 (48): 2 --> 0
forelimbs (114): 1 --> 0
pelvis_size (116): 1 --> 0

***Tetrapodophis amplectus***
splenial_posterior_extension (69): 2 --> 0
splenial-dentary (71): 1 --> 0
ribs_curved/straight (102): 0 --> 1
post.caudal_neural_spines (106): 0 --> 1

**Node 28 (*Shinisaurus + Xenosaurus*)**
pmx_foramina (3): 0 --> 1
jugal_dermal_sculpture (10): 0 --> 1
frontal_fused/unfused (14): 1 --> 0
palpebral_ossification (18): 1 --> 0
pineal_foramen (21): 1 --> 0

temporal_arch_crest (25): 0 --> 1
ectopterygoid-cheek (56): 0 --> 1

**Node 29 ((*Shinisaurus* + *Xenosaurus*) + (*Heloderma* + ((*Varanus* + *Lanthanotus*) + Pythonomorpha)))**
No synapomorphies

**Node 30 (*Heloderma* + ((*Varanus* + *Lanthanotus*) + Pythonomorpha))**
dentary-coronoid_proc. (63): 0 --> 1
subdental_shelf (65): 1 --> 2
dentary-postdentary (67): 0 --> 1
splenial-dentary (71): 0 --> 1
angular (80): 0 --> 1
Retroarticular_proc._size (84): 2 --> 1
resorption_pits (88): 0 --> 2
pmx_teeth_size (91): 0 --> 1

**Node 31 (*Varanus* + *Lanthanotus*)**
pmx_foramina (3): 0 --> 1
lacrimal_foramen (7): 0 --> 1
posterior_jugal (9): 0 --> 1
nasals_fused/unfused (12): 0 --> 1
palpebral_ossification (18): 1 --> 0
prearticular_medial_view (81): 0 --> 1

**Node 32 ((*Varanus* + *Lanthanotus*) + Pythonomorpha)**
pmx-palatal_ramus (2): 0 --> 1
external_naris (13): 0 --> 2
post.opening_vidian_canal_1 (45): 0 --> 1
anterolateral_coronoid (75): 0 --> 1

**Node 33 (*Dinilysia* + (*Yurlunggur* + (*Anilius* + ((*Python* + *Lampropeltis*) + (*Haasiophis* + (*Pachyrhachis* + *Eudophis*))))))**
pmx-mx (4): 0 --> 1
palatine_teeth (92): 0 --> 1
pterygoid_teeth (94): 1 --> 0
hypapophyses (97): 0 --> 1

**Node 34 (Ophidia)**
lacrimal (6): 0 --> 1
external_naris (13): 2 --> 1
frontal-parietal (16): 0 --> 1
parietal_post.rami (23): 0 --> 1
frontal_ventromedial_proc. (35): 0 --> 2
parietal_flange-parabasisphenoid (38): 0 --> 1
optic_foramina (39): 0 --> 1
trigeminal_foramen (40): 0 --> 1
postemporal_fenestra (50): 0 --> 1
Prearticular_surangular (83): 0 --> 1
Retroarticular_proc._size (84): 1 --> 0
forked_cloacal_ribs (104): 0 --> 1
Scapulocoracoid (107): 0 --> 2
clavicle (109): 0 --> 1
interclavicle (110): 0 --> 1
sternum (112): 0 --> 1
hindlimbs (119): 0 --> 1
sclerotics (125): 0 --> 1
appendicular_epiphyses (127): 0 --> 1

**Node 35 (Pythonomorpha)**
supratemporal-prootic (30): 0 --> 1
quadrate_shape (33): 0 --> 1
parietal_flanges (36): 0 --> 1
parietal_flange-prootic (37): 0 --> 1
basipterygoid_proc. (43): 0 --> 1
smx_median_flange (52): 0 --> 1
mandibular_symphysis (60): 0 --> 1
dentary_dorsal_margin (62): 0 --> 1
Meckel's_canal (64): 0 --> 1
subdental_shelf (65): 2 --> 0
ventral_coronoid (77): 0 --> 1
marginal_teeth_implantation (86): 0 --> 1
zygosphenes_&_zygantra (96): 0 --> 1
ribs_begin (103): 1 --> 0
pelvis_size (116): 0 --> 1
distal_tibia (120): 0 --> 1
astragalus-calcaneum (121): 0 --> 1
skull-axial_epiphyses (126): 0 --> 1

**Node 36 (*Leptotyphlops* + *Typhlops*)**
supratemporal (29): 0 --> 1
quadrate-mandible (34): 0 --> 1
post.opening_vidian_canal_1 (45): 1 --> 0
post.opening_vidian_canal_2 (46): 0 --> 1
caudal_vertebrae (101): 0 --> 1

**Node 37 (*Anilius* + ((*Python* + *Lampropeltis*) + (*Haasiophis* + (*Pachyrhachis* + *Eudophis*))))**
subdental_shelf (65): 0 --> 2
separation_ov_V2_and_V3 (129): 0 --> 1

**Node 38 (*Yurlunggur* + (*Anilius* + ((*Python* + *Lampropeltis*) + (*Haasiophis* + (*Pachyrhachis* + *Eudophis*)))))**
palatine-vomer (54): 0 --> 1
palatine_medial_proc. (55): 0 --> 1
dentary_mental_foramina (61): 0 --> 1
palatine_teeth_size (93): 0 --> 1

**Node 39 (*Python* + *Lampropeltis*)**
splenial_size (68): 0 --> 1
caudal_vertebrae (101): 0 --> 2

**Node 40 ((*Python* + *Lampropeltis*) + (*Haasiophis* + (*Pachyrhachis* + *Eudophis*)))**
quadrate_shape (33): 1 --> 2
anteromedial_coronoid (74): 1 --> 0
pterygoid_teeth_size (95): 0 --> 1

**Node 41 (*Mosasaurus* + *Platecarpus*)**
frontal-parietal (16): 0 --> 1
squamosal_dorsal_proc. (26): 0 --> 1
interclavicle_shape (111): 0 --> 1
appendicular_epiphyses (127): 0 --> 1

**Node 42 (Mosasauroidea)**
mandible_subcoronoid_fenestra (78): 0 --> 1
surangular-articular_cotyle (79): 0 --> 1
adductor_fossa (82): 0 --> 1
marginal_teeth_pedestals (87): 0 --> 1
cervical_transv.proc. (98): 0 --> 1

**Node 43 (Mosasauroidea + Dolichosauridae)**
splenial_anterior_tip (70): 0 --> 1
retroarticular_proc_end (85): 1 --> 0
tail_compression (105): 0 --> 1

**Node 44 (*Tetrapodophis* + (Mosasauroidea + Dolichosauridae))**
pmx-frontal (1): 0 --> 1

**Node 45 (*Pachyrhachis* + *Eudophis*)**
ribs_curved/straight (102): 0 --> 1

**Node 46 (*Haasiophis* + (*Pachyrhachis* + *Eudophis*))**
pmx-palatal_ramus (2): 1 --> 0
cervical_intercentra (99): 1 --> 0
pachyostosis_vertebrae_&_ribs (100): 0 --> 1
hindlimbs (119): 1 --> 0

**Node 47 (*Pontosaurus kornhuberi*, *Adriosaurus*, *Aphanizocnemus*, *Acteosaurus*)**
ribs_curved/straight (102): 0 --> 1
post.caudal_neural_spines (106): 0 --> 1
posterodistal_fibula (128): 0 --> 1

**Node 48 (Dolichosauridae)**
pmx-mx (4): 0 --> 1
posterior_jugal (9): 0 --> 1
postorbital_ventral_proc. (20): 0 --> 1
hypapophyses (97): 0 --> 1
Scapulocoracoid (107): 0 --> 1

**Node 49 (*Primitivus*, *Pontosaurus lesinensis*, *Dolichosaurus*)**
Retroarticular_proc._size (84): 1 --> 2
coracoid_anterior_emargination (108): 0 --> 1

## References


1. Palci A, Caldwell MW. Redescription of *Acteosaurus tommasinii* von Meyer, 1860, and a discussion of evolutionary trends within the clade Ophidiomorpha. *J Vert Paleontol* **30**, 94-108 (2010).
2. Scanlon JD. Skull of the large non-macrostomatan snake Yurlunggur from the Australian Oligo-Miocene. *Nature* **439**, 839-842 (2006).
3. Caldwell MW. A new species of *Pontosaurus* (Squamata, Pythonomorpha) from the Upper Cretaceous of Lebanon and a phylogenetic analysis of Pythonomorpha. *Memorie della Società Italiana di Scienze Naturali e Museo Civico di Storia Naturale di Milano* **34**, 1-43 (2006).
4. Gauthier JA, Estes R, de Queiroz ET. A phylogenetic analysis of Lepidosauromorpha. In: *Phylogenetic Relationships of the Lizard Families* (ed^(eds Estes R, Pregill G). Stanford University Press (1988).
5. Conrad JL. Postcranial skeleton of *Shinisaurus crocodilurus* (Squamata: Anguimorpha). *J Morphol* **267**, 759-775 (2006).
6. Dutchak AR, Caldwell MW. Redescription of *Aigialosaurus dalmaticus* Kramberger, 1892, a Cenomanian mosasauroid lizard from Hvar Island, Croatia. *Can J Earth Sci* **43**, 1821-1834 (2006).

7. Dutchak AR, Caldwell MW. A redescription of *Aigialosaurus* (= *Opetiosaurus*) *bucchichi* (Kornhuber, 1901)(Squamata: Aigialosauridae) with comments on mosasauroid systematics. *J Vert Paleontol* **29**, 437-452 (2009).
8. Caldwell MW. Snake phylogeny, origins, and evolution: the role, impact, and importance of fossils (1869–2006). *Major Transitions in Vertebrate Evolution Indiana University Press, Bloomington and Indianapolis, Indiana*, 253-302 (2007).
9. Palci A, Caldwell MW, Nydam RL. Reevaluation of the anatomy of the Cenomanian (Upper Cretaceous) hind-limbed marine fossil snakes *Pachyrhachis, Haasiophis*, and *Eupodophis*. *J Vert Paleontol* **33**, 1328-1342 (2013).
10. Lee MSY, Caldwell MW. Anatomy and relationships of *Pachyrhachis problematicus*, a primitive snake with hindlimbs. *Philos Trans R Soc Lond, Ser B: Biol Sci* **353**, 1521-1552 (1998).
11. Pierce SE, Caldwell MW. Redescription and phylogenetic position of the Adriatic (Upper Cretaceous; Cenomanian) dolichosaur *Pontosaurus lesinensis* (Kornhuber, 1873). *J Vert Paleontol* **24**, 373-386 (2004).
12. Zaher H, Scanferla CA. The skull of the Upper Cretaceous snake Dinilysia patagonica Smith-Woodward, 1901, and its phylogenetic position revisited. *Zool J Linn Soc* **164**, 194-238 (2012).
13. Rage J-C, Escuillié F. Un nouveau serpent bipède du Cénomanien (Crétacé). Implications phylétiques. *Comptes Rendus de l'Académie des Sciences-Series IIA-Earth and Planetary Science* **330**, 513-520 (2000).
14. Rieppel O, Head JJ. *New Specimens of the Fossil Snake Genus" Eupodophis" Rage & Escuillié, from Cenomanian (late Cretaceous) of Lebanon*. Società italiana di scienze naturali (2004).
15. Rieppel O, Zaher H, Tchernov E, Polcyn MJ. The anatomy and relationships of *Haasiophis terrasanctus*, a fossil snake with well-developed hind limbs from the mid-Cretaceous of the Middle East. *J Paleontol* **77**, 536-558 (2003).
16. Caldwell MW. On the aquatic squamate *Dolichosaurus longicollis* owen, 1850 (Cenomanian, Upper Cretaceous), and the evolution of elongate necks in squamates. *J Vert Paleontol* **20**, 720-735 (2000).
17. Estes R, de Queiroz K, Gauthier JA. Phylogenetic relationships within Squamata. In: *Phylogenetic Relationships of the Lizard Families* (ed^(eds Estes R, Pregill G). Stanford University Press (1988).
18. McDowell SB, Bogert CM. The systematic position of Lanthanotus and the affinities of the anguinomorphan lizards. *Bull Am Mus Nat Hist* **105**,  (1954).
19. Lee MSY, Caldwell MW. *Adriosaurus* and the affinities of mosasaurs, dolichosaurs and snakes. *J Paleontol* **74**, 915-937 (2000).
20. Caldwell MW, Lee MSY. A snake with legs from the marine Cretaceous of the Middle East. *Nature* **386**, 705-709 (1997).
