# Supplementary material for: A new fossil marine lizard with soft tissues from the Late Cretaceous of southern Italy
Source: R Soc Open Sci. 2018 Jun 20;5(6):172411. doi: 10.1098/rsos.172411 (PMC6030324; doi:10.1098/rsos.172411)
Supplement: Bayesian Inference results [file rsos172411supp5.pdf]

```
Logging screen output to file "primitivus.log.txt"
Nst =1 unchanged because dataType is not DNA or RNA
Setting Rates to Gamma
Setting Ngammacat to 8
Enabling Coding Variable
Successfully set likelihood model parameters
Unlinking
Setting Ratepr to Variable [Dirichlet(..,1,..)] for partition 1
Setting Shapepr to Exponential(1.00) for partition 1
Successfully set prior model parameters
Setting number of generations to 10000000
Using relative burnin (a fraction of samples discarded).
Setting burnin fraction to 0.25
Setting print frequency to 1000
Setting sample frequency to 1000
Setting number of chains to 4
Setting heating parameter to 0.010000
Successfully set chain parameters
Running Markov chain
MCMC stamp = 3520932144
Seed = 1839578841
Swapseed = 1525277089
Model settings:

  Data not partitioned --
    Datatype  = Standard
    Coding    = Variable
    # States  = Variable, up to 10
                State frequencies are fixed to be equal
    Rates     = Gamma
                The distribution is approximated using 8 categories.
                Likelihood summarized over all rate categories in each generation.
                Shape parameter is exponentially
                distributed with parameter (1.00).

Active parameters:

  Parameters
  ---------------------
  Statefreq          1
  Shape              2
  Ratemultiplier     3
  Topology           4
  Brlens             5
  ---------------------

  1 --  Parameter  = Alpha_symdir
        Type       = Symmetric diricihlet/beta distribution alpha_i parameter
        Prior      = Symmetric dirichlet with all parameters equal to infinity

  2 --  Parameter  = Alpha
        Type       = Shape of scaled gamma distribution of site rates
        Prior      = Exponential(1.00)
```

3 -- Parameter = Ratemultiplier
Type   = Partition-specific rate multiplier
Prior   = Dirichlet(1.00)

4 -- Parameter = Tau
Type   = Topology
Prior   = All topologies equally probable a priori
Subparam. = V

5 -- Parameter = V
Type   = Branch lengths
Prior   = Unconstrained:GammaDir(1.0,0.1000,1.0,1.0)

The MCMC sampler will use the following moves:
With prob. Chain will use move
1.90 %   Multiplier(Alpha)
1.43 %   Dirichlet(Ratemultiplier)
1.43 %   Slider(Ratemultiplier)
9.52 %   ExtSPR(Tau,V)
9.52 %   ExtTBR(Tau,V)
9.52 %   NNI(Tau,V)
9.52 %   ParsSPR(Tau,V)
38.10 %   Multiplier(V)
13.33 %   Nodeslider(V)
5.71 %   TLMultiplier(V)

Division 1 has 131 unique site patterns
Initializing conditional likelihoods
Using standard non-SSE likelihood calculator for division 1 (single-precision)

Initial log likelihoods and log prior probs for run 1:
Chain 1 -- -2235.129577 -- 70.937568
Chain 2 -- -2301.383273 -- 70.937568
Chain 3 -- -2315.489061 -- 70.937568
Chain 4 -- -2407.024110 -- 70.937568

Initial log likelihoods and log prior probs for run 2:
Chain 1 -- -2215.433675 -- 70.937568
Chain 2 -- -2316.032842 -- 70.937568
Chain 3 -- -2272.055205 -- 70.937568
Chain 4 -- -2163.714225 -- 70.937568

Using a relative burnin of 25.0 % for diagnostics

Average standard deviation of split frequencies: 0.004129

Analysis completed in 5 hours 43 mins 12 seconds
Analysis used 20591.61 seconds of CPU time
Likelihood of best state for "cold" chain of run 1 was -993.38

Likelihood of best state for "cold" chain of run 2 was -992.92

Acceptance rates for the moves in the "cold" chain of run 1:

With prob.	(last 100)	chain accepted proposals by move
28.4 %	(22 %)	Multiplier(Alpha)
16.6 %	(25 %)	Dirichlet(Ratemultiplier)
32.5 %	(21 %)	Slider(Ratemultiplier)
16.9 %	(16 %)	ExtSPR(Tau,V)
13.8 %	(10 %)	ExtTBR(Tau,V)
23.8 %	(19 %)	NNI(Tau,V)
9.9 %	(4 %)	ParsSPR(Tau,V)
26.5 %	(22 %)	Multiplier(V)
50.6 %	(59 %)	Nodeslider(V)
25.6 %	(29 %)	TLMultiplier(V)

Acceptance rates for the moves in the "cold" chain of run 2:

With prob.	(last 100)	chain accepted proposals by move
28.6 %	(29 %)	Multiplier(Alpha)
17.2 %	(1 %)	Dirichlet(Ratemultiplier)
32.5 %	(20 %)	Slider(Ratemultiplier)
17.0 %	(16 %)	ExtSPR(Tau,V)
13.8 %	(15 %)	ExtTBR(Tau,V)
23.9 %	(32 %)	NNI(Tau,V)
10.0 %	(5 %)	ParsSPR(Tau,V)
25.5 %	(29 %)	Multiplier(V)
50.5 %	(56 %)	Nodeslider(V)
25.6 %	(16 %)	TLMultiplier(V)

Chain swap information for run 1:

	1	2	3	4
1 |		0.86	0.71	0.56
2 |	1668219		0.85	0.69
3 |	1667055	1664333		0.83
4 |	1668878	1665771	1665744	

Chain swap information for run 2:

	1	2	3	4
1 |		0.86	0.70	0.55
2 |	1666837		0.84	0.68
3 |	1665943	1668873		0.83
4 |	1665828	1666157	1666362	

Upper diagonal: Proportion of successful state exchanges between chains
Lower diagonal: Number of attempted state exchanges between chains

Chain information:

ID -- Heat
----
1 -- 1.00 (cold chain)

```
      2 -- 0.99
      3 -- 0.98
      4 -- 0.97

   Heat = 1 / (1 + T * (ID - 1))
      (where T = 0.01 is the temperature and ID is the chain number)
```

Summarizing parameters in files C:\Users\caldwell_lab\Desktop\MrBayes 3.2.6\Primitivus Bayesian\Primitivus_Bayesian.nex.run1.p and C:\Users\caldwell_lab\Desktop\MrBayes 3.2.6\Primitivus Bayesian\Primitivus_Bayesian.nex.run2.p

Writing summary statistics to file C:\Users\caldwell_lab\Desktop\MrBayes 3.2.6\Primitivus Bayesian\Primitivus_Bayesian.nex.pstat

Using relative burnin ('relburnin=yes'), discarding the first 25 % of samples

Below are rough plots of the generation (x-axis) versus the log probability of observing the data (y-axis). You can use these graphs to determine what the burn in for your analysis should be. When the log probability starts to plateau you may be at stationarity. Sample trees and parameters after the log probability plateaus. Of course, this is not a guarantee that you are at stationarity. Also examine the convergence diagnostics provided by the 'sump' and 'sumt' commands for all the parameters in your model. Remember that the burn in is the number of samples to discard. There are a total of ngen / samplefreq samples taken during a MCMC analysis.

Overlay plot for both runs:
(1 = Run number 1; 2 = Run number 2; * = Both runs)

```
   +------------------------------------------------------------+ -1012.88
   |        1     1           *             1  2   |
   |  1   1                 1                     1|
   |   1    21           1           2          2     |
   | 1  2  1                       1    1     1      |
   |      21    1  2         2  2  2       11       1     |
   |   2  2 1    2  *12  1   1        1 2           12  1    |
   |1        22       22  *    1        2  *1 22*        1  |
   |     2              21   2     1      1      2          |
   |2          11 2    1 1     2   1   2                  * |
   | 2           2    1     2 1  2     11        12   2     2|
   |    11      2           2 1         1        12   2    |
   |             1      2        2    2 2  2     12        |
   |  2                     2      1     2             |
   |      2                                   12  |
   |                2         1                       |
   +------+-----+-----+-----+-----+-----+-----+-----+-----+-----+ -1015.08
   ^                                                    ^
   2500000                                              10000000
```

Estimated marginal likelihoods for runs sampled in files
"C:\Users\caldwell_lab\Desktop\MrBayes 3.2.6\Primitivus Bayesian\Primitivus_Bayesian.nex.run1.p" and
"C:\Users\caldwell_lab\Desktop\MrBayes 3.2.6\Primitivus Bayesian\Primitivus_Bayesian.nex.run2.p":
(Use the harmonic mean for Bayes factor comparisons of models)

(Values are saved to the file C:\Users\caldwell_lab\Desktop\MrBayes 3.2.6\Primitivus Bayesian\Primitivus_Bayesian.nex.lstat)

Run	Arithmetic mean	Harmonic mean
1	-1003.55	-1029.60
2	-1004.11	-1034.78
TOTAL	-1003.79	-1034.09

Model parameter summaries over the runs sampled in files
"C:\Users\caldwell_lab\Desktop\MrBayes 3.2.6\Primitivus Bayesian\Primitivus_Bayesian.nex.run1.p" and "C:\Users\caldwell_lab\Desktop\MrBayes 3.2.6\Primitivus Bayesian\Primitivus_Bayesian.nex.run2.p":
Summaries are based on a total of 15002 samples from 2 runs.
Each run produced 10001 samples of which 7501 samples were included.
Parameter summaries saved to file "C:\Users\caldwell_lab\Desktop\MrBayes 3.2.6\Primitivus Bayesian\Primitivus_Bayesian.nex.pstat".

			95% HPD Interval					
Parameter	Mean	Variance	Lower	Upper	Median	min ESS*	avg ESS	PSRF+
TL	7.413377	20.783788	2.820522	16.348190	5.934442	2182.87	2319.56	1.000
alpha	2.617121	1.387935	0.753614	4.973965	2.412317	6576.98	6837.42	1.000
m{1}	0.720584	0.065417	0.171595	1.006238	0.745245	2322.97	2409.64	1.000

* Convergence diagnostic (ESS = Estimated Sample Size); min and avg values correspond to minimal and average ESS among runs.
ESS value below 100 may indicate that the parameter is undersampled.
+ Convergence diagnostic (PSRF = Potential Scale Reduction Factor; Gelman and Rubin, 1992) should approach 1.0 as runs converge.

Summarizing trees in files "C:\Users\caldwell_lab\Desktop\MrBayes 3.2.6\Primitivus Bayesian\Primitivus_Bayesian.nex.run1.t" and "C:\Users\caldwell_lab\Desktop\MrBayes 3.2.6\Primitivus Bayesian\Primitivus_Bayesian.nex.run2.t"
Using relative burnin ('relburnin=yes'), discarding the first 25 % of sampled trees
Writing statistics to files C:\Users\caldwell_lab\Desktop\MrBayes 3.2.6\Primitivus Bayesian\Primitivus_Bayesian.nex.<parts|tstat|vstat|trprobs|con>
Examining first file ...
Found one tree block in file "C:\Users\caldwell_lab\Desktop\MrBayes 3.2.6\Primitivus Bayesian\Primitivus_Bayesian.nex.run1.t" with 10001 trees in last block
Expecting the same number of trees in the last tree block of all files

Tree reading status:

```
0      10      20      30      40      50      60      70      80      90     100
v-------v-------v-------v-------v-------v-------v-------v-------v-------v-------v
*********************************************************************************
```

Read a total of 20002 trees in 2 files (sampling 15002 of them)

(Each file contained 10001 trees of which 7501 were sampled)

List of taxa in bipartitions:

1 -- Diploglossusmillepunctatus
2 -- Xenosaurusgrandis
3 -- Shinisauruscrocodilurus
4 -- Helodermahorridum
5 -- Lanthanotusborneensis
6 -- Varanusbengalensis
7 -- Dinilysiapatagonica
8 -- Thyphlopsjamaicensis
9 -- Leptotyphlopsdulcis
10 -- Aniliusscytale
11 -- Lampropeltisgetulus
12 -- Pythonmolurus
13 -- Mosasaurushoffmanni
14 -- Platecarpustympaniticus
15 -- Aigialosaurus
16 -- Eupodophis
17 -- Haasiophisterrasanctus
18 -- Pachyrhachisproblematicus
19 -- Yurlunggur
20 -- Acteosaurustommasinii
21 -- Aphanizocnemuslebanensis
22 -- Adriosaurus
23 -- Dolichosauruslongicollis
24 -- Pontosauruskornhuberi
25 -- Pontosauruslesinensis
26 -- Primitivusmanduriensis
27 -- Tetrapodophisamplectus

Summary statistics for informative taxon bipartitions
(saved to file "C:\Users\caldwell_lab\Desktop\MrBayes 3.2.6\Primitivus Bayesian\Primitivus_Bayesian.nex.tstat"):

ID	#obs	Probab.	Sd(s)+	Min(s)	Max(s)	Nruns
28	15002	1.000000	0.000000	1.000000	1.000000	2
29	14994	0.999467	0.000566	0.999067	0.999867	2
30	14934	0.995467	0.000943	0.994801	0.996134	2
31	14699	0.979803	0.000848	0.979203	0.980403	2
32	14601	0.973270	0.000660	0.972804	0.973737	2
33	13411	0.893947	0.001225	0.893081	0.894814	2
34	13274	0.884815	0.004525	0.881616	0.888015	2
35	11420	0.761232	0.000754	0.760699	0.761765	2
36	9386	0.625650	0.011878	0.617251	0.634049	2
37	9267	0.617718	0.000283	0.617518	0.617918	2
38	8650	0.576590	0.014329	0.566458	0.586722	2
39	8622	0.574723	0.003959	0.571924	0.577523	2
40	8206	0.546994	0.001131	0.546194	0.547794	2
41	7893	0.526130	0.008955	0.519797	0.532462	2
42	7856	0.523664	0.008673	0.517531	0.529796	2
43	7603	0.506799	0.010087	0.499667	0.513931	2

44	7380	0.491934	0.004148	0.489001	0.494867	2
45	6418	0.427810	0.012632	0.418877	0.436742	2
46	6030	0.401946	0.004148	0.399013	0.404879	2
47	6002	0.400080	0.007164	0.395014	0.405146	2
48	5690	0.379283	0.006033	0.375017	0.383549	2
49	5392	0.359419	0.005845	0.355286	0.363552	2
50	5264	0.350887	0.001320	0.349953	0.351820	2
51	5198	0.346487	0.000754	0.345954	0.347020	2
52	5125	0.341621	0.006505	0.337022	0.346221	2
53	4252	0.283429	0.004902	0.279963	0.286895	2
54	4060	0.270631	0.005090	0.267031	0.274230	2
55	3516	0.234369	0.001320	0.233436	0.235302	2
56	3430	0.228636	0.001508	0.227570	0.229703	2
57	3322	0.221437	0.000754	0.220904	0.221970	2
58	3210	0.213971	0.003582	0.211438	0.216504	2
59	3036	0.202373	0.000943	0.201706	0.203040	2
60	3029	0.201906	0.007636	0.196507	0.207306	2
61	2998	0.199840	0.003394	0.197440	0.202240	2
62	2581	0.172044	0.003865	0.169311	0.174777	2
63	2306	0.153713	0.002262	0.152113	0.155313	2
64	2202	0.146780	0.001320	0.145847	0.147714	2
65	2199	0.146580	0.002734	0.144647	0.148514	2
66	2085	0.138981	0.001414	0.137982	0.139981	2
67	1912	0.127450	0.002074	0.125983	0.128916	2
68	1877	0.125117	0.006693	0.120384	0.129849	2
69	1860	0.123983	0.003205	0.121717	0.126250	2
70	1818	0.121184	0.000754	0.120651	0.121717	2
71	1616	0.107719	0.002828	0.105719	0.109719	2
72	1593	0.106186	0.007259	0.101053	0.111318	2
73	1522	0.101453	0.002828	0.099453	0.103453	2
74	1509	0.100587	0.001603	0.099453	0.101720	2
75	1484	0.098920	0.008861	0.092654	0.105186	2
76	1452	0.096787	0.008107	0.091055	0.102520	2

+ Convergence diagnostic (standard deviation of split frequencies)
  should approach 0.0 as runs converge.

Summary statistics for branch and node parameters
   (saved to file "C:\Users\caldwell_lab\Desktop\MrBayes 3.2.6\Primitivus Bayesian\Primitivus_Bayesian.nex.vstat"):

			95% HPD Interval				
Parameter	Mean	Variance	Lower	Upper	Median	PSRF+	Nruns
length[1]	0.132596	0.011669	0.000947	0.320661	0.105381	1.000	2
length[2]	0.035166	0.001187	0.000006	0.095690	0.026091	1.000	2
length[3]	0.035348	0.001277	0.000006	0.094757	0.025743	1.000	2
length[4]	0.056285	0.004013	0.000004	0.168899	0.038221	1.000	2
length[5]	0.108679	0.009213	0.000067	0.283488	0.084069	1.000	2
length[6]	0.101882	0.011693	0.000006	0.289993	0.075072	1.000	2
length[7]	0.154218	0.022442	0.000025	0.420169	0.120219	1.000	2
length[8]	0.086435	0.007067	0.000009	0.231389	0.064925	1.000	2
length[9]	0.141959	0.014113	0.003782	0.354001	0.111189	1.000	2

length[10]	0.130293	0.010436	0.009763	0.318934	0.103433	1.000	2
length[11]	0.048007	0.002210	0.000001	0.130421	0.035325	1.000	2
length[12]	0.025167	0.000949	0.000001	0.076720	0.016293	1.000	2
length[13]	0.022139	0.000588	0.000002	0.063400	0.015441	1.000	2
length[14]	0.014906	0.000379	0.000003	0.049039	0.008885	1.000	2
length[15]	0.067945	0.005710	0.000001	0.203487	0.046652	1.000	2
length[16]	0.080356	0.006144	0.000007	0.217970	0.060206	1.000	2
length[17]	0.034164	0.001533	0.000007	0.101976	0.022895	1.000	2
length[18]	0.040267	0.002274	0.000009	0.122382	0.026673	1.000	2
length[19]	0.092225	0.009176	0.000023	0.256360	0.066779	1.000	2
length[20]	0.099498	0.015307	0.000002	0.314622	0.062965	1.000	2
length[21]	0.070908	0.007940	0.000001	0.227942	0.043330	1.000	2
length[22]	0.070087	0.008080	0.000000	0.221858	0.043049	1.000	2
length[23]	0.072836	0.007078	0.000005	0.220890	0.048029	1.000	2
length[24]	0.053105	0.004439	0.000001	0.167351	0.032686	1.000	2
length[25]	0.102741	0.010108	0.000044	0.284231	0.075973	1.000	2
length[26]	0.114670	0.016957	0.000011	0.351689	0.077464	1.000	2
length[27]	0.179258	0.035564	0.000001	0.510973	0.129721	1.000	2
length[28]	1.227768	0.704194	0.319298	2.851872	0.984570	1.000	2
length[29]	0.871259	0.421098	0.156978	2.095164	0.691500	1.000	2
length[30]	0.338549	0.058250	0.073831	0.800576	0.271902	1.000	2
length[31]	0.128166	0.010685	0.002431	0.312428	0.100634	1.000	2
length[32]	0.127453	0.012556	0.000003	0.322306	0.098595	1.000	2
length[33]	0.232335	0.030371	0.000137	0.550296	0.187514	1.000	2
length[34]	0.261489	0.048507	0.000041	0.655491	0.206527	1.000	2
length[35]	0.270569	0.039525	0.000445	0.645789	0.220500	1.000	2
length[36]	0.104162	0.008781	0.000174	0.261597	0.081316	1.000	2
length[37]	0.143869	0.014726	0.001895	0.364144	0.112588	1.000	2
length[38]	0.207447	0.036249	0.000788	0.541796	0.157912	1.000	2
length[39]	0.053297	0.002758	0.000009	0.152948	0.038448	1.000	2
length[40]	0.207087	0.048395	0.000301	0.596393	0.145394	1.000	2
length[41]	0.216603	0.041453	0.000200	0.593037	0.162360	1.000	2
length[42]	0.130175	0.012299	0.000201	0.333516	0.101791	1.000	2
length[43]	0.165921	0.022354	0.000053	0.433189	0.128797	1.000	2
length[44]	0.098754	0.008967	0.000004	0.258356	0.075407	1.001	2
length[45]	0.136581	0.017373	0.000228	0.360573	0.101489	1.000	2
length[46]	0.151815	0.025989	0.000028	0.425909	0.108213	1.000	2
length[47]	0.145079	0.023293	0.000067	0.409735	0.104278	1.000	2
length[48]	0.241307	0.040811	0.000091	0.600979	0.198071	1.000	2
length[49]	0.038722	0.001812	0.000009	0.109436	0.027360	1.000	2
length[50]	0.130224	0.014844	0.000016	0.350508	0.100192	1.000	2
length[51]	0.135266	0.020573	0.000108	0.372176	0.098838	1.000	2
length[52]	0.100434	0.011555	0.000018	0.281127	0.071600	1.000	2
length[53]	0.129101	0.011796	0.000834	0.327916	0.102901	1.000	2
length[54]	0.053351	0.003635	0.000029	0.153324	0.037606	1.000	2
length[55]	0.056188	0.003554	0.000018	0.163733	0.039526	1.000	2
length[56]	0.206797	0.035302	0.000212	0.566079	0.160142	1.000	2
length[57]	0.193204	0.026975	0.000004	0.494441	0.157440	1.000	2
length[58]	0.152044	0.022323	0.000284	0.421984	0.111905	1.000	2
length[59]	0.038083	0.002219	0.000019	0.114629	0.025168	1.000	2
length[60]	0.033607	0.001772	0.000015	0.102560	0.021962	1.000	2
length[61]	0.058610	0.002868	0.000097	0.161388	0.044681	1.000	2
length[62]	0.094567	0.009145	0.000027	0.270295	0.069744	1.000	2
length[63]	0.123744	0.013293	0.000905	0.349662	0.092375	1.000	2

length[64]	0.103245	0.015260	0.000002	0.304675	0.068063	1.000	2
length[65]	0.226707	0.052546	0.000215	0.660057	0.165414	1.001	2
length[66]	0.064283	0.006338	0.000006	0.206383	0.039614	1.000	2
length[67]	0.045228	0.003038	0.000054	0.128655	0.030406	1.000	2
length[68]	0.078090	0.007272	0.000080	0.232274	0.054342	1.000	2
length[69]	0.132470	0.020398	0.000050	0.403879	0.093103	1.000	2
length[70]	0.088616	0.011340	0.000054	0.268016	0.058229	0.999	2
length[71]	0.092996	0.013915	0.000054	0.278477	0.059517	0.999	2
length[72]	0.061809	0.003615	0.000082	0.167746	0.045981	1.000	2
length[73]	0.056271	0.004090	0.000048	0.164664	0.037868	1.000	2
length[74]	0.123857	0.022405	0.000000	0.375057	0.082187	1.001	2
length[75]	0.113910	0.013990	0.000002	0.328471	0.084550	1.000	2
length[76]	0.084175	0.008953	0.000097	0.258390	0.055159	0.999	2

+ Convergence diagnostic (PSRF = Potential Scale Reduction Factor; Gelman
  and Rubin, 1992) should approach 1.0 as runs converge. NA is reported when
  deviation of parameter values within all runs is 0 or when a parameter
  value (a branch length, for instance) is not sampled in all runs.

Summary statistics for partitions with frequency >= 0.10 in at least one run:
    Average standard deviation of split frequencies = 0.004129
    Maximum standard deviation of split frequencies = 0.014329
    Average PSRF for parameter values (excluding NA and >10.0) = 1.000
    Maximum PSRF for parameter values = 1.001

Clade credibility values:

Subtree rooted at node 40:

```
   /------------------------------------------------------ Varanusbengalen~ (6)
   |
   |                          /--------------------------- Dinilysiapatago~ (7)
   |                          |
   |                          |                    /----- Thyphlopsjamaic~ (8)
   |                          |         /----100---+
   |                          |         |          \----- Leptotyphlopsdu~ (9)
   |                          |         |
   |                          |         |---------------- Aniliusscytale (10)
   |                    /-100-+         |
   |                    |     |         |---------------- Lampropeltisge~ (11)
   |                    |     |    /-62-+
   |                    |     |    |    |---------------- Pythonmolurus (12)
   |                    |     |    |    |
   |                    |     |    |    |          /----- Eupodophis (16)
   |                    |     |    |    |    /--57-+
   |               /-55-+     \-51-+    |    |     \----- Pachyrhachispr~ (18)
   |               |    |          |    \-63-+
--52-+             |    |          |         \----------- Haasiophisterr~ (17)
   |               |    |          |
   |          /-58-+    |          \--------------------- Yurlunggur (19)
   |          |    |    |
   |          |    |    \-------------------------------- Acteosaurustom~ (20)
```

```
   |         |    |
   |         |    \------------------------------------- Adriosaurus (22)
   |         |
   |         |                         /----- Mosasaurushoff~ (13)
   |         |                    /--97-+
   |         |                    |    \----- Platecarpustym~ (14)
   |         |--------------88--------------+
   |    /-53-+                         \----------- Aigialosaurus (15)
   |    |    |
   |    |    |------------------------------------------ Aphanizocnemus~ (21)
   |    |    |
   |    |    |------------------------------------------ Dolichosaurusl~ (23)
   |    |    |
   \-100-+    |------------------------------------------ Pontosauruskor~ (24)
        |    |
        |    |------------------------------------------ Pontosaurusles~ (25)
        |    |
        |    \------------------------------------------ Tetrapodophisa~ (27)
        |
        \----------------------------------------------- Primitivusmand~ (26)
```

Root part of tree:

```
/------------------------------------------------------------ Diploglossusmil~ (1)
|
|                          /-------------------- Xenosaurusgrand~ (2)
|------------------98------------------+
+                           \-------------------- Shinisauruscroc~ (3)
|
|             /---------------------------------------- Helodermahorrid~ (4)
|             |
\--------89--------+                  /-------------------- Lanthanotusborn~ (5)
              \---------76--------+
                           \-------------------- (40)
```

Phylogram (based on average branch lengths):

```
                                                                                                      /--
Diploglossusmil~ (1)
  |
  | / Xenosaurusgrand~ (2)
  |-+
  | \ Shinisauruscroc~ (3)
  |
  +  /- Helodermahorrid~ (4)
  |  |
  |  |   /-- Lanthanotusborn~ (5)
  |  |   |
  |  |   | /- Varanusbengalen~ (6)
  |  |   | |
  \--+   | |                           /-- Dinilysiapatago~ (7)
     |   | |                           |
     |   | |                           |      /- Thyphlopsjamaic~ (8)
```

```
   |  | |                                  |  /----+
   |  | |                                  |  |    \-- Leptotyphlopsdu~ (9)
   |  | |                                  |  |
   \---+ |                                 |  |-- Aniliusscytale (10)
       | |                      /-----------+  |
       | |                      |           |  |- Lampropeltisge~ (11)
       | |                      |           | /-+
       | |                      |           | | |- Pythonmolurus (12)
       | |                      |           | | |
       | |                      |           | | | /- Eupodophis (16)
       | |                      |           | | | |
       | |                   /--+           \-+ | |- Pachyrhachispr~ (18)
       | |                   |  |             | \-+
       \-+                   |  |             |   \ Haasiophisterr~ (17)
         |                   |  |             |
         |                /--+  |             \- Yurlunggur (19)
         |                |  |  |
         |                |  |  \- Acteosaurustom~ (20)
         |                |  |
         |                |  \- Adriosaurus (22)
         |                |
         |                |    / Mosasaurushoff~ (13)
         |                |  /-+
         |                |  | \ Platecarpustym~ (14)
         |                |---+
         |             /--+   \- Aigialosaurus (15)
         |             |  |
         |             |  |- Aphanizocnemus~ (21)
         |             |  |
         |             |  |- Dolichosaurusl~ (23)
         |             |  |
         \-------------+  |- Pontosauruskor~ (24)
                       |  |
                       |  |-- Pontosaurusles~ (25)
                       |  |
                       |  \--- Tetrapodophisa~ (27)
                       |
                       \-- Primitivusmand~ (26)

|-------| 0.500 expected changes per site

Calculating tree probabilities...

Credible sets of trees (14876 trees sampled):
   50 % credible set contains 7375 trees
   90 % credible set contains 13376 trees
   95 % credible set contains 14126 trees
   99 % credible set contains 14726 trees

Exiting mrbayes block
Reached end of file
```
